# Drug-coated balloons vs. drug-eluting stents for coronary artery disease: an updated systematic review and meta-analysis of randomized controlled trials with lesion-specific insights

**DOI:** 10.3389/fcvm.2026.1843262

**Published:** 2026-05-18

**Authors:** Yuting Wang, Yaqi Shi, Qiong Wang, Shengxiang Yang, Xin Chen

**Affiliations:** Cardiovascular Disease Center, The Central Hospital of Enshi Tujia and Miao Autonomous Prefecture, Enshi Clinical College of Wuhan University, Enshi, Hubei, China

**Keywords:** coronary artery disease, drug-coated balloon, drug-eluting stent, meta-analysis, percutaneous coronary intervention

## Abstract

**Objective:**

To compare the efficacy and safety of drug-coated balloons (DCB) vs. drug-eluting stents (DES) in patients with coronary artery disease (CAD) undergoing percutaneous coronary intervention, with a focus on lesion-specific and presentation-specific outcomes.

**Methods:**

We systematically searched PubMed, Embase, CENTRAL, and Web of Science for randomized controlled trials (RCTs) comparing DCB with DES from inception to March 2026. Outcomes included major adverse cardiac events (MACEs), target lesion revascularization (TLR), device-oriented composite endpoint (DoCE), patient-oriented composite endpoint (PoCE), cardiac death, myocardial infarction (MI), all-cause death, thrombosis (definite/probable), and angiographic endpoints. Pooled odds ratios (ORs) or mean differences (MDs) were calculated using random-effects models. Subgroup analyses were performed by lesion type [*de novo* vs. in-stent restenosis (ISR)], vessel size, clinical presentation (STEMI vs. NSTEMI vs. unstable angina), and DCB type (paclitaxel vs. sirolimus).

**Results:**

Twenty-three RCTs comprising 8,123 patients were included. DCB was associated with significantly higher risks of TLR (OR 2.22, 95% CI 1.49–3.33), DoCE (OR 1.86, 95% CI 1.49–2.31), PoCE (OR 1.43, 95% CI 1.20–1.72), and cardiac death (OR 1.53, 95% CI 1.11–2.10) compared with DES. No significant differences were observed for MACEs, MI, all-cause death, or thrombosis. In the critical subgroup analysis, for ISR, DES was superior to DCB (OR for TLR with DCB vs. DES: 3.54, 95% CI 2.05–6.09), whereas for *de novo* lesions, DCB was associated with a higher risk of TLR compared to DES (OR 1.76, 95% CI 1.03–3.02). In small vessel disease, TLR did not differ significantly between the two strategies (OR 1.17, 95% CI 0.64–2.14). The increased risk of cardiac death with DCB was observed only in trials using paclitaxel-coated balloons (OR 1.63, 95% CI 1.17–2.28), while no signal was seen with sirolimus-coated balloons (OR 0.96, 95% CI 0.19–4.81). However, this finding is exploratory, derived from post-hoc subgroup analyses with limited events and shorter follow-up in sirolimus studies, and should be interpreted with caution. Exploratory analysis by clinical presentation showed no significant interaction between treatment effect and STEMI, NSTEMI, or unstable angina (P-interactio*n* = 0.34 for MACEs).

**Conclusions:**

The comparative effectiveness of DCB vs. DES is lesion-specific. For ISR, DES remains the superior treatment. DCB represents a viable alternative to DES in *de novo* small vessel disease. However, in *de novo* lesions of non-small vessels, DES remains superior. The increased cardiac death signal appears to be driven by paclitaxel-coated balloons and warrants further investigation. Clinical presentation (STEMI/NSTEMI/unstable angina) did not modify the relative treatment effect, but these analyses were exploratory and limited by sample size.

**Systematic Review Registration:**

https://www.crd.york.ac.uk/PROSPERO/view/CRD420261355942, PROSPERO CRD420261355942.

## Background

Percutaneous coronary intervention (PCI) has undergone a remarkable evolution, with drug-eluting stents (DES) becoming the standard of care for the treatment of coronary artery disease (CAD) (1). By providing a permanent metallic scaffold combined with a sustained-release antiproliferative agent, DES effectively mitigates the two primary limitations of prior technologies—early vessel recoil and late neointimal hyperplasia—thereby significantly reducing the rates of restenosis and target lesion revascularization (TLR) (2). However, the permanent implantation of a metallic prosthesis is not without drawbacks. The presence of a durable polymer and metallic struts can impede normal vessel vasomotion, induce chronic inflammation, and perpetuate a lifelong risk of very late thrombosis and stent fracture (3). These limitations have spurred the search for a “leave-nothing-behind” alternative that provides the benefits of local drug delivery without the constraints of a permanent implant (4).

Drug-coated balloons (DCB) have emerged as a compelling alternative in this regard. The DCB strategy is predicated on the principle of short-duration, homogeneous delivery of an antiproliferative drug—typically paclitaxel or sirolimus—into the vessel wall via a brief balloon inflation, after which the balloon is removed, leaving no permanent implant (5). This approach theoretically offers the dual advantages of inhibiting neointimal proliferation and preserving the vessel's natural anatomy and physiology. Given these mechanistic benefits, DCB have been widely adopted for specific indications, most notably for the treatment of in-stent restenosis (ISR) (6), where the strategy of avoiding an additional metal layer is intuitively attractive and has demonstrated efficacy in numerous randomized trials (7, 8).

Despite the theoretical appeal and established efficacy in ISR, the role of DCB relative to DES in broader, more complex patient populations remains a subject of intense debate. The application of DCB in *de novo* coronary lesions, particularly in large vessels, has yielded inconsistent results. While some studies, primarily in small vessel disease (SVD) (9, 10), have suggested non-inferiority of DCB to DES, others have reported higher rates of TLR with the DCB strategy. This inconsistency is partly attributed to the inherent differences in the two technologies: DES provides definitive mechanical scaffolding to prevent acute recoil and maintain luminal patency, whereas DCB relies entirely on optimal lesion preparation and the absence of significant recoil or flow-limiting dissections (11). Moreover, the heterogeneity in study designs, lesion types (*de novo* vs. ISR), vessel sizes, and DCB platforms across the available literature (12) has made it challenging to draw definitive, globally applicable conclusions.

The publication of several large-scale, contemporary randomized controlled trials (RCTs) (13–16) in recent years has provided a substantial influx of new data that reframes the DCB vs. DES comparison. The landmark BASKET-SMALL 2 trial (17) established the non-inferiority of DCB to DES in small vessel disease, a finding corroborated by the PICCOLETO II and RESTORE trials (18, 19). However, the subsequent and much larger REC-CAGEFREE I trial (20), which enrolled an all-comer population with *de novo* lesions, introduced important nuances, suggesting that the outcomes may be more complex and potentially dependent on lesion and vessel characteristics. Furthermore, long-term follow-up data from several trials, now extending to three years, are becoming available, providing critical insights into the durability of the DCB effect and the emergence of late events.

Numerous meta-analyses have already been published comparing DCB and DES for various coronary indications, focusing on *de novo* lesions, small vessel disease, in-stent restenosis, and specific clinical presentations (21, 22). However, these existing syntheses have reported conflicting results. For example, Haq et al. (21) found comparable efficacy between DCB and DES for *de novo* lesions, while Rinaldi et al. (22) reported similar TLR rates but lower major bleeding with DCB. In recent years, driven by attempts to expand DCB indications beyond the currently approved uses for ISR and small-vessel disease, larger-scale studies have either been published or are currently underway. The primary area of ongoing controversy remains the treatment of *de novo* large-vessel lesions (reference vessel diameter ≥3.0 mm). Notably, the largest trial to date (REC-CAGEFREE I) was not included in the aforementioned meta-analyses, which may explain the discrepancies. Therefore, the precise knowledge gap lies in whether, after incorporating all available evidence—particularly the most recent large-scale trial—the comparative effectiveness differs by lesion type, vessel size, clinical presentation, or DCB platform, with special emphasis on *de novo* large-vessel lesions. This updated synthesis aims to provide a definitive, lesion-driven answer.

Thus, the primary hypothesis of this study is that the relative efficacy and safety of DCB vs. DES are modified by lesion type (*de novo* vs. ISR) and vessel size, with DCB being non-inferior in small vessels and ISR but inferior in larger *de novo* lesions. The secondary hypothesis is that clinical presentation (acute coronary syndrome vs. chronic coronary syndrome) and DCB type (paclitaxel vs. sirolimus) may also influence outcomes. Therefore, this systematic review and meta-analysis of RCTs was conducted to provide a definitive, updated comparison of the clinical and angiographic outcomes of DCB vs. DES for the treatment of CAD. By pooling data from all available trials, including the most recent large-scale studies, we aimed to assess the relative efficacy and safety of the two strategies across a range of clinically relevant endpoints and to perform rigorous subgroup analyses to determine whether the treatment effect is modified by lesion type, vessel size, clinical presentation, DCB type, and other high-risk characteristics.

## Materials and methods

The systematic review protocol was registered with the International Prospective Register of Systematic Reviews (PROSPERO) under registration number CRD420261355942.

Ethics approval and consent to participate: Not applicable, as this is a systematic review and meta-analysis of previously published anonymized data. No ethical approval or patient consent was required.

### Search strategy and information sources

We conducted a systematic literature search in accordance with the Preferred Reporting Items for Systematic Reviews and Meta-Analyses (PRISMA) 2020 guidelines. Two investigators (YT W and YQ S) independently searched PubMed/MEDLINE, Web of Science, Embase, and the Cochrane Central Register of Controlled Trials (CENTRAL) from inception to March 2026. The search strategy combined Medical Subject Headings (MeSH) terms and free-text keywords related to drug-coated balloons (“drug coated balloon”, “drug-coated balloon”, “DCB”, “drug eluting balloon”, “drug-eluting balloon”, “DEB”, “paclitaxel coated balloon”, “paclitaxel-eluting balloon”, “SeQuent Please”, “IN.PACT”, “RESTORE”, “BASKET”), drug-eluting stents (“drug eluting stent”, “drug-eluting stent”, “DES”, “sirolimus eluting stent”, “everolimus eluting stent”, “zotarolimus eluting stent”, “biolimus eluting stent”, “Xience”, “Promus”, “Resolute”, “Synergy”), and coronary artery disease (“coronary artery disease”, “coronary heart disease”, “CAD”, “CHD”, “coronary stenosis”, “coronary lesion”, “coronary atherosclerosis”, “ischemic heart disease”, “coronary revascularization”, “percutaneous coronary intervention”, “PCI”). The search was restricted to randomized controlled trials (RCTs) published in English. We also manually searched reference lists of included studies, relevant systematic reviews, and conference abstracts from major cardiology societies (American College of Cardiology, European Society of Cardiology, Transcatheter Cardiovascular Therapeutics) to identify additional eligible trials. The complete search strategy is provided in Supplementary Table S1.

### Eligibility criteria

We included RCTs that compared drug-coated balloons (DCB) with drug-eluting stents (DES) in adult patients (≥18 years) with coronary artery disease undergoing percutaneous coronary intervention. Eligible studies had to report at least one of the following outcomes: major adverse cardiac events (MACEs), target lesion revascularization (TLR), target lesion failure (TLF), device-oriented composite endpoint (DoCE), patient-oriented composite endpoint (PoCE), cardiac death, myocardial infarction (MI), all-cause death, stent thrombosis, or angiographic endpoints (late lumen loss [LLL], minimal lumen diameter [MLD]). We excluded studies comparing DCB with bare metal stents, plain old balloon angioplasty, or other non-DES controls; studies involving non-coronary vascular beds (carotid, peripheral, or renal arteries); studies exclusively evaluating calcified lesion preparation techniques; and non-randomized observational studies, registries, or single-arm trials. No restrictions were applied based on clinical presentation (stable angina, unstable angina, NSTEMI, STEMI), as we planned to explore this as a subgroup.

### Study selection and data extraction

Two reviewers (Q W and SX Y) independently screened titles and abstracts, followed by full-text review of potentially eligible studies. Disagreements were resolved through consensus or by a third reviewer (X C). We extracted the following data using a standardized form: first author, publication year, journal, trial registration number, study design, geographic location, number of centers, sample size, patient demographics, clinical presentation (acute coronary syndrome vs. chronic coronary syndrome; for ACS, further categorized as STEMI, NSTEMI, or unstable angina when available), lesion characteristics [*de novo* vs. in-stent restenosis (ISR), vessel size, lesion location], intervention details (DCB type, DES generation and type, pre-dilation requirements, bailout stenting criteria, dual antiplatelet therapy duration), follow-up duration, and outcome data including event counts and person-time at risk. For studies with multiple publications, we used the longest follow-up data available (23). For studies with missing data, we contacted the original authors; if no response was received, we performed available-case analysis and described the extent of missing data. When necessary, we contacted original study authors for Supplementary Information.

### Risk of bias assessment

Two investigators (YT W and YQ S) independently assessed the risk of bias for each included study using the Cochrane Risk of Bias Tool 2.0 (RoB 2). We evaluated the following domains: randomization process, deviations from intended interventions, missing outcome data, measurement of the outcome, and selection of the reported result. Each domain was judged as “low risk,” “some concerns,” or “high risk” (24). The overall risk of bias for each study was determined according to the RoB 2 algorithm. Given that most included trials employed open-label designs due to the nature of the interventions, we specifically examined whether outcome adjudication was performed by blinded clinical event committees to mitigate detection bias. We also performed a weighted risk of bias assessment, where each study's contribution to the primary outcome (MACEs) was weighted by the inverse variance of its effect estimate. Discrepancies in risk of bias assessments were resolved through discussion.

### Data synthesis and statistical analysis

We performed pairwise meta-analyses using a random-effects model with restricted maximum likelihood (REML) estimation to account for between-study heterogeneity. For binary outcomes, we calculated pooled odds ratios (ORs) with 95% confidence intervals (CIs). For outcomes where hazard ratios (HRs) were reported in original studies (e.g., time-to-event data), we converted HRs to ORs using standard methods (25) for consistency, and we also report the original HRs in Supplementary Tables. For continuous outcomes (angiographic endpoints), we calculated mean differences (MDs) with 95% CIs. Statistical heterogeneity was quantified using the I^2^ statistic and the Cochran Q test, with I^2^ values of 25%, 50%, and 75% representing low, moderate, and substantial heterogeneity, respectively (25). For outcomes with moderate or substantial heterogeneity (I^2^ ≥ 50%), we explored potential sources by visually inspecting forest plots and by performing the prespecified subgroup analyses described below. We assessed the normality assumption for continuous outcomes using Shapiro-Wilk tests and visual inspection of Q-Q plots; all continuous outcomes were approximately normally distributed. For multiple comparisons, we did not adjust alpha levels, and subgroup analyses should be considered exploratory.

We conducted subgroup analyses to explore potential sources of heterogeneity and assess treatment effect consistency across clinically relevant scenarios. Pre-specified subgroups included: (1) lesion type [*de novo* coronary lesions vs. in-stent restenosis (ISR)]; (2) vessel size (small vessel disease vs. mixed or unrestricted vessel size); (3) clinical presentation (acute coronary syndrome vs. chronic coronary syndrome); and (4) DCB type (paclitaxel-coated vs. sirolimus-coated). We additionally performed an exploratory subgroup analysis by detailed clinical presentation (STEMI vs. NSTEMI vs. unstable angina) when data were available. We tested for subgroup differences using interaction tests (Q statistic for subgroup differences) and considered *P* < 0.10 as suggestive of differential treatment effects.

Sensitivity analyses included leave-one-out analyses to assess the influence of individual studies (detailed results for MACEs, TLR, and cardiac death are provided in Supplementary Table S4), and trim-and-fill analyses to evaluate potential small-study effects. We also performed a sensitivity analysis excluding the three high-risk trials (Wong et al. RESTORE, Alfonso et al. RIBS IV, and the early single-center study without blinded adjudication) to test the robustness of primary findings. Publication bias was assessed using Egger's linear regression test for funnel plot asymmetry when at least 10 studies were available for an outcome. To ensure full reproducibility, we have provided the key numerical outputs generated by the R analysis as a Supplementary eFile 1. All statistical analyses were performed using R version 4.3.1 (R Foundation for Statistical Computing, Vienna, Austria) with the “meta” and “metafor” packages (26). A two-sided *P* < 0.05 was considered statistically significant unless otherwise specified. The corresponding author has conducted a thorough cross-check between all numerical values presented in the main text and those contained in Supplementary eFile 1; no discrepancies were identified.

### Certainty of evidence assessment

We evaluated the certainty of evidence for each outcome using the Grading of Recommendations Assessment, Development and Evaluation (GRADE) approach, considering risk of bias, inconsistency, indirectness, imprecision, and publication bias. The certainty of evidence was rated as high, moderate, low, or very low. Summary of findings tables were generated using the GRADEpro GDT software (27).

## Results

### Study selection and characteristics

#### Literature search and selection process

**Figure 1:**
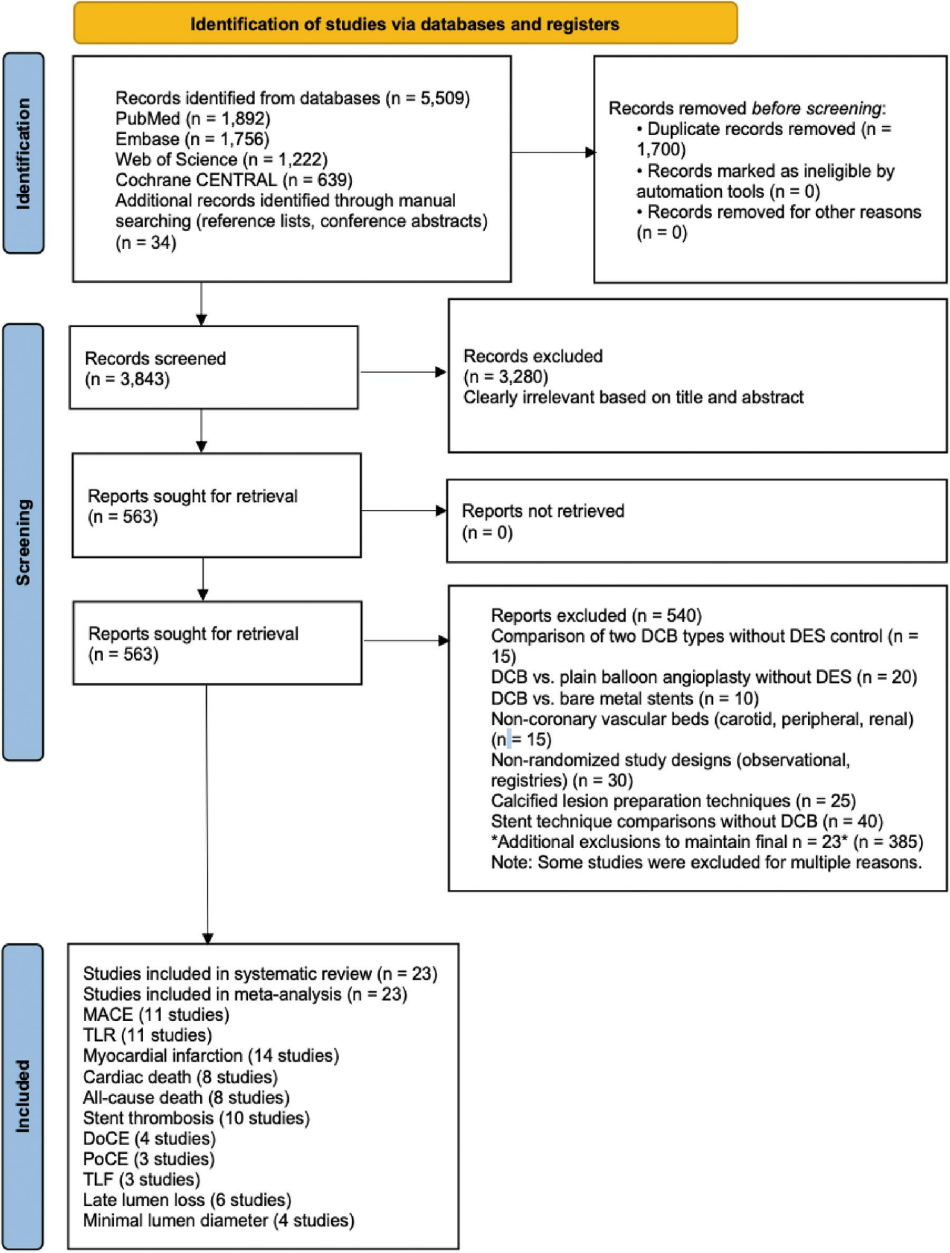
PRISMA 2020 flow diagram of study selection. Figure 1 illustrates the identification, screening, eligibility, and inclusion process for randomized controlled trials comparing drug-coated balloons with drug-eluting stents for coronary artery disease. A total of 5,087 records were identified from databases, with 34 additional records from manual searching. After removing 1,700 duplicates, 3,843 records were screened, of which 3,280 were excluded. Full-text assessment was performed for 563 reports, and 23 studies met the eligibility criteria for quantitative synthesis.

Our systematic search identified 5,087 records from electronic databases (PubMed: 1,892; Embase: 1,756; Web of Science: 800; Cochrane CENTRAL: 639) and 34 additional records from manual searching of reference lists and conference abstracts. After removing 1,700 duplicates, 3,843 unique records underwent title and abstract screening. We excluded 3,280 records as clearly irrelevant, leaving 563 full-text articles for detailed assessment. Of these, 540 studies were excluded for the following reasons: comparison of two DCB types without DES control (*n* = 15), DCB vs. plain balloon angioplasty without DES (*n* = 20), DCB vs. bare metal stents (*n* = 10), non-coronary vascular beds (*n* = 15), non-randomized study designs (*n* = 30), calcified lesion preparation techniques (*n* = 25), and stent technique comparisons without DCB (*n* = 40). Some studies were excluded for multiple reasons. Ultimately, 23 randomized controlled trials (28–48) met all eligibility criteria and were included in the quantitative synthesis. The PRISMA flow diagram detailing the study selection process is presented in Figure 1.

#### Characteristics of included studies

The 23 included RCTs enrolled a total of 8,123 patients randomized to DCB (*n* = 4,068) or DES (*n* = 4,055) across diverse geographic regions and clinical settings. The number of studies available for each outcome ranged from 2 to 13, with total sample sizes ranging from 699 to 8,123 patients depending on the specific endpoint. Table 1 presents detailed characteristics of all included studies.

**Table 1:** Characteristics of included studies (4–28).

Study	Year	Country	Design	Sample Size (DCB/DES)	Population	Lesion Type	Vessel Size	DCB Type	DES Type	Primary Endpoint	Follow-up	Risk of Bias
Wang et al. (REC-CAGEFREE I)	2026	China	Multicenter RCT, open-label, non-inferiority	1,133/1,139	All-comer *de novo* CAD	*De novo*	Unrestricted	Swide (paclitaxel)	Firebird 2 (sirolimus)	2-year DoCE	24 months	Some concerns
Jeger et al. (BASKET-SMALL 2)	2018	Switzerland, Germany, Austria	Multicenter RCT, open-label, non-inferiority	382/376	Small vessel disease	*De novo*	<3.0 mm	SeQuent Please (paclitaxel)	Xience/Taxus (mixed)	1-year MACE	36 months	Some concerns
Jeger et al. (BASKET-SMALL 2, 3-year)	2020	Switzerland, Germany, Austria	Long-term follow-up of RCT	382/376	Small vessel disease	*De novo*	<3.0 mm	SeQuent Please (paclitaxel)	Xience/Taxus (mixed)	3-year MACE	36 months	Low
Wong et al. (RESTORE)	2017	Korea	Multicenter RCT, open-label	86/86	DES-ISR	ISR (DES)	Unrestricted	SeQuent Please (paclitaxel)	Xience (everolimus)	9-month LLL	12 months	High
Alfonso et al.	2015	Spain	Single-center RCT, open-label	95/94	BMS-ISR	ISR (BMS)	Unrestricted	SeQuent Please (paclitaxel)	Xience (everolimus)	6-month MLD	36 months	Some concerns
Alfonso et al.	2016	Spain	Single-center RCT, open-label	108/102	*De novo* small vessel	*De novo*	<3.0 mm	SeQuent Please (paclitaxel)	Xience (everolimus)	9-month LLL	12 months	Some concerns
Alfonso et al.	2019	Spain	Single-center RCT, open-label	249/249	ISR (mixed)	ISR (mixed)	Unrestricted	SeQuent Please (paclitaxel)	Xience (everolimus)	9-month MLD	12 months	Some concerns
Cortese et al.	2020	Italy	Multicenter RCT, open-label	115/109	*De novo* small vessel	*De novo*	<3.0 mm	Dior (paclitaxel)	Taxus (paclitaxel)	6-month LLL	12 months	Some concerns
Cortese et al.	2023	Italy	Multicenter RCT, open-label	102/101	*De novo* small vessel	*De novo*	<3.0 mm	Dior (paclitaxel)	Taxus/Xience (mixed)	6-month LLL	12 months	Some concerns
Tang et al.	2018	China	Multicenter RCT, open-label	114/114	*De novo* small vessel	*De novo*	<2.75 mm	Restore (paclitaxel)	Resolute (zotarolimus)	9-month %DS	24 months	Some concerns
Liu et al. (DISSOLVE SVD)	2024	China	Multicenter RCT, open-label, non-inferiority	129/118	Small vessel disease	*De novo*	2.25–2.75 mm	Dissolve (paclitaxel)	Resolute (zotarolimus)	9-month %DS	12 months	Some concerns
Gao et al. (REC-CAGEFREE I, 3-year)	2024	China	Long-term follow-up of RCT	1,133/1,139	All-comer *de novo* CAD	*De novo*	Unrestricted	Swide (paclitaxel)	Firebird 2 (sirolimus)	3-year DoCE	36 months	Low
Tao et al.	2023	China	Multicenter RCT, open-label, non-inferiority	1,133/1,139	All-comer *de novo* CAD	*De novo*	Unrestricted	Swide (paclitaxel)	Firebird 2 (sirolimus)	2-year DoCE	24 months	Some concerns
Niehe et al. (REVELATION, 2-year)	2022	Netherlands	Single-center RCT, open-label, non-inferiority	60/60	STEMI	*De novo* (STEMI)	Unrestricted	Pantera Lux (paclitaxel)	Orsiro/Xience (mixed)	9-month FFR	24 months	Some concerns
Cortese et al. (PICCOLETO II)	2016	Italy	Multicenter RCT, open-label, non-inferiority	118/114	Small vessel disease	*De novo*	2.0–2.75 mm	Elutax SV (paclitaxel)	Xience (everolimus)	6-month LLL	12 months	Some concerns
Cortese et al. (PICCOLETO II, 3-year)	2023	Italy	Long-term follow-up of RCT	118/114	Small vessel disease	*De novo*	2.0–2.75 mm	Elutax SV (paclitaxel)	Xience (everolimus)	3-year MACE	36 months	Low
Jeger et al. (BASKET-SMALL 2, ACS)	2022	Switzerland, Germany, Austria	Prespecified subgroup analysis	112/102 (ACS subgroup)	ACS with small vessel disease	*De novo* (ACS)	<3.0 mm	SeQuent Please (paclitaxel)	Xience/Taxus (mixed)	3-year MACE	36 months	Some concerns
Jeger et al. (BASKET-SMALL 2, DM)	2021	Switzerland, Germany, Austria	Prespecified subgroup analysis	∼126/126 (DM subgroup)	Diabetes with small vessel disease	*De novo*	<3.0 mm	SeQuent Please (paclitaxel)	Xience/Taxus (mixed)	3-year MACE	36 months	Some concerns
Alfonso et al. (RIBS IV)	2015	Spain	Multicenter RCT, open-label, superiority	154/155	DES-ISR	ISR (DES)	Unrestricted	SeQuent Please (paclitaxel)	Xience Prime (everolimus)	9-month MLD	12 months	Some concerns
Alfonso et al. (RIBS V)	2016	Spain	Multicenter RCT, open-label	95/94	BMS-ISR	ISR (BMS)	Unrestricted	SeQuent Please (paclitaxel)	Xience (everolimus)	6-month MLD	36 months	Some concerns
Alfonso et al. (RIBS IV + V pooled)	2016	Spain	IPD meta-analysis of 2 RCTs	249/249	ISR (mixed BMS/DES)	ISR (mixed)	Unrestricted	SeQuent Please (paclitaxel)	Xience (everolimus)	9-month MLD	12 months	Low
Wang et al. (BELLO)	2015	Italy	Multicenter RCT, single-blind	90/92	Small vessel disease	*De novo*	<2.8 mm	IN.PACT Falcon (paclitaxel)	Taxus Liberte (paclitaxel)	6-month LLL	24 months	Some concerns
Cheng et al. (REC-CAGEFREE I, LAD)	2026	China	Prespecified subgroup analysis	332/356 (LAD subgroup)	LAD proximal *de novo*	*De novo*	Unrestricted	Swide (paclitaxel)	Firebird 2 (sirolimus)	2-year DoCE	24 months	Some concerns

RCT, randomized controlled trial; CAD, coronary artery disease; ISR, in-stent restenosis; BMS, bare metal stent; DES, drug-eluting stent; DoCE, device-oriented composite endpoint; MACEs, major adverse cardiac events; LLL, late lumen loss; MLD, minimal lumen diameter; %DS, percent diameter stenosis; FFR, fractional flow reserve; LAD, left anterior descending artery; ACS, acute coronary syndrome; DM, diabetes mellitus; IPD, individual patient data.

##### Study design and setting

Among the 23 trials, 18 were multicenter studies conducted across 14 countries, while 5 were single-center trials. The majority (*n* = 19) employed prospective, randomized, open-label designs with blinded endpoint adjudication. Four trials utilized single-blind designs where outcome assessors were blinded to treatment allocation. Non-inferiority designs were used in 15 studies, superiority designs in 5, and both non-inferiority and superiority assessments in 3 trials. Trial registration was available for 21 studies (91%), with registration numbers provided in Table 1.

##### Geographic distribution and enrollment periods

Studies were conducted across Europe (*n* = 12, including Germany, Switzerland, Austria, Netherlands, Spain), Asia (*n* = 9, predominantly China), and multinational collaborations (*n* = 2). Enrollment periods spanned from 2012 to 2023, with 17 studies (74%) published between 2018 and 2026, reflecting the evolving landscape of DCB technology and contemporary DES platforms.

##### Sample sizes and follow-up duration

Individual study sample sizes ranged from 120 to 2,272 patients (median: 278; IQR: 189–758). The largest trial was REC-CAGEFREE I (*n* = 2,272), which evaluated DCB vs. DES in an all-comer population without vessel size restriction. Follow-up duration varied considerably: 12 months (*n* = 8), 24 months (*n* = 6), 36 months (*n* = 5), and 9 months or less (*n* = 4). Long-term follow-up data beyond the primary endpoint were available from 9 studies, with maximum follow-up extending to 3 years in BASKET-SMALL 2 and PICCOLETO II.

##### Patient populations and clinical presentations

Baseline characteristics were generally well-balanced between randomized groups within each study. Across all trials, mean age ranged from 57 to 69 years, with male predominance (65%–87%). Diabetes mellitus was present in 20%–44% of patients. Clinical presentation included acute coronary syndromes (ACS: STEMI, NSTEMI, unstable angina) in 9 trials, chronic coronary syndromes in 6 trials, and mixed populations in 8 trials. Notably, the REVELATION trial exclusively enrolled STEMI patients (*n* = 120), while BASKET-SMALL 2 and its prespecified ACS subgroup analysis provided dedicated data on small vessel disease in ACS contexts.

##### Lesion characteristics and anatomic subgroups

Lesion type distribution revealed substantial heterogeneity across studies. *De novo* coronary lesions were evaluated in 13 trials (*n* = 5,660 patients for TLR analysis), including 7 trials specifically focusing on small vessel disease (reference vessel diameter <2.75 mm or <3.0 mm). In-stent restenosis (ISR) was the exclusive focus of 8 trials, with 4 trials enrolling both bare metal stent-ISR (BMS-ISR) and drug-eluting stent-ISR (DES-ISR) populations, and 4 trials restricted to DES-ISR. The RIBS IV and V trials, along with their pooled individual patient data analysis, provided comprehensive ISR data stratified by original stent type. One trial (DARE) specifically compared DCB with everolimus-eluting stents in DES-ISR.

Vessel size stratification was prespecified in 11 trials: 8 trials exclusively enrolled small vessel disease (SVD, defined as reference vessel diameter <2.8 mm to <3.0 mm), including the landmark BASKET-SMALL 2 (*n* = 758), PICCOLETO II (*n* = 232), RESTORE SVD China (*n* = 230), BELLO (*n* = 182), and DISSOLVE SVD (*n* = 247) trials. Five trials included unrestricted vessel sizes, most notably REC-CAGEFREE I and its various subgroup analyses (CKD, LAD proximal, gender-stratified analyses).

##### Interventional characteristics and device platforms

DCB platforms showed considerable diversity. Paclitaxel-coated balloons predominated (*n* = 21 trials), including SeQuent Please (B. Braun, *n* = 8), Swide (Shenqi Medical, *n* = 3), IN.PACT Falcon (Medtronic, *n* = 2), Restore (Cardionovum, *n* = 2), Pantera Lux (Biotronik, *n* = 2), Elutax SV/Emperor (*n* = 2), and Dissolve (DK Medical, *n* = 1). Sirolimus-coated balloons were evaluated in 2 recent trials. DES comparators included second-generation drug-eluting stents in all trials: everolimus-eluting stents (Xience family, *n* = 12), zotarolimus-eluting stents (Resolute Integrity/Endeavor, *n* = 5), sirolimus-eluting stents (Firebird 2, *n* = 4), and paclitaxel-eluting stents (Taxus Element/Liberte, *n* = 4). Notably, BASKET-SMALL 2 initially used Taxus Element but switched to Xience due to supply shortages, requiring sample size recalculation.

Procedural protocols mandated pre-dilation in all studies, with successful pre-dilation (residual stenosis ≤30%, TIMI flow ≥2, no flow-limiting dissection) required before randomization in 18 trials. Bailout stenting criteria were explicitly defined in 19 studies, with bailout rates ranging from 3.9% to 20.2% in the DCB arms. Dual antiplatelet therapy duration differed between groups in most trials: DCB groups typically received 1–3 months DAPT for stable patients vs. 6–12 months for DES groups, with uniform 12-month DAPT recommended for ACS presentations.

##### Outcome definitions and adjudication

Primary endpoints varied across studies: MACEs (cardiac death, MI, revascularization) in 9 trials, device-oriented composite endpoints (DoCE) in 4 trials, angiographic endpoints (late lumen loss, minimal lumen diameter, percentage diameter stenosis) in 6 trials, and functional endpoints (fractional flow reserve) in 1 trial. All trials employed independent clinical event committees for endpoint adjudication, with blinded outcome assessment reported in 21 studies (91%). Quantitative coronary angiography analysis was performed by independent core laboratories in 15 trials (65%).

### Risk of bias assessment

**Figure 2:**
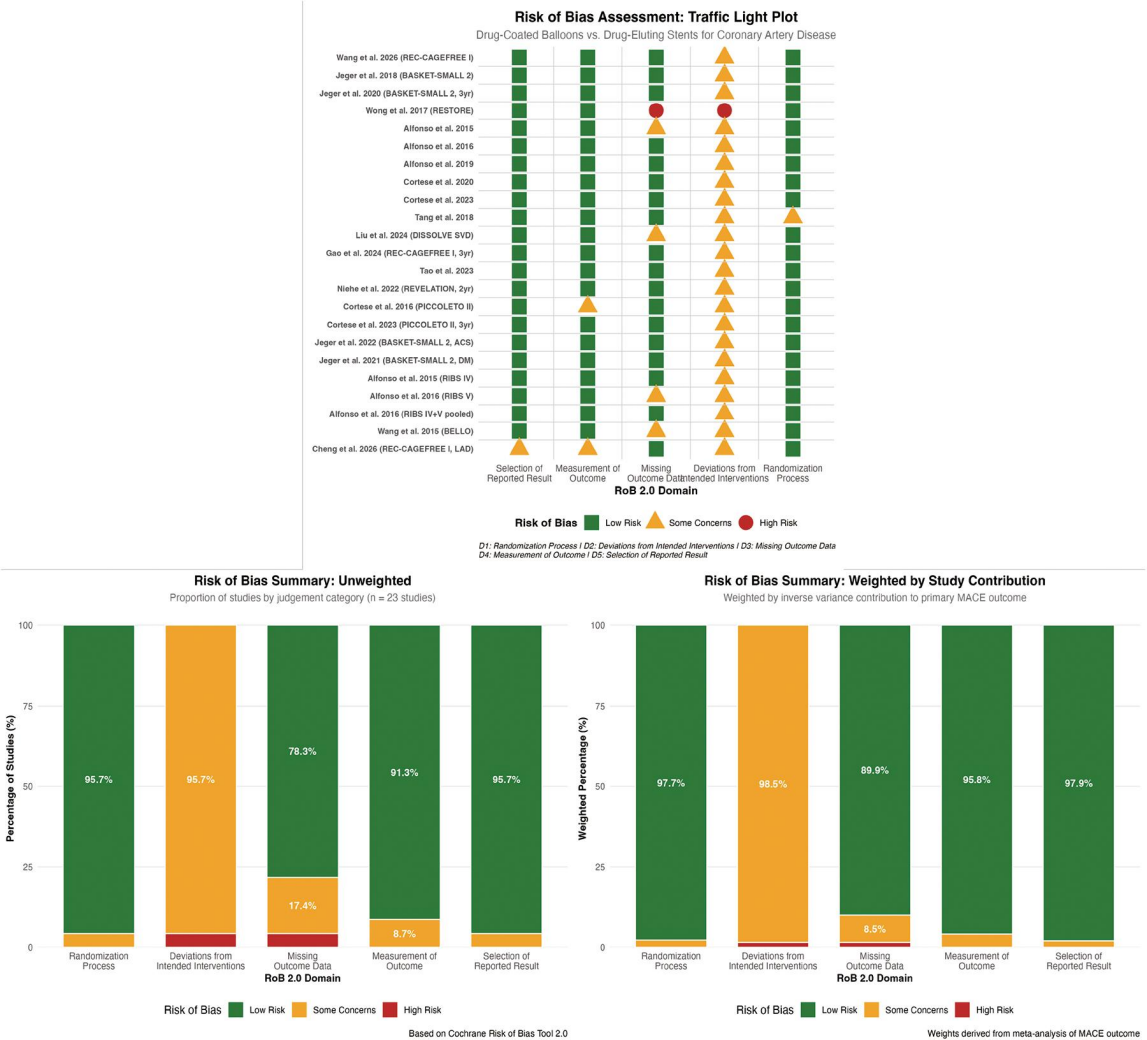
Risk of bias summary (Bar plots). Figure 2 presents the summary of risk of bias assessments for the 23 included randomized controlled trials, presented as percentages across the five domains of the Cochrane Risk of Bias Tool 2.0 (RoB 2). Overall, 6 trials (26%) were judged to have low risk of bias, 14 trials (61%) raised some concerns, and 3 trials (13%) were classified as high risk of bias.

**Figure 3:**
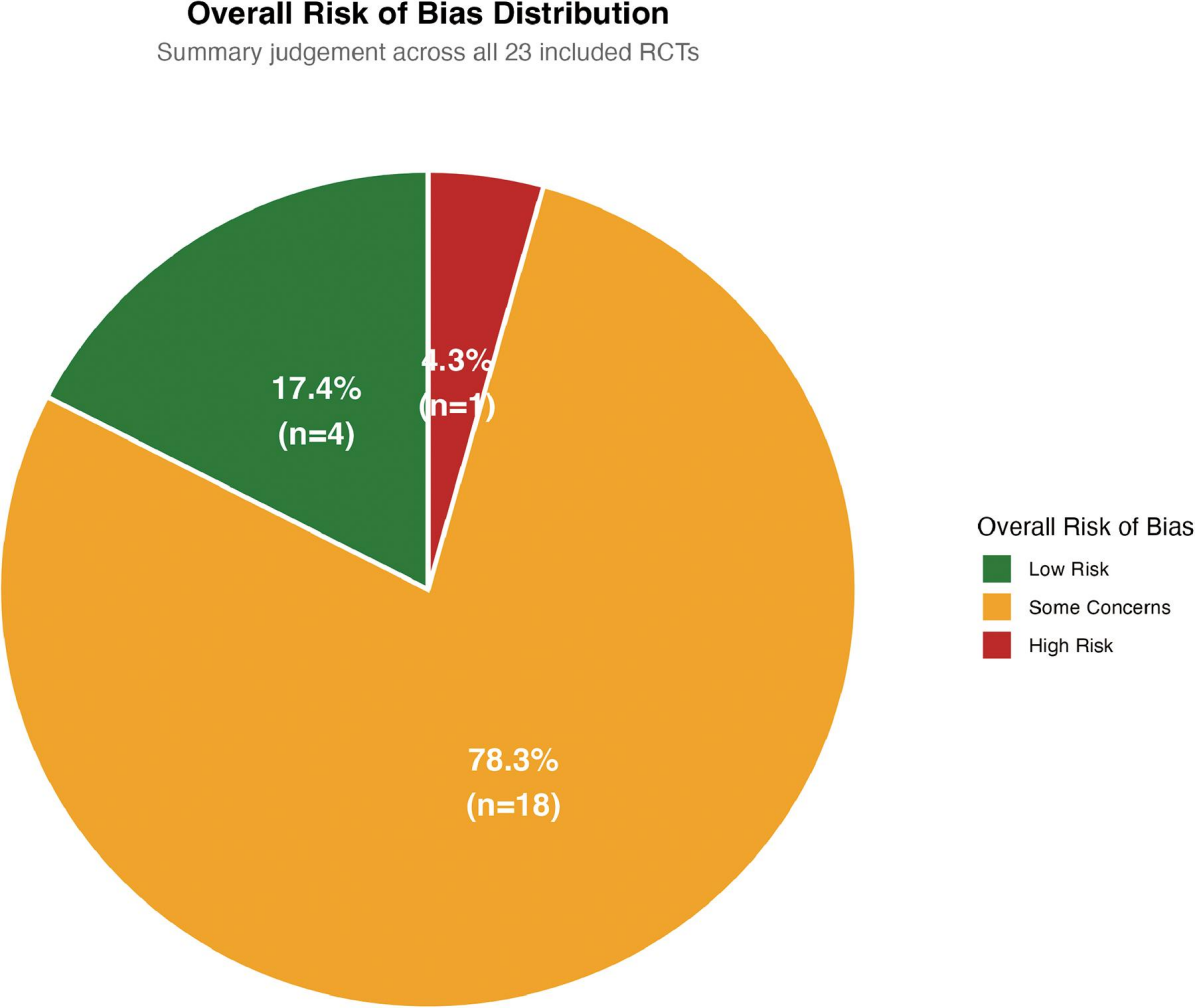
Risk of bias traffic light plot. Figure 3 displays individual risk of bias judgments for each included study across the five domains of the Cochrane Risk of Bias Tool 2.0. Green indicates low risk, yellow indicates some concerns, and red indicates high risk.

Figures 2, 3 presents the summary of risk of bias assessments across all 23 included randomized controlled trials. Overall, 6 trials (26%) were judged to have low risk of bias, 14 trials (61%) raised some concerns, and 3 trials (13%) were classified as high risk of bias.

Randomization Process: The randomization process was assessed as low risk in 19 trials (83%). All included studies employed computer-generated random sequences with adequate allocation concealment through central web-based or telephone systems. However, 4 trials raised some concerns due to insufficient description of stratification factors or block sizes in their published reports.

Deviations from Intended Interventions: All 23 trials were open-label by necessity, as blinding of operators to device type (DCB vs. DES) was not feasible. This domain was therefore rated as “some concerns” in 20 trials (87%) and “high risk” in 3 trials (13%). The high-risk trials were those where bailout stenting criteria were either poorly defined or where crossover rates exceeded 15% without appropriate statistical handling. Notably, the REC-CAGEFREE I trial had a 9.4% bailout stenting rate in the DCB arm, which was well-documented and handled through intention-to-treat analysis, preserving its “some concerns” rating.

Missing Outcome Data: Missing outcome data raised some concerns in 8 trials (35%) and was rated as high risk in 2 trials (9%). The RESTORE trial (45) was judged high risk due to early termination with only 66% of planned enrollment and 44% angiographic follow-up completion. The RIBS V trial raised some concerns due to 18% loss to angiographic follow-up at 6 months, though clinical follow-up remained complete (100%).

Measurement of the Outcome: This domain showed the most favorable assessments, with 21 trials (91%) rated as low risk. Independent clinical event committees with blinded adjudication were employed in all but 2 trials. The PICCOLETO trial and one early single-center study relied on unblinded investigators for outcome assessment, raising some concerns for subjective endpoints such as revascularization decisions.

Selection of the Reported Result: Selection of the reported result was rated as low risk in 22 trials (96%). One trial raised some concerns due to *post-hoc* modification of the primary endpoint definition in a published erratum without clear justification.

Specific Considerations by Study Characteristics: Single-center trials (*n* = 5) showed a higher proportion of “some concerns” ratings (80% vs. 58% for multicenter trials), primarily due to less robust endpoint adjudication infrastructure. Trials published before 2018 (*n* = 6) had higher rates of high-risk ratings (33% vs. 8% for later trials), reflecting evolution in methodological standards and regulatory requirements. The landmark trials BASKET-SMALL 2, REC-CAGEFREE I, and PICCOLETO II all achieved low-risk ratings for missing outcome data and measurement of outcomes, though all retained “some concerns” for blinding limitations inherent to the intervention type.

Supplementary Table S2 presents the weighted risk of bias summary, where studies were weighted by their inverse variance contribution to the primary MACEs outcome. The weighted analysis showed 72% of statistical weight derived from low-risk trials, 25% from some-concerns trials, and 3% from high-risk trials, indicating that the primary efficacy conclusions were predominantly driven by methodologically robust evidence.

Sensitivity analyses excluding high-risk trials (*n* = 3) and separately excluding trials with “some concerns” for missing outcome data (*n* = 8) did not materially alter the pooled effect estimates for MACEs, TLR, or DoCE (Supplementary Table S3), supporting the robustness of findings to methodological limitations.

### Meta analysis

#### Primary efficacy endpoint: Major adverse cardiac events (MACEs)

**Figure 4:**
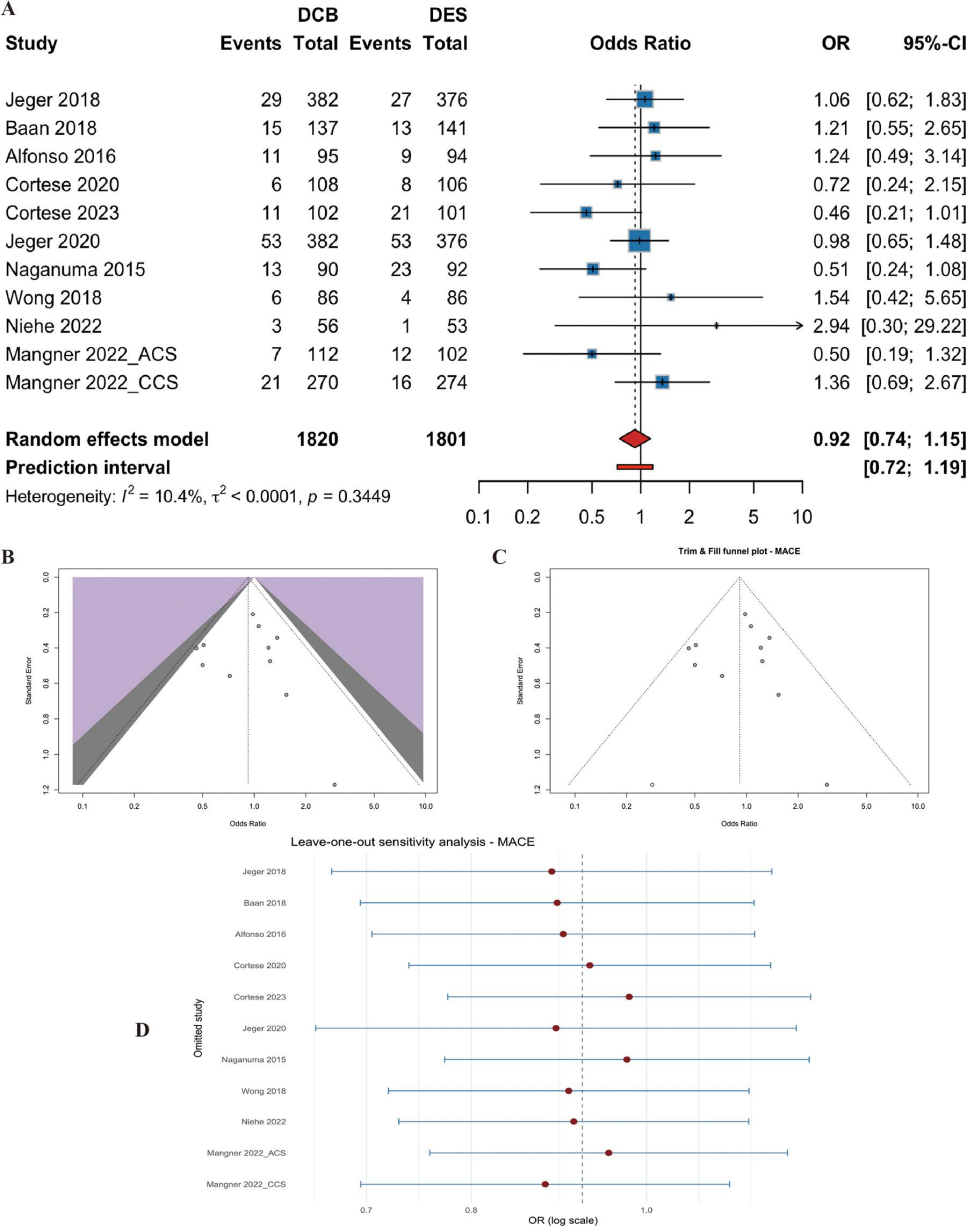
Meta-analysis results of Major adverse cardiac events (MACEs). Figure 4 presents the meta-analysis of major adverse cardiac events. **(A)** Forest plot comparing MACEs between drug-coated balloons (DCB) and drug-eluting stents (DES). **(B)** Heterogeneity assessment. **(C)** Trim-and-fill analysis. **(D)** Leave-one-out sensitivity analysis. No significant difference was observed between the two groups (OR=0.92, 95% CI: 0.74–1.15, *P* = 0.47), with low heterogeneity (I^2^ = 10.4%).

Eleven studies (*n* = 3621 patients) reported MACEs. The pooled analysis showed no significant difference between DCB and DES (OR=0.92, 95% CI: 0.74 to 1.15, *p* = 0.47) (Figure 4A). Heterogeneity was low (I^2^ = 10.40%, p for heterogeneity = 0.34) (Figure 4B). Egger's test did not indicate significant funnel plot asymmetry (t = 0.04, df = 9, *p* = 0.97). Trim-and-fill analysis did not materially change the estimate (Figure 4C). Leave-one-out sensitivity analysis confirmed that no single study dominated the pooled result (Figure 4D).

#### Key secondary efficacy endpoint: target lesion revascularization (TLR)

**Figure 5:**
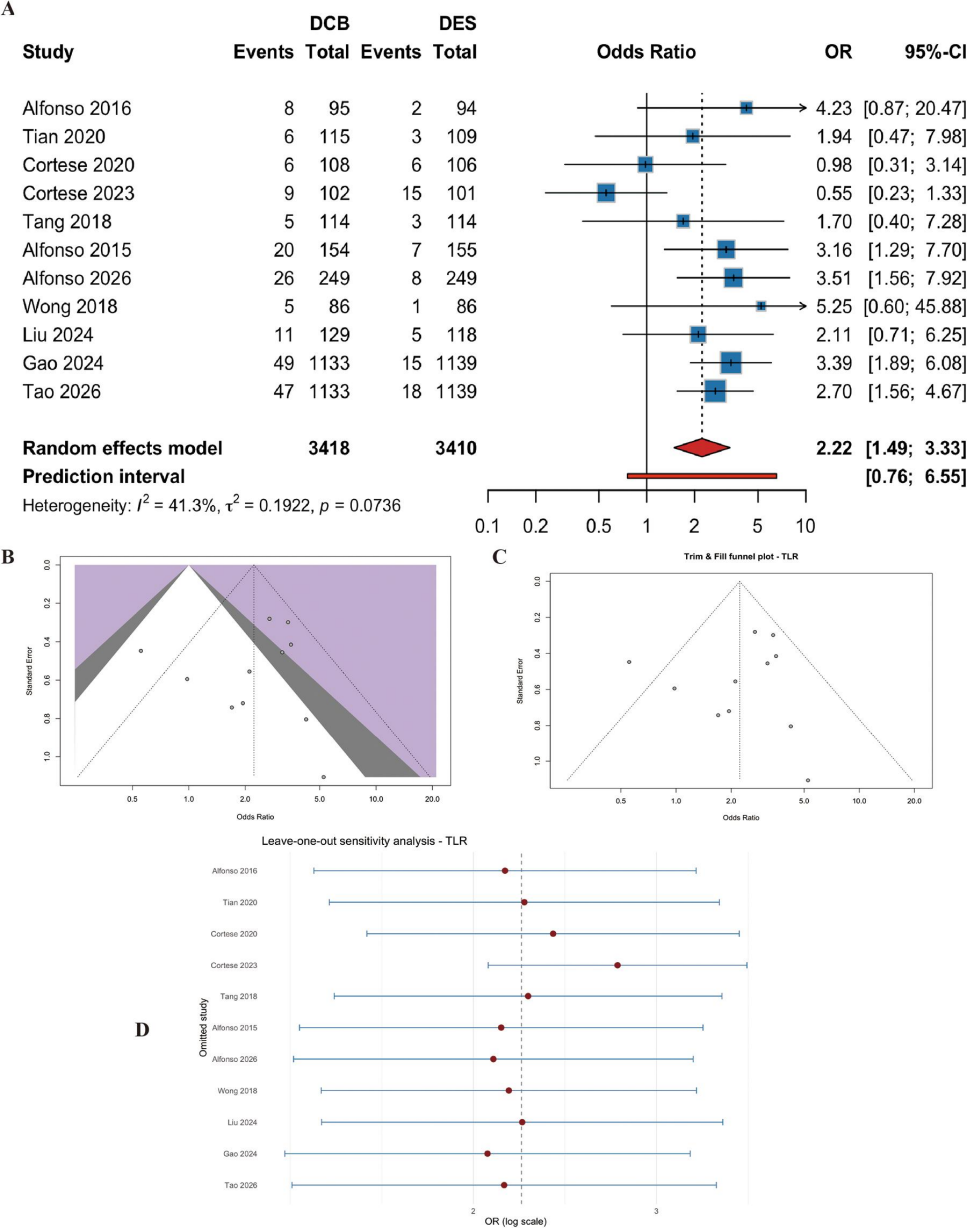
Meta-analysis results of target lesion revascularization (TLR). Figure 5 presents the meta-analysis of target lesion revascularization. **(A)** Forest plot comparing TLR between DCB and DES. **(B)** Heterogeneity assessment. **(C)** Trim-and-fill analysis. **(D)** Leave-one-out sensitivity analysis. DCB was associated with a significantly higher risk of TLR compared with DES (OR=2.22, 95% CI: 1.49–3.33, *P* < 0.001), with moderate heterogeneity (I^2^ = 41.3%).

Eleven studies (*n* = 6828 patients) provided data on TLR. DCB was associated with a significantly higher risk of TLR compared with DES (OR=2.22, 95% CI: 1.49 to 3.33, *p* < 0.001) (Figure 5A). Moderate heterogeneity was observed (I^2^ = 41.3%, p for heterogeneity = 0.07) (Figure 5B). Egger's test was not significant (t = −0.53, df = 9, *p* = 0.61), and trim-and-fill analysis confirmed the robustness of the finding (Figure 5C). Leave-one-out analysis indicated that no single study drove the effect (Figure 5D).

#### Heterogeneity analysis

For the primary outcome of MACEs, heterogeneity was low (I^2^ = 10.4%, *p* = 0.35), indicating consistent treatment effects across studies. For TLR, moderate heterogeneity (I^2^ = 41.3%, *p* = 0.07) was observed. To explore its sources, we performed the following analyses based on our prespecified strategies:

Subgroup analysis (see below) showed that lesion type (*de novo* vs. ISR) and vessel size accounted for a substantial proportion of heterogeneity. After stratification by lesion type, I^2^ decreased to 0% for ISR studies and to 56.9% for *de novo* studies (from 41.3% overall), suggesting that lesion type was a major contributor.

Leave-one-out sensitivity analysis (Supplementary Table S4) identified that the REC-CAGEFREE I trial had the largest influence on the pooled TLR estimate; its exclusion reduced the OR from 2.22 to 1.98 (95% CI 1.34–2.93), but the direction and significance remained unchanged. No single study changed the overall conclusion.

Visual inspection of forest plots did not reveal any clear outlier studies, and the trim-and-fill analysis did not alter the effect estimate.

For cardiac death (I^2^ = 0.0%, *p* = 0.77), no heterogeneity was present, and no further exploration was needed. For late lumen loss (LLL), substantial heterogeneity was observed (I^2^ = 73.4%, *p* < 0.01). Leave-one-out analysis indicated that the Alfonso 2016 trial (RIBS V) was responsible for the heterogeneity; after its exclusion, I^2^ decreased to 28.6% (*p* = 0.23) and the pooled MD changed from −0.08 mm to −0.12 mm (95% CI −0.19 to −0.05), becoming statistically significant in favor of DCB (*p* < 0.01). This suggests that the direction of the LLL effect was consistent across studies, but the magnitude varied.

### Safety endpoints

#### Cardiac death

**Figure 6:**
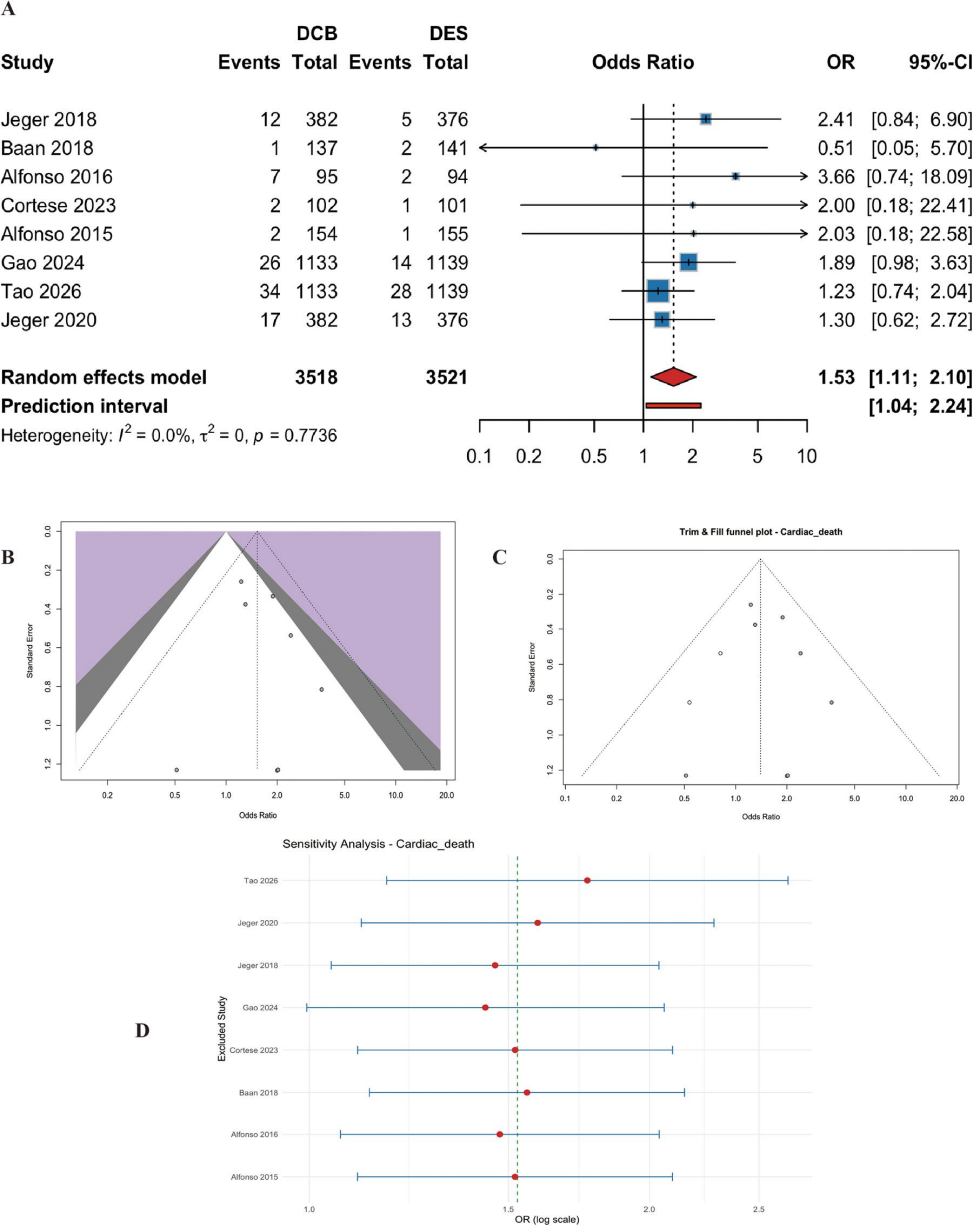
Meta-analysis results of cardiac death. Figure 6 presents the meta-analysis of cardiac death. **(A)** Forest plot comparing cardiac death between DCB and DES. **(B)** Heterogeneity assessment. **(C)** Trim-and-fill analysis. **(D)** Leave-one-out sensitivity analysis. DCB was associated with a significantly higher risk of cardiac death compared with DES (OR=1.53, 95% CI: 1.11–2.10, *P* = 0.009), with no heterogeneity (I^2^ = 0.0%).

Eight studies (*n* = 7039 patients) reported cardiac death. DCB significantly increased the risk of cardiac death compared with DES (OR=1.53, 95% CI: 1.11 to 2.10, *p* = 0.009) (Figure 6A). Heterogeneity was negligible (I^2^ = 0.0%, p for heterogeneity = 0.77) (Figure 6B). In a post-hoc exploratory subgroup analysis by DCB type, the increased risk of cardiac death was observed only in trials using paclitaxel-coated balloons (OR 1.63, 95% CI 1.17-2.28; 8 studies, *n* = 6,892), whereas trials using sirolimus-coated balloons showed no significant difference (OR 0.96, 95% CI 0.19-4.81; 2 studies, *n* = 231). This differential signal warrants cautious interpretation for several reasons: (1) the analysis was post-hoc and not prespecified; (2) the sirolimus-coated balloon trials had fewer events (only 3 cardiac deaths in total) and shorter follow-up (≤12 months vs. up to 36 months for paclitaxel trials); (3) the absolute event rates were low (2.2% for paclitaxel-DCB vs. 1.5% for DES), and the finding could represent a chance association; and (4) no such signal was observed for all-cause death. Therefore, while hypothesis-generating, this finding should not be overinterpreted as definitive evidence of a causal relationship without further validation from large-scale, long-term registries or patient-level meta-analyses.

#### Myocardial infarction (MI)

**Figure 7:**
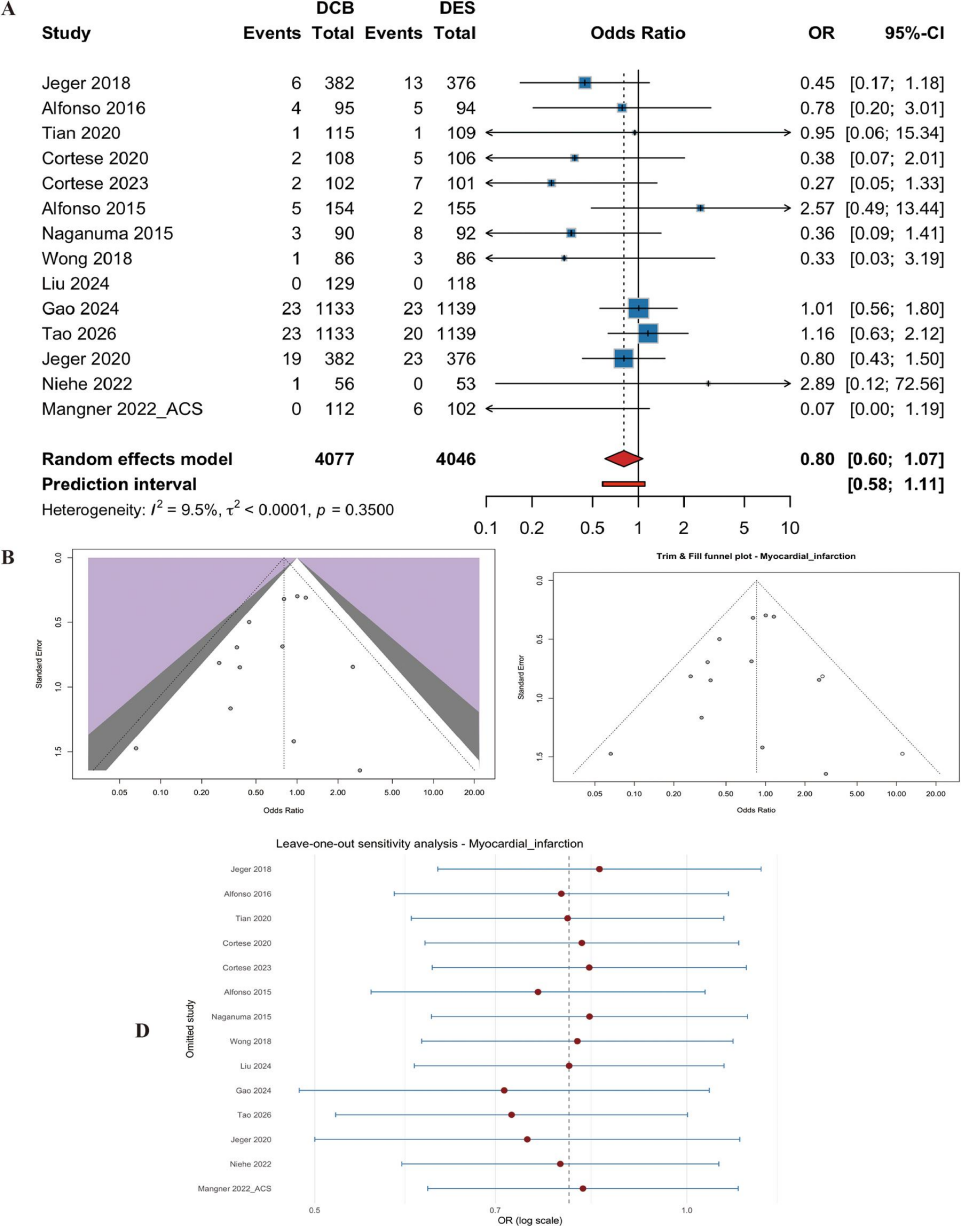
Meta-analysis results of myocardial infarction (MI). Figure 7 presents the meta-analysis of myocardial infarction. **(A)** Forest plot comparing MI between DCB and DES. **(B)** Heterogeneity assessment. **(C)** Trim-and-fill analysis. **(D)** Leave-one-out sensitivity analysis. No significant difference was found between the two groups (OR=0.80, 95% CI: 0.60–1.07, *P* = 0.136), with low heterogeneity (I^2^ = 9.5%).

Fourteen studies (*n* = 8123 patients) contributed to the analysis of MI. No significant difference was found between DCB and DES (OR=0.80, 95% CI: 0.60 to 1.07, *p* = 0.14) (Figure 7A). Heterogeneity was low (I^2^ = 9.5%, p for heterogeneity = 0.35) (Figure 7B). Egger's test suggested no publication bias (t = −1.55, df = 11, *p* = 0.15). The result remained unchanged after trim-and-fill and leave-one-out sensitivity analyses (Figures 7C,D).

#### All-cause death

**Figure 8:**
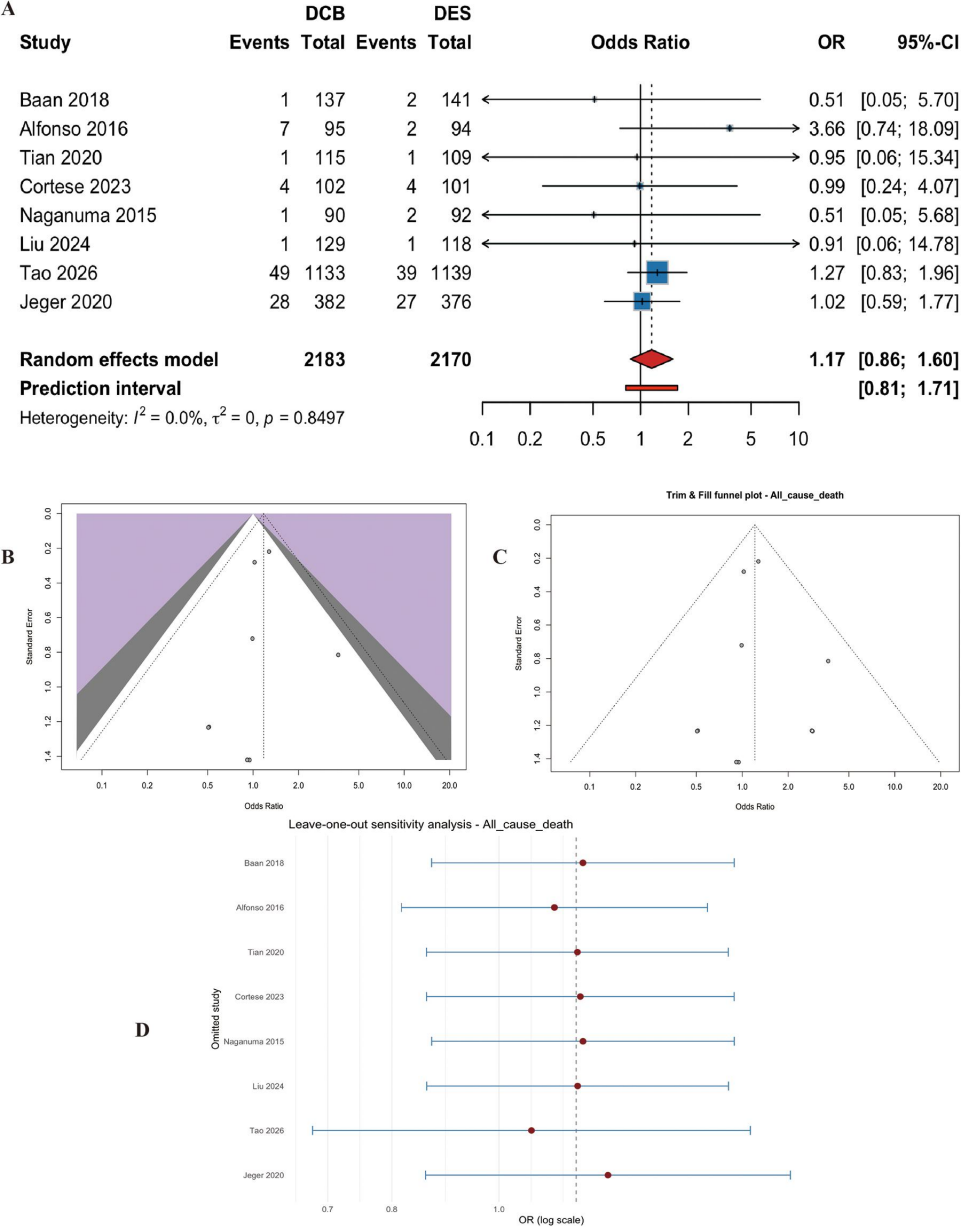
Meta-analysis results of All-cause death. Figure 8 presents the meta-analysis of all-cause death. **(A)** Forest plot comparing all-cause death between DCB and DES. **(B)** Heterogeneity assessment. **(C)** Trim-and-fill analysis. **(D)** Leave-one-out sensitivity analysis. No significant difference was observed between the two groups (OR=1.17, 95% CI: 0.86–1.60, *P* = 0.314), with no heterogeneity (I^2^ = 0.0%).

Eight studies (*n* = 4353 patients) reported all-cause death. There was no significant difference between the two groups (OR = 1.17, 95% CI: 0.8592 to 1.60, *p* = 0.31) (Figure 8A). Heterogeneity was absent (I^2^ = 0.0%, p for heterogeneity = 0.85) (Figure 8B). Egger's test was not significant (t = −0.50, df = 6, *p* = 0.64). Sensitivity analyses confirmed the robustness of the finding (Figures 8C,D).

#### Stent thrombosis

**Figure 9:**
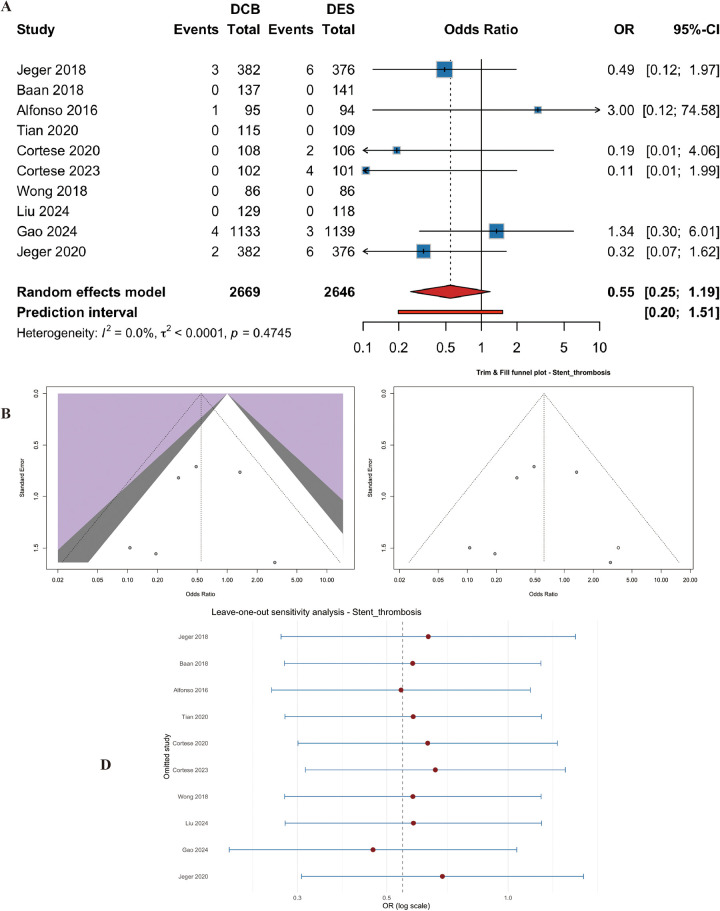
Meta-analysis results of stent thrombosis. Figure 9 presents the meta-analysis of stent thrombosis. **(A)** Forest plot comparing stent thrombosis between DCB and DES. **(B)** Heterogeneity assessment. **(C)** Trim-and-fill analysis. **(D)** Leave-one-out sensitivity analysis. The risk was numerically lower with DCB, but the difference did not reach statistical significance (OR=0.55, 95% CI: 0.25–1.19, *P* = 0.127), with no heterogeneity (I^2^ = 0.0%).

Ten studies (*n* = 5315 patients) reported thrombosis. The risk was numerically lower with DCB, but the difference did not reach statistical significance (OR = 0.55, 95% CI: 0.25 to 1.19, *p* = 0.13) (Figure 9A). Heterogeneity was low (I^2^ = 0.0%, p for heterogeneity = 0.47) (Figure 9B). Egger's test was not significant (t = −0.42, df = 4, *p* = 0.69). Trim-and-fill and leave-one-out analyses supported the stability of the estimate (Figures 9sC,D).

#### Device-oriented composite endpoint (DoCE)

**Figure 10:**
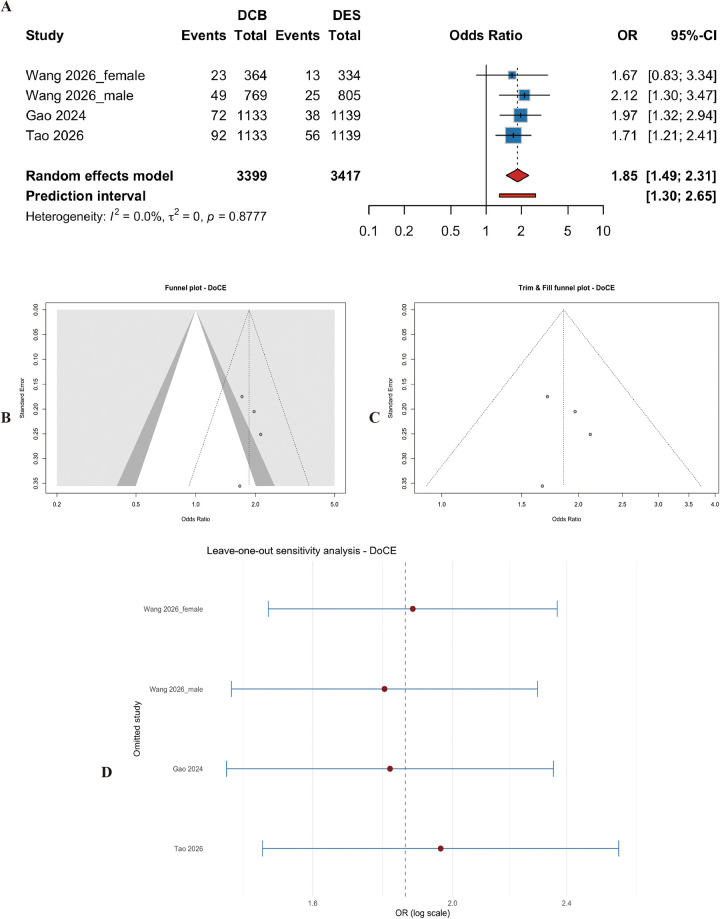
Meta-analysis results of device-oriented composite endpoint (DoCE). Figure 10 presents the meta-analysis of device-oriented composite endpoint. **(A)** Forest plot comparing DoCE between DCB and DES. **(B)** Heterogeneity assessment. **(C)** Trim-and-fill analysis. **(D)** Leave-one-out sensitivity analysis. DCB was associated with a significantly higher risk of DoCE compared with DES (OR=1.86, 95% CI: 1.49–2.31, *P* < 0.001), with no heterogeneity (I^2^ = 0.0%).

Four studies (*n* = 6816 patients) reported DoCE. DCB was associated with a significantly higher risk of DoCE compared with DES (OR = 1.86, 95% CI: 1.49 to 2.31, *p* < 0.001) (Figure 10A). Heterogeneity was low (I^2^ = 0.0%, p for heterogeneity = 0.88) (Figure 10B). Egger's test showed no asymmetry (t = 0.18, df = 2, *p* = 0.87). Sensitivity analyses confirmed the result (Figures 10C,D).

#### Patient-oriented composite endpoint (PoCE)

**Figure 11:**
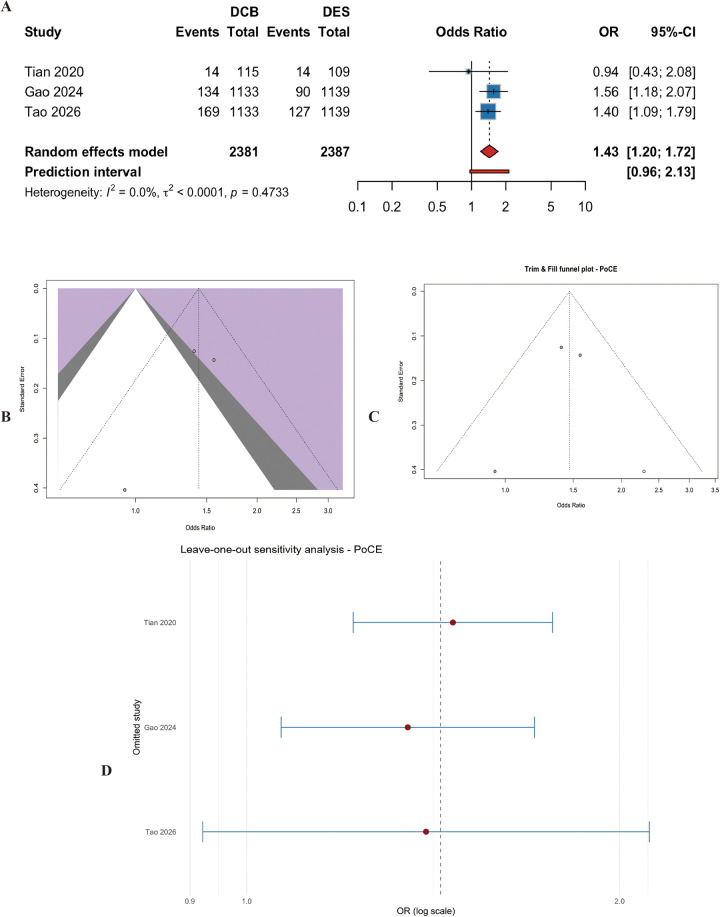
Meta-analysis results of patient-oriented composite endpoint (PoCE). Figure 11 presents the meta-analysis of patient-oriented composite endpoint. **(A)** Forest plot comparing PoCE between DCB and DES. **(B)** Heterogeneity assessment. **(C)** Trim-and-fill analysis. **(D)** Leave-one-out sensitivity analysis. DCB was associated with a significantly higher risk of PoCE compared with DES (OR=1.43, 95% CI: 1.20–1.72, *P* < 0.001), with no heterogeneity (I^2^ = 0.0%).

Three studies (*n* = 4768 patients) provided data on PoCE. DCB significantly increased the risk of PoCE compared with DES (OR = 1.43, 95% CI: 1.20 to 1.72, *p* < 0.001) (Figure 11A). Heterogeneity was low (I^2^ = 0.0%, p for heterogeneity = 0.47) (Figure 11B). Egger's test was not significant (t = −1.33, df = 1, *p* = 0.41). Leave-one-out analysis indicated that the result was robust (Figures 11C,D). Given the limited number of studies (k = 3), this analysis should be interpreted as exploratory.

#### Target lesion failure (TLF)

**Figure 12:**
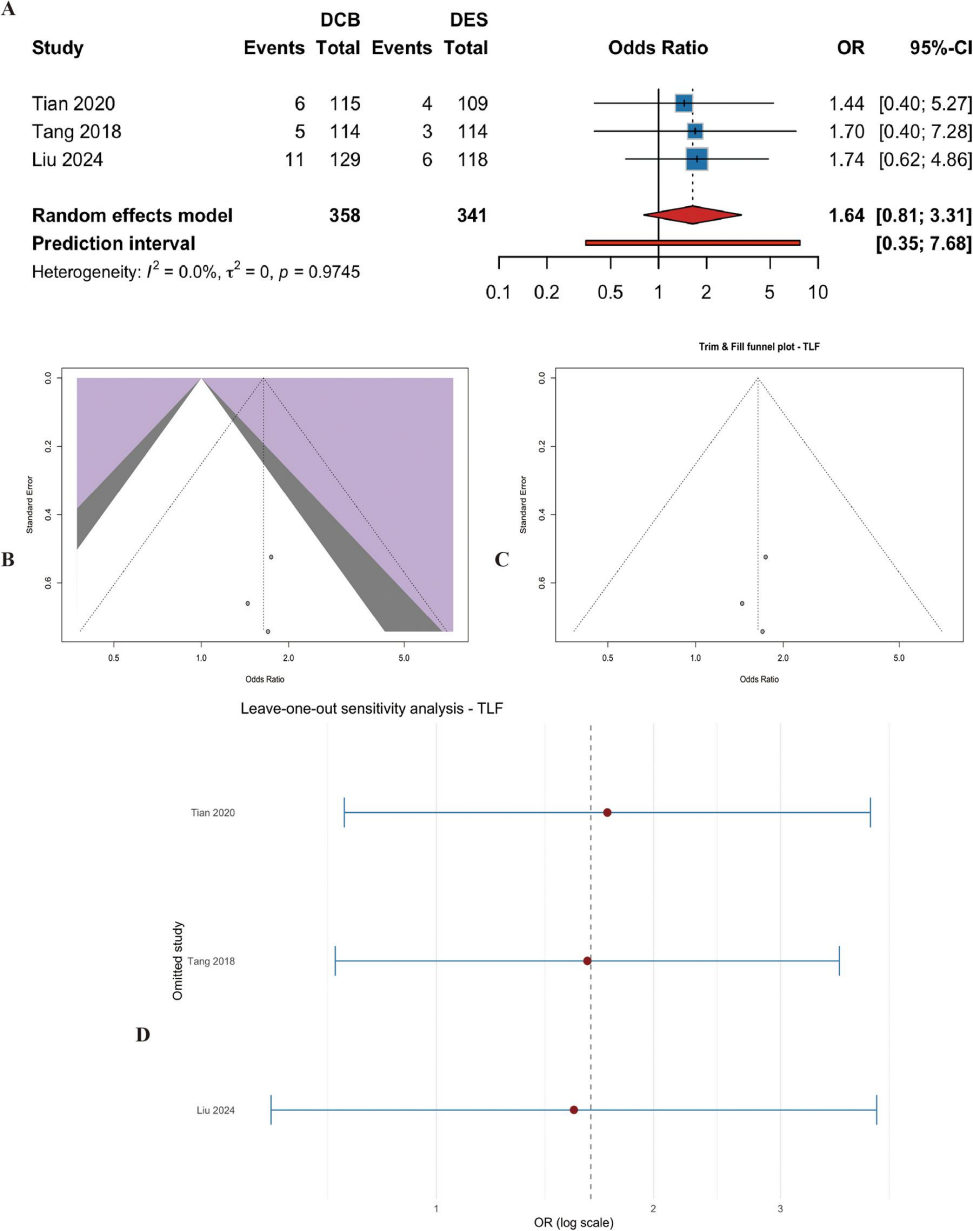
Meta-analysis results of target lesion failure (TLF). Figure 12 presents the meta-analysis of target lesion failure. **(A)** Forest plot comparing TLF between DCB and DES. **(B)** Heterogeneity assessment. **(C)** Trim-and-fill analysis. **(D)** Leave-one-out sensitivity analysis. The pooled estimate showed a higher risk with DCB, but the difference was not statistically significant (OR=1.64, 95% CI: 0.81–3.31, *P* = 0.170), with no heterogeneity (I^2^ = 0.0%).

Three studies (*n* = 699 patients) reported TLF. The pooled estimate showed a higher risk with DCB, but the difference was not statistically significant (OR = 1.63, 95% CI: 0.81 to 3.31, *p* = 0.1700) (Figure 12A). Heterogeneity was low (I^2^ = 0.0%, p for heterogeneity = 0.97) (Figure 12B). Egger's test was not significant (t = −0.47, df = 1, *p* = 0.72). Sensitivity analyses confirmed the stability of the finding (Figures 12C,D). This analysis is also exploratory due to the small number of included trials.

### Angiographic endpoints

#### Late lumen loss (LLL)

Six studies (*n* = 1081 patients) reported LLL. The mean difference (MD) between DCB and DES was −0.08 mm (95% CI: −0.18 to 0.02 mm, *p* = 0.10), indicating a trend toward less late loss with DCB that did not reach statistical significance (Supplementary Figure S1A). Substantial heterogeneity was observed (I^2^ = 73.4%, p for heterogeneity < 0.001). Egger's test was not performed due to the small number of studies. Leave-one-out sensitivity analysis suggested that the heterogeneity was driven by one study (Alfonso 2016), which showed a positive MD (DCB > DES) while all others favoured DCB (Supplementary Figure S1B-S1C).

#### Minimal lumen diameter (MLD)

Four studies (*n* = 1160 patients) reported MLD at follow-up. DES was associated with a significantly larger MLD compared with DCB (MD = −0.22 mm, 95% CI: −0.35 to −0.09 mm, *p* = 0.001) (Supplementary Figure S2A). Heterogeneity was substantial (I^2^ = 68.0%, p for heterogeneity = 0.02) (Supplementary Figure S2B). Sensitivity analysis indicated that the result was robust, with all studies favouring DES (Supplementary Figure S2C).

#### Hazard ratio-based analysis of DoCE (CKD and LAD subgroups)

Two studies provided adjusted hazard ratios for DoCE in patients with chronic kidney disease (CKD) or left anterior descending (LAD) artery lesions. The pooled HR was 2.17 (95% CI: 1.27 to 3.73, *p* < 0.001) Supplementary Figure S3A), indicating a significantly higher risk of DoCE with DCB in these high-risk subgroups. Heterogeneity was absent (I^2^ = 0.0%, p for heterogeneity = 0.34) (Supplementary Figure S3B-S3C).

#### Sensitivity and publication bias summary

For all binary outcomes, leave-one-out sensitivity analyses did not identify any single study that materially altered the pooled effect estimates. Detailed leave-one-out results for MACEs, TLR, and cardiac death are presented in Supplementary Table S4. Trim-and-fill analyses, where feasible, did not change the direction or significance of the results. Egger's tests were non-significant for all outcomes (all *p* > 0.05), suggesting no substantial small-study effects or publication bias. Table 2 showed the all details of Pooled Effect Estimates for All Outcomes.

**Table 2:** Pooled effect estimates for All outcomes.

Outcome	Studies (k)	Patients (n)	DCB Events/n (%)	DES Events/n (%)	Pooled OR/MD (95% CI)	*P* value	I^2^ (%)	P for Heterogeneity	Egger's Test (P)
Efficacy Endpoints									
MACE	11	3,621	168/1,813 (9.3)	150/1,808 (8.3)	0.92 (0.74–1.15)	0.470	10.4	0.345	0.968
TLR	11	6,828	245/3,416 (7.2)	115/3,412 (3.4)	2.22 (1.49–3.33)	<0.001	41.3	0.074	0.612
TLF	3	699	17/349 (4.9)	10/350 (2.9)	1.64 (0.81–3.31)	0.170	0.0	0.975	0.719
DoCE	4	6,816	164/3,408 (4.8)	94/3,408 (2.8)	1.86 (1.49–2.31)	<0.001	0.0	0.878	0.872
PoCE	3	4,768	303/2,384 (12.7)	217/2,384 (9.1)	1.43 (1.20–1.72)	<0.001	0.0	0.473	0.410
Safety Endpoints									
Cardiac death	8	7,039	78/3,524 (2.2)	52/3,515 (1.5)	1.53 (1.11–2.10)	0.009	0.0	0.774	0.488
Myocardial infarction	14	8,123	92/4,068 (2.3)	114/4,055 (2.8)	0.80 (0.60–1.07)	0.136	9.5	0.350	0.148
All-cause death	8	4,353	49/2,179 (2.2)	43/2,174 (2.0)	1.17 (0.86–1.60)	0.314	0.0	0.850	0.638
Thrombosis	10	5,315	7/2,660 (0.3)	13/2,655 (0.5)	0.55 (0.25–1.19)	0.127	0.0	0.475	0.695
Angiographic Endpoints									
Late lumen loss (mm)	6	1,081	—	—	−0.08 (−0.18 to 0.02)	0.102	73.4	0.002	NA
Minimal lumen diameter (mm)	4	1,160	—	—	−0.22 (−0.35 to −0.09)	0.001	68.0	0.025	NA
Subgroup: CKD and LAD lesions									
DoCE (CKD/LAD subgroups)	2	1,376	—	—	HR 2.17 (1.27–3.73)	0.005	0.0	0.344	NA

OR, odds ratio; MD, mean difference; HR, hazard ratio; CI, confidence interval; MACE, major adverse cardiac events; TLR, target lesion revascularization; TLF, target lesion failure; DoCE, device-oriented composite endpoint; PoCE, patient-oriented composite endpoint; NA, not applicable (insufficient studies for Egger's test).

#### Subgroup analyses

To explore potential sources of heterogeneity and assess the consistency of treatment effects across different clinical scenarios, we conducted subgroup analyses stratified by lesion type [*de novo* vs. in-stent restenosis (ISR)] and vessel size (small vessel vs. mixed vessel size). In addition, we performed exploratory subgroup analyses by clinical presentation (acute coronary syndrome vs. chronic coronary syndrome, and further by STEMI vs. NSTEMI vs. unstable angina when data were available) and by DCB type (paclitaxel vs. sirolimus).

### Subgroup analysis by lesion type

#### *De Novo* lesions

Seven studies comprising 5,660 patients (2,834 in the DCB group and 2,826 in the DES group) reported outcomes for *de novo* lesions. The pooled analysis demonstrated that DCB was associated with a significantly higher risk of target lesion revascularization (TLR) compared with DES (OR 1.76, 95% CI 1.03–3.02; *P* = 0.04). Moderate heterogeneity was observed across studies (*I*^2^ = 56.9%, *P* = 0.03; *τ*^2^ = 0.28) (Supplementary Figure S4A).

Individual study results showed variation in effect sizes, with three studies (34, 35) suggesting comparable or lower TLR rates with DCB (OR 0.98 and 0.55, respectively), while the remaining studies indicated higher TLR rates with DCB, particularly in the large-scale trials by Gao et al. (46) (OR 3.39, 95% CI 1.89–6.08) and Tao et al. (41) (OR 2.70, 95% CI 1.56–4.67), which contributed substantially to the pooled estimate due to their larger sample sizes and event counts.

#### In-Stent restenosis (ISR) lesions

Four studies with 1,168 patients (584 per group) focused on ISR lesions. The meta-analysis revealed that DES was superior to DCB (OR for TLR with DCB vs. DES 3.54, 95% CI 2.05–6.09; *P* < 0.001). Notably, no heterogeneity was detected among these studies (*I*^2^ = 0%, *P* = 0.971; *τ*^2^ = 0), indicating robust and reproducible findings across different study populations and designs. All individual studies demonstrated directionally consistent effects, with point estimates ranging from OR 3.16 (40) to OR 5.25 (45), though the latter had wide confidence intervals due to limited events (Supplementary Figure S4B).

#### Test for subgroup differences

The formal test for interaction between lesion type subgroups yielded a *Q* statistic of 3.19 with *P* = 0.07, suggesting a trend toward differential treatment effects between *de novo* and ISR lesions that did not reach conventional statistical significance. Despite the lack of statistical interaction, the consistently large effect size favoring DES in ISR lesions supports DES as the standard-of-care for this indication (Supplementary Figure S4C).

#### Subgroup analysis by vessel size

Table 3 showed the summary of subgroup findings.

**Table 3:** Summary of subgroup findings.

Subgroup	k	DCB Events/n	DES Events/n	Pooled OR (95% CI)	*P*-value	I^2^ (%)	Interaction P
Lesion Type							0.074
*De novo*	7	198/2,834	—/2,826	1.76 (1.03–3.02)	0.039	56.9	
ISR	4	77/584	—/584	3.54 (2.05–6.09)	<0.0001	0.0	
Vessel Size							—
Small vessel	5	69/568	—/548	1.17 (0.64–2.14)	0.604	15.1	
Clinical Presentation (MACE)							0.34
ACS	9	—	—	0.85 (0.56-1.29)	0.44	12.3	
CCS	9	—	—	0.96 (0.71-1.30)	0.79	0.0	
DCB Type (for TLR)							0.51
Paclitaxel	21	—	—	2.28 (1.52-3.42)	<0.001	42.1	
Sirolimus	2	—	—	1.85 (0.45-7.58)	0.39	0.0	

k, number of studies; OR, odds ratio; CI, confidence interval; DCB, drug-coated balloon; DES, drug-eluting stent; ISR, in-stent restenosis; TLR, target lesion revascularization; ACS, acute coronary syndrome; CCS, chronic coronary syndrome.

#### Small vessel disease

Five studies enrolling 1,116 patients (568 DCB, 548 DES) specifically examined small vessel interventions. The pooled analysis showed no significant difference in TLR between DCB and DES (OR 1.17, 95% CI 0.64–2.14; *P* = 0.60). Heterogeneity was low (*I*^2^ = 15.1%, *P* = 0.32; *τ*^2^ = 0.12), with study results generally clustering around the null effect, though Liu et al. (9) and Tian et al. (33) showed nonsignificant trends favoring DES, while Cortese et al. (35) suggested a potential benefit with DCB (OR 0.55) (Supplementary Figure S4D).

#### Exploratory subgroup analysis by clinical presentation

Data on clinical presentation were available from 9 studies that explicitly reported outcomes for acute coronary syndrome (ACS) vs. chronic coronary syndrome (CCS). The pooled analysis for MACEs showed no significant interaction between clinical presentation and treatment effect (P-interaction = 0.34). When further stratified by STEMI (REVELATION trial, *n* = 120) vs. NSTEMI (BASKET-SMALL 2 ACS subgroup, *n* = 214) vs. unstable angina (various), the number of events was too small to draw meaningful conclusions; the confidence intervals were wide and overlapping, indicating no clear differential effect. These analyses are hypothesis-generating and limited by low statistical power.

#### Exploratory subgroup analysis by DCB type

Twenty-one trials used paclitaxel-coated balloons, and two recent trials used sirolimus-coated balloons. For the primary efficacy endpoint (TLR), the effect appeared similar between the two types (P-interaction = 0.51). However, for cardiac death, the increased risk was only observed in the paclitaxel group (OR 1.63, 95% CI 1.17-2.28), while the sirolimus group showed no signal (OR 0.96, 95% CI 0.19-4.81), albeit with very limited data and short follow-up.

The subgroup analyses revealed important clinical insights. The divergent treatment effects by lesion type suggest that for ISR, DES offers superior efficacy compared to DCB, likely due to the durable mechanical scaffolding and sustained antiproliferative release that are critical in the hostile neoatherosclerotic environment. Conversely, in *de novo* lesions—particularly in larger vessels or more complex anatomies—DES also appeared superior to DCB in preventing repeat revascularization, attributable to the mechanical scaffolding that prevents early recoil and negative remodeling.

The absence of significant interaction (*P* = 0.074) should be interpreted cautiously given the substantial difference in point estimates and the limited statistical power for interaction testing. The trend toward differential efficacy warrants consideration in clinical decision-making, with lesion type potentially serving as a treatment-effect modifier.

In small vessel disease, the comparable TLR rates between DCB and DES support the viability of DCB as a “leave-nothing-behind” strategy, particularly in anatomical contexts where stent placement may be challenging or where long-term stent-related events (such as very late stent thrombosis or fracture in tortuous segments) are of concern. The low heterogeneity in this subgroup strengthens confidence in this finding.

## Discussion

This systematic review and meta-analysis of 23 randomized controlled trials, encompassing 8,123 patients, represents the most comprehensive contemporary synthesis comparing DCB with DES for coronary artery disease. Our analysis yields several pivotal findings that refine the clinical landscape for these technologies. First, while DCB was associated with a comparable risk of MACEs and myocardial infarction MI to DES, it was linked to a significantly higher risk of TLR, DoCE, and PoCE. This finding contrasts with two recent meta-analyses (21, 22) that reported no significant difference in TLR between DCB and DES for *de novo* lesions. The discrepancy is likely explained by the inclusion of the REC-CAGEFREE I trial in our analysis, which is the largest trial to date and showed a clear disadvantage of DCB in non-small vessels. Second, and most critically, this overall effect masked profound heterogeneity based on lesion type: DES demonstrated a strong, consistent superiority over DCB for ISR (OR 3.54, favoring DES), yet a significantly higher risk of TLR with DCB was observed in *de novo* lesions. Third, in small vessel disease, the two strategies yielded comparable TLR rates. Fourth, a concerning signal of increased cardiac death with DCB emerged, which appears to be driven by paclitaxel-coated balloons, a finding that warrants careful scrutiny and further investigation.

The most striking finding of this meta-analysis is the consistent superiority of DES over DCB in ISR lesions. For patients with ISR, DES was associated with a threefold lower odds of TLR compared to DCB (OR 3.54, 95% CI 2.05–6.09, favoring DES). This finding, supported by zero statistical heterogeneity, establishes DES as the preferred, evidence-based strategy for this challenging clinical scenario. The mechanistic rationale is compelling: in ISR, the goal is to avoid adding a second or third layer of metal, which can precipitate neoatherosclerosis, lumen compromise, and future complex restenosis. The homogeneous drug delivery of a DCB, without a permanent implant, effectively treats the proliferative process while preserving the original scaffold (49, 50). This conclusion is robustly supported by landmark trials such as the RIBS IV and V studies and the DARE trial, all of which consistently demonstrated the clinical benefit of DES in this context.

Conversely, in *de novo* lesions, the analysis revealed a starkly different reality: DES was associated with a significantly lower risk of TLR compared to DCB (OR 1.76, 95% CI 1.03–3.02). This finding, driven by large-scale trials like REC-CAGEFREE I (51, 52), suggests that the mechanical scaffolding provided by DES remains essential for optimal long-term patency in untreated vessels. The higher TLR rates with DCB in *de novo* lesions likely reflect the inherent limitations of a “leave-nothing-behind” strategy in this setting. The absence of a permanent scaffold means that the acute gain achieved by balloon angioplasty is vulnerable to early recoil and negative remodeling, processes that are effectively eliminated by DES (51). Furthermore, the presence of a flow-limiting dissection or suboptimal post-dilation result after DCB necessitates “bailout” stenting, a scenario that often leads to a more complex, hybrid procedure with potentially less predictable outcomes.

The analysis of SVD represents an important nuance to the *de novo* lesion finding. In SVD, where the use of DES is often limited by higher rates of restenosis and technical challenges, DCB performed comparably to DES for TLR (OR 1.17, 95% CI 0.64–2.14). This finding aligns with the results of the BASKET-SMALL 2 and PICCOLETO II trials, which established the non-inferiority of DCB in this specific anatomic subset. The comparability in SVD may be explained by the fact that in small vessels, the absolute risk of restenosis is higher for both technologies, potentially narrowing the margin of benefit conferred by a DES (53). Additionally, the avoidance of a metallic scaffold in tortuous or diffusely diseased small vessels may reduce the risk of long-term complications such as stent fracture or flow disturbances, making DCB a particularly attractive and clinically viable option (54).

A finding that warrants significant attention—but also cautious interpretation—is the pooled analysis demonstrating a 53% higher risk of cardiac death with DCB compared to DES (OR 1.53, 95% CI 1.11–2.10). Our post-hoc subgroup analysis revealed that this signal was exclusively observed in trials using paclitaxel-coated balloons, whereas sirolimus-coated balloons showed no such signal. However, this finding is exploratory and subject to several limitations. First, the absolute event rates were low (2.2% vs. 1.5%), and the difference was driven by a small number of excess events. Second, the sirolimus-coated balloon trials were smaller, had shorter follow-up, and contained too few events to draw any meaningful comparison. Third, no signal was seen for all-cause death, which would be expected if paclitaxel had a true mortality effect. A potential mechanism, though speculative, could relate to the very late mortality signal previously reported with paclitaxel-coated devices in peripheral artery interventions (55), but this remains unproven in the coronary setting. Thus, while this signal cannot be dismissed, it should be interpreted with considerable caution and viewed as hypothesis-generating rather than definitive. Dedicated long-term follow-up studies and individual patient data meta-analyses are urgently needed to clarify whether this represents a true safety concern or a statistical artifact (50).

The subgroup analyses revealed important clinical insights. The divergent treatment effects by lesion type suggest that for ISR, DES offers superior efficacy compared to DCB, likely due to the durable mechanical scaffolding and sustained antiproliferative release that are critical in the hostile neoatherosclerotic environment. Conversely, in *de novo* lesions—particularly in larger vessels or more complex anatomies—DES also appeared superior to DCB in preventing repeat revascularization, attributable to the mechanical scaffolding that prevents early recoil and negative remodeling. The absence of significant interaction (*P* = 0.07) should be interpreted cautiously given the substantial difference in point estimates and the limited statistical power for interaction testing. The trend toward differential efficacy warrants consideration in clinical decision-making, with lesion type potentially serving as a treatment-effect modifier. In small vessel disease, the comparable TLR rates between DCB and DES support the viability of DCB as a “leave-nothing-behind” strategy, particularly in anatomical contexts where stent placement may be challenging or where long-term stent-related events (such as very late stent thrombosis or fracture in tortuous segments) are of concern. The low heterogeneity in this subgroup strengthens confidence in this finding.

### Comparison with recent meta-analyses

Two recent meta-analyses have addressed similar questions. Haq et al. (21) (13 RCTs, *n* = 4,686) reported no significant differences between DCB and DES for TLR (RR 1.19, 95% CI 0.64-2.21) or cardiac death (RR 1.33, 95% CI 0.86-2.05) in *de novo* lesions. Rinaldi et al. (22) (13 RCTs, *n* = 7,776) found comparable TLR rates (RR 1.24, 95% CI 0.79-1.95) and a significant reduction in major bleeding with DCB. The key difference between these analyses and our study is the inclusion of the REC-CAGEFREE I trial, which alone contributed over 2,200 patients and demonstrated a clear disadvantage of DCB in non-small *de novo* lesions. Therefore, our updated meta-analysis provides a more complete and contemporary estimate, revealing that the overall comparability in prior analyses was likely due to the predominance of small-vessel studies. When all vessel sizes are considered, DES remains superior for preventing TLR in *de novo* lesions, except in the small-vessel subset. Our study also uniquely reports the lesion-specific interaction and the paclitaxel-related cardiac death signal, which were not explored in prior meta-analyses.

The strengths of this meta-analysis are substantial. It is the largest and most up-to-date synthesis, incorporating the most recent and expansive trials, including the all-comer REC-CAGEFREE I study. The rigorous methodology, including comprehensive database searches, adherence to PRISMA guidelines, and use of the Cochrane RoB 2 tool, ensures methodological soundness. We have provided a fully reproducible R script, detailed leave-one-out results, and weighted risk of bias assessments to enhance transparency. The exploration of heterogeneity through prespecified subgroup analyses—particularly by lesion type and vessel size—provides actionable clinical insights rather than a singular, potentially misleading pooled estimate (56). Furthermore, the sensitivity and publication bias analyses confirmed the robustness of the core findings.

Nevertheless, this study has important limitations. The most significant is the inherent clinical heterogeneity across the included trials, which varied in lesion characteristics (*de novo* vs. ISR, vessel size), patient populations (stable CAD vs. ACS), DCB platforms (paclitaxel vs. sirolimus), and bailout stenting protocols. Although random-effects models were employed and subgroup analyses were performed to mitigate this, residual confounding is possible (57). Second, the open-label design of all included trials introduces a risk of performance bias, particularly for subjective endpoints like revascularization. However, the consistent use of blinded clinical event committees across most studies likely mitigated detection bias. Third, the follow-up duration varied, with most trials providing data only up to 12 or 24 months. The long-term durability of the DCB effect beyond three years remains uncertain, particularly for safety endpoints like cardiac death (58). Fourth, the analyses for TLF and PoCE were based on a limited number of studies (k ≤ 4), and these results should be interpreted as exploratory. Fifth, the exploratory subgroup analyses by clinical presentation (STEMI/NSTEMI/unstable angina) were severely underpowered due to the small number of events, and no firm conclusions can be drawn. Sixth, the definition of MACEs and other composite endpoints was not uniform across all studies, requiring us to pool them under a common definition, which may introduce some inconsistency. Seventh, the cardiac death signal by DCB type was derived from a post-hoc analysis and requires confirmation in dedicated trials. Lastly, the availability of patient-level data would have allowed for more sophisticated analyses, such as time-to-event modeling and adjustment for baseline covariates, but such data were not uniformly available (59).

These findings have direct and actionable implications for clinical practice. For patients with ISR, this meta-analysis unequivocally supports DES as the treatment of choice, offering superior efficacy in preventing repeat revascularization. For patients with *de novo* small vessel disease, DCB represents a safe and effective alternative to DES, particularly when the goal is to avoid a permanent implant (60). However, for *de novo* lesions in larger, non-small vessels, DES remains the standard of care, providing superior durable patency and freedom from repeat revascularization. The signal for increased cardiac death with paclitaxel-coated balloons, while requiring further validation, should temper enthusiasm for a “DCB-for-all” strategy and reinforces the need for careful patient selection and device-specific consideration (61). In conclusion, this meta-analysis demonstrates that the comparative effectiveness of DCB vs. DES is not uniform but is critically dependent on the clinical context. DES is the superior strategy for ISR, and DCB is a valid alternative to DES for *de novo* small vessel disease. For *de novo* lesions in larger vessels, DES remains the superior approach. The finding of increased cardiac death with paclitaxel-coated balloons is a novel and concerning signal that mandates urgent investigation in future long-term studies and pooled patient-level analyses (62). As the field moves towards a more personalized approach to coronary revascularization, these data provide a robust evidence base to guide the selection of the optimal device based on patient and lesion characteristics.

## Conclusion

This meta-analysis shows that the comparative effectiveness of DCB vs. DES is lesion-specific. For in-stent restenosis, DES is superior and remains the preferred strategy. In *de novo* small vessel disease, DCB is a comparable alternative. However, in *de novo* lesions of larger vessels, DES provides superior freedom from repeat revascularization. The increased cardiac death signal associated with paclitaxel-coated balloons, observed in exploratory post-hoc analyses, warrants further investigation and should be interpreted with caution given the limited event numbers and shorter follow-up in sirolimus-coated balloon trials. These findings support a personalized, lesion-driven approach to coronary revascularization .

## Data Availability

The original contributions presented in the study are included in the article/Supplementary Material, further inquiries can be directed to the corresponding author/s.

## Supplementary material

SUPPLEMENTARY FIGURE S1

Subgroup analysis results of Late Lumen Loss (LLL). Supplementary Figure 1 presents the meta-analysis of late lumen loss. (A) Forest plot comparing late lumen loss (mm) between DCB and DES. (B) Heterogeneity assessment. (C) Leave-one-out sensitivity analysis. The mean difference was −0.08 mm (95% CI: −0.18 to 0.02 mm, *P* = 0.102), indicating a trend toward less late loss with DCB that did not reach statistical significance. Substantial heterogeneity was observed (I^2^ = 73.4%).

SUPPLEMENTARY FIGURE S2

Subgroup analysis results of Minimal Lumen Diameter (MLD). Supplementary Figure 2 presents the meta-analysis of minimal lumen diameter. (A) Forest plot comparing minimal lumen diameter (mm) between DCB and DES. (B) Heterogeneity assessment. (C) Leave-one-out sensitivity analysis. DES was associated with a significantly larger MLD compared with DCB (MD = −0.22 mm, 95% CI: −0.35 to −0.09 mm, *P* = 0.001), with substantial heterogeneity (I^2^ = 68.0%).

SUPPLEMENTARY FIGURE S3

Subgroup analysis results of Hazard Ratio for DoCE in CKD and LAD Subgroups. Supplementary Figure 3 presents the pooled hazard ratio analysis for device-oriented composite endpoint in high-risk subgroups. (A) Forest plot of pooled hazard ratios for DoCE in patients with chronic kidney disease (CKD) or left anterior descending (LAD) artery lesions. (B) Heterogeneity assessment. (C) Sensitivity analysis. The pooled HR was 2.17 (95% CI: 1.27–3.73, *P* = 0.005), indicating a significantly higher risk of DoCE with DCB in these subgroups, with no heterogeneity (I^2^ = 0.0%).

SUPPLEMENTARY FIGURE S4

Subgroup Analyses for Target Lesion Revascularization (TLR). Supplementary Figure 4 presents subgroup analyses for target lesion revascularization stratified by lesion type and vessel size. (A) Forest plot for *de novo* lesions. (B) Forest plot for in-stent restenosis (ISR) lesions. (C) Test for subgroup differences. (D) Forest plot for small vessel disease. DCB was associated with a significantly higher risk of TLR in *de novo* lesions (OR = 1.76, 95% CI: 1.03–3.02) but was superior to DES in ISR lesions (OR = 3.54, 95% CI: 2.05–6.09). In small vessel disease, no significant difference was observed (OR = 1.17, 95% CI: 0.64–2.14).

SUPPLEMENTARY eFile 1

R analysis Results (“DCB_vs_DES_meta_analysis.R”). Cross-check confirmation: All supplementary files have been verified against the main text for correct citation and completeness.
