# Supplementary material for: Drug-coated balloons vs. drug-eluting stents for coronary artery disease: an updated systematic review and meta-analysis of randomized controlled trials with lesion-specific insights
Source: Front Cardiovasc Med. 2026 May 18;13:1843262. doi: 10.3389/fcvm.2026.1843262 (PMC13223156; doi:10.3389/fcvm.2026.1843262)
Supplement: Supplementary file 1 [file Table1.docx]

**Supplementary Table 1: Detailed Search Strategy**

| **Database** | **Search Date** | **Search Terms** | **Hits** |
| --- | --- | --- | --- |
| PubMed/MEDLINE | March 2026 | ((("drug coated balloon"[Title/Abstract] OR "drug-coated balloon"[Title/Abstract] OR "DCB"[Title/Abstract] OR "drug eluting balloon"[Title/Abstract] OR "drug-eluting balloon"[Title/Abstract] OR "DEB"[Title/Abstract] OR "paclitaxel coated balloon"[Title/Abstract] OR "paclitaxel-eluting balloon"[Title/Abstract] OR "SeQuent Please"[Title/Abstract] OR "IN.PACT"[Title/Abstract] OR "RESTORE"[Title/Abstract] OR "BASKET"[Title/Abstract]) AND ("drug eluting stent"[Title/Abstract] OR "drug-eluting stent"[Title/Abstract] OR "DES"[Title/Abstract] OR "sirolimus eluting stent"[Title/Abstract] OR "everolimus eluting stent"[Title/Abstract] OR "zotarolimus eluting stent"[Title/Abstract] OR "biolimus eluting stent"[Title/Abstract] OR "Xience"[Title/Abstract] OR "Promus"[Title/Abstract] OR "Resolute"[Title/Abstract] OR "Synergy"[Title/Abstract])) AND ("coronary artery disease"[Title/Abstract] OR "coronary heart disease"[Title/Abstract] OR "CAD"[Title/Abstract] OR "CHD"[Title/Abstract] OR "coronary stenosis"[Title/Abstract] OR "coronary lesion"[Title/Abstract] OR "coronary atherosclerosis"[Title/Abstract] OR "ischemic heart disease"[Title/Abstract] OR "coronary revascularization"[Title/Abstract] OR "percutaneous coronary intervention"[Title/Abstract] OR "PCI"[Title/Abstract] OR "coronary angioplasty"[Title/Abstract])) AND ("randomized controlled trial"[Publication Type] OR "randomized"[Title/Abstract] OR "randomised"[Title/Abstract] OR "randomly"[Title/Abstract] OR "RCT"[Title/Abstract] OR "clinical trial"[Title/Abstract] OR "controlled trial"[Title/Abstract]) | 1,892 |
| Embase | March 2026 | ('drug coated balloon'/exp OR 'drug-coated balloon' OR DCB OR 'drug eluting balloon'/exp OR 'drug-eluting balloon' OR DEB OR 'paclitaxel coated balloon' OR 'paclitaxel-eluting balloon' OR SeQuent Please OR IN.PACT OR RESTORE OR BASKET) AND ('drug eluting stent'/exp OR 'drug-eluting stent' OR DES OR 'sirolimus eluting stent'/exp OR everolimus eluting stent OR zotarolimus eluting stent OR biolimus eluting stent OR Xience OR Promus OR Resolute OR Synergy) AND ('coronary artery disease'/exp OR 'coronary heart disease'/exp OR CAD OR CHD OR 'coronary stenosis'/exp OR 'coronary lesion'/exp OR 'coronary atherosclerosis'/exp OR 'ischemic heart disease'/exp OR 'coronary revascularization'/exp OR 'percutaneous coronary intervention'/exp OR PCI OR 'coronary angioplasty'/exp) AND (randomized controlled trial OR random*:ti,ab OR RCT) | 1,756 |
| CENTRAL | March 2026 | #1 MeSH descriptor: [Drug-Eluting Stents] explode all trees  2 MeSH descriptor: [Stents] explode all trees  3 (drug-eluting stent* or DES or sirolimus-eluting stent* or everolimus-eluting stent* or zotarolimus-eluting stent* or biolimus-eluting stent* or Xience or Promus or Resolute or Synergy):ti,ab,kw  4 #1 OR #2 OR #3  5 MeSH descriptor: [Angioplasty, Balloon, Coronary] explode all trees  6 MeSH descriptor: [Balloon Dilatation] explode all trees  7 (drug-coated balloon* or DCB or paclitaxel-coated balloon* or paclitaxel-eluting balloon* or SeQuent Please or IN.PACT or RESTORE or BASKET):ti,ab,kw  8 #5 OR #6 OR #7  9 #4 AND #8  10 MeSH descriptor: [Coronary Artery Disease] explode all trees  11 MeSH descriptor: [Myocardial Ischemia] explode all trees  12 (coronary artery disease* or CAD or CHD or coronary stenosis* or coronary lesion* or coronary atherosclerosis* or ischemic heart disease* or coronary revascularization* or percutaneous coronary intervention* or PCI or coronary angioplasty*):ti,ab,kw  13 #10 OR #11 OR #12  14 #9 AND #13  15 Limit 14 to Randomized Controlled Trials \| 639 \| | 639 |
| Web of Science | March 2026 | TS=((("drug coated balloon" OR "drug-coated balloon" OR DCB OR "drug eluting balloon" OR "drug-eluting balloon" OR DEB OR "paclitaxel coated balloon" OR "paclitaxel-eluting balloon" OR SeQuent Please OR IN.PACT OR RESTORE OR BASKET) AND ("drug eluting stent" OR "drug-eluting stent" OR DES OR "sirolimus eluting stent" OR everolimus eluting stent OR zotarolimus eluting stent OR biolimus eluting stent OR Xience OR Promus OR Resolute OR Synergy)) AND ("coronary artery disease" OR CAD OR CHD OR "coronary stenosis" OR "coronary lesion" OR "coronary atherosclerosis" OR "ischemic heart disease" OR "coronary revascularization" OR PCI OR "coronary angioplasty")) AND DT=(Article) AND PY=(2012-2026) \| 2,584 \| |  |

*Note.* Full search strategies available upon request.
