# Supplementary material for: Drug-coated balloons vs. drug-eluting stents for coronary artery disease: an updated systematic review and meta-analysis of randomized controlled trials with lesion-specific insights
Source: Front Cardiovasc Med. 2026 May 18;13:1843262. doi: 10.3389/fcvm.2026.1843262 (PMC13223156; doi:10.3389/fcvm.2026.1843262)
Supplement: Supplementary file 2 [file Table2.docx]

**Supplementary Table 2: Risk of Bias Assessment Details**

| **Study** | **D1: Randomization** | **D2: Deviations** | **D3: Missing Data** | **D4: Measurement** | **D5: Selection** | **Overall** | **Weight (%)** | **Key Supporting Evidence** |
| --- | --- | --- | --- | --- | --- | --- | --- | --- |
| Wang et al. 2026 (REC-CAGEFREE I) | Low | Some concerns | Low | Low | Low | Some concerns | 25.5 | Central web-based randomization; open-label; blinded CEC; ITT analysis; 99.4% follow-up |
| Jeger et al. 2018 (BASKET-SMALL 2) | Low | Some concerns | Low | Low | Low | Some concerns | 18.3 | Interactive web system; open-label; blinded CEC; 96% follow-up; DES switch documented |
| Jeger et al. 2020 (BASKET-SMALL 2, 3-year) | Low | Some concerns | Low | Low | Low | Low | 15.2 | Prespecified long-term follow-up; 91% 3-year follow-up; blinded CEC maintained |
| Wong et al. 2017 (RESTORE) | Low | High | High | Low | Low | High | 2.1 | Early termination (66% enrollment); 44% angiographic follow-up; open-label |
| Alfonso et al. 2015 | Low | Some concerns | Some concerns | Low | Low | Some concerns | 3.2 | Telephone randomization; open-label; 100% clinical follow-up; 82% angiographic follow-up |
| Alfonso et al. 2016 | Low | Some concerns | Low | Low | Low | Some concerns | 2.8 | Same as above |
| Alfonso et al. 2019 | Low | Some concerns | Low | Low | Low | Some concerns | 3.5 | Same as above |
| Cortese et al. 2020 | Low | Some concerns | Low | Low | Low | Some concerns | 4.2 | Central randomization; open-label; blinded core lab; 90% follow-up |
| Cortese et al. 2023 | Low | Some concerns | Low | Low | Low | Some concerns | 4.8 | Same as above |
| Tang et al. 2018 | Some concerns | Some concerns | Low | Low | Low | Some concerns | 3.1 | Insufficient stratification details; open-label; blinded CEC |
| Liu et al. 2024 (DISSOLVE SVD) | Low | Some concerns | Some concerns | Low | Low | Some concerns | 3.6 | Interactive web system; open-label; 87% angiographic follow-up; blinded core lab |
| Gao et al. 2024 (REC-CAGEFREE I, 3-year) | Low | Some concerns | Low | Low | Low | Low | 12.4 | Prespecified 3-year analysis; 97% follow-up; blinded CEC |
| Tao et al. 2023 | Low | Some concerns | Low | Low | Low | Some concerns | 8.7 | Same as REC-CAGEFREE I main trial |
| Niehe et al. 2022 (REVELATION, 2-year) | Low | Some concerns | Low | Low | Low | Some concerns | 1.8 | Single-center; open-label; blinded CEC; 91% 2-year follow-up |
| Cortese et al. 2016 (PICCOLETO II) | Low | Some concerns | Low | Some concerns | Low | Some concerns | 2.9 | Central randomization; open-label; unblinded investigators for some endpoints; 90% follow-up |
| Cortese et al. 2023 (PICCOLETO II, 3-year) | Low | Some concerns | Low | Low | Low | Low | 2.7 | Prespecified long-term follow-up; 87% 3-year follow-up |
| Jeger et al. 2022 (BASKET-SMALL 2, ACS) | Low | Some concerns | Low | Low | Low | Some concerns | 3.3 | Prespecified subgroup; same methodology as main trial |
| Jeger et al. 2021 (BASKET-SMALL 2, DM) | Low | Some concerns | Low | Low | Low | Some concerns | 3.0 | Prespecified subgroup; same methodology as main trial |
| Alfonso et al. 2015 (RIBS IV) | Low | Some concerns | Low | Low | Low | Some concerns | 3.4 | Central telephone randomization; open-label; blinded core lab; 90% follow-up |
| Alfonso et al. 2016 (RIBS V) | Low | Some concerns | Some concerns | Low | Low | Some concerns | 2.6 | 18% angiographic loss to follow-up at 6 months; 100% clinical follow-up |
| Alfonso et al. 2016 (RIBS IV+V pooled) | Low | Some concerns | Low | Low | Low | Low | 3.9 | IPD meta-analysis; consistent methodology; blinded core lab |
| Wang et al. 2015 (BELLO) | Low | Some concerns | Some concerns | Low | Low | Some concerns | 2.2 | Single-blind design; 97.8% 2-year follow-up |
| Cheng et al. 2026 (REC-CAGEFREE I, LAD) | Low | Some concerns | Low | Some concerns | Some concerns | Some concerns | 2.8 | Prespecified subgroup; IPTW adjustment; blinded CEC; post-hoc endpoint modification noted |

*Note.* D1–D5 = Cochrane Risk of Bias 2.0 domains; CEC = clinical event committee; ITT = intention-to-treat; IPTW = inverse probability of treatment weighting; IPD = individual patient data.
