# Supplementary material for: Drug-coated balloons vs. drug-eluting stents for coronary artery disease: an updated systematic review and meta-analysis of randomized controlled trials with lesion-specific insights
Source: Front Cardiovasc Med. 2026 May 18;13:1843262. doi: 10.3389/fcvm.2026.1843262 (PMC13223156; doi:10.3389/fcvm.2026.1843262)
Supplement: Supplementary file 3 [file Table3.docx]

**Supplementary Table 3: Sensitivity Analyses Excluding Studies by Risk of Bias**

| **Analysis** | **Studies Excluded** | **MACE OR (95% CI)** | **MACE P value** | **TLR OR (95% CI)** | **TLR P value** | **DoCE OR (95% CI)** | **DoCE P value** |
| --- | --- | --- | --- | --- | --- | --- | --- |
| Primary analysis (all studies) | None | 0.92 (0.74–1.15) | 0.470 | 2.22 (1.49–3.33) | <0.001 | 1.86 (1.49–2.31) | <0.001 |
| Excluding high-risk studies | Wong et al. 2017 | 0.89 (0.71–1.12) | 0.320 | 2.18 (1.45–3.28) | <0.001 | 1.84 (1.48–2.29) | <0.001 |
| Excluding high + D3 "some concerns" | Wong et al. 2017, Alfonso 2015, Liu 2024, Wang 2015 | 0.91 (0.72–1.15) | 0.445 | 2.15 (1.42–3.25) | <0.001 | 1.85 (1.48–2.31) | <0.001 |
| Only low-risk studies | All except Jeger 2020, Gao 2024, Cortese 2023, RIBS IV+V | 0.85 (0.65–1.11) | 0.230 | 2.05 (1.32–3.18) | 0.001 | 1.82 (1.45–2.28) | <0.001 |

*Note.* D3 = Domain 3 (Missing Outcome Data). All sensitivity analyses used random-effects model with REML estimation.
