# Supplementary material for: Drug-coated balloons vs. drug-eluting stents for coronary artery disease: an updated systematic review and meta-analysis of randomized controlled trials with lesion-specific insights
Source: Front Cardiovasc Med. 2026 May 18;13:1843262. doi: 10.3389/fcvm.2026.1843262 (PMC13223156; doi:10.3389/fcvm.2026.1843262)
Supplement: Supplementary file 4 [file Table4.docx]

**Supplementary Table 4: Detailed Subgroup Analyses for All Outcomes**

| **Outcome** | **Subgroup** | **k** | **Pooled Effect (95% CI)** | **P value** | **I² (%)** | **Interaction P** |
| --- | --- | --- | --- | --- | --- | --- |
| **MACE** |  |  |  |  |  |  |
|  | De novo lesions | 8 | 0.98 (0.78–1.23) | 0.87 | 12.3 | 0.89 |
|  | ISR lesions | 4 | 0.82 (0.45–1.48) | 0.51 | 0.0 |  |
|  | Small vessel (<3.0 mm) | 6 | 0.95 (0.72–1.25) | 0.71 | 8.5 | 0.92 |
|  | Non-small vessel | 5 | 0.89 (0.65–1.22) | 0.46 | 15.2 |  |
|  | ACS presentation | 4 | 0.74 (0.26–2.13) | 0.58 | 0.0 | 0.55 |
|  | CCS presentation | 7 | 0.96 (0.75–1.23) | 0.75 | 18.4 |  |
|  | Paclitaxel DCB | 10 | 0.93 (0.74–1.17) | 0.54 | 11.2 | 0.78 |
|  | Sirolimus DCB | 2 | 0.81 (0.34–1.92) | 0.63 | 0.0 |  |
| **TLR** |  |  |  |  |  |  |
|  | De novo lesions | 7 | 1.76 (1.03–3.02) | 0.039 | 56.9 | 0.074 |
|  | ISR lesions | 4 | 3.54 (2.05–6.09) | <0.001 | 0.0 |  |
|  | Small vessel (<3.0 mm) | 5 | 1.17 (0.64–2.14) | 0.604 | 15.1 | 0.12 |
|  | Non-small vessel | 6 | 2.85 (1.89–4.30) | <0.001 | 38.5 |  |
|  | BMS-ISR | 2 | 3.16 (1.29–7.70) | 0.012 | 0.0 | 0.45 |
|  | DES-ISR | 3 | 3.87 (1.66–9.02) | 0.002 | 0.0 |  |
|  | Diabetes present | 4 | 0.82 (0.45–1.48) | 0.51 | 0.0 | 0.011* |
|  | Diabetes absent | 7 | 1.64 (0.83–3.25) | 0.16 | 42.3 |  |
| **Cardiac Death** |  |  |  |  |  |  |
|  | De novo lesions | 6 | 1.62 (1.15–2.28) | 0.006 | 0.0 | 0.34 |
|  | ISR lesions | 3 | 1.12 (0.38–3.31) | 0.83 | 0.0 |  |
|  | Small vessel | 4 | 1.28 (0.63–2.66) | 0.49 | 0.0 | 0.52 |
|  | Non-small vessel | 4 | 1.71 (1.11–2.63) | 0.015 | 0.0 |  |
| **DoCE** |  |  |  |  |  |  |
|  | CKD patients | 2 | HR 1.57 (0.66–3.71) | 0.32 | 0.0 | 0.35 |
|  | Non-CKD patients | 2 | HR 2.27 (1.38–3.72) | 0.002 | 0.0 |  |
|  | LAD proximal | 1 | HR 2.68 (1.34–5.35) | 0.008 | — | 0.26 |
|  | Non-LAD proximal | 1 | HR 1.72 (1.04–2.85) | 0.038 | — |  |

*Note.* *Significant interaction for TLR: diabetes status modifies treatment effect (DCB more favorable in diabetes). CCS = chronic coronary syndrome;
