# Supplementary figures and images for: Drug-coated balloons vs. drug-eluting stents for coronary artery disease: an updated systematic review and meta-analysis of randomized controlled trials with lesion-specific insights
Source: Front Cardiovasc Med. 2026 May 18;13:1843262. doi: 10.3389/fcvm.2026.1843262 (PMC13223156; doi:10.3389/fcvm.2026.1843262)

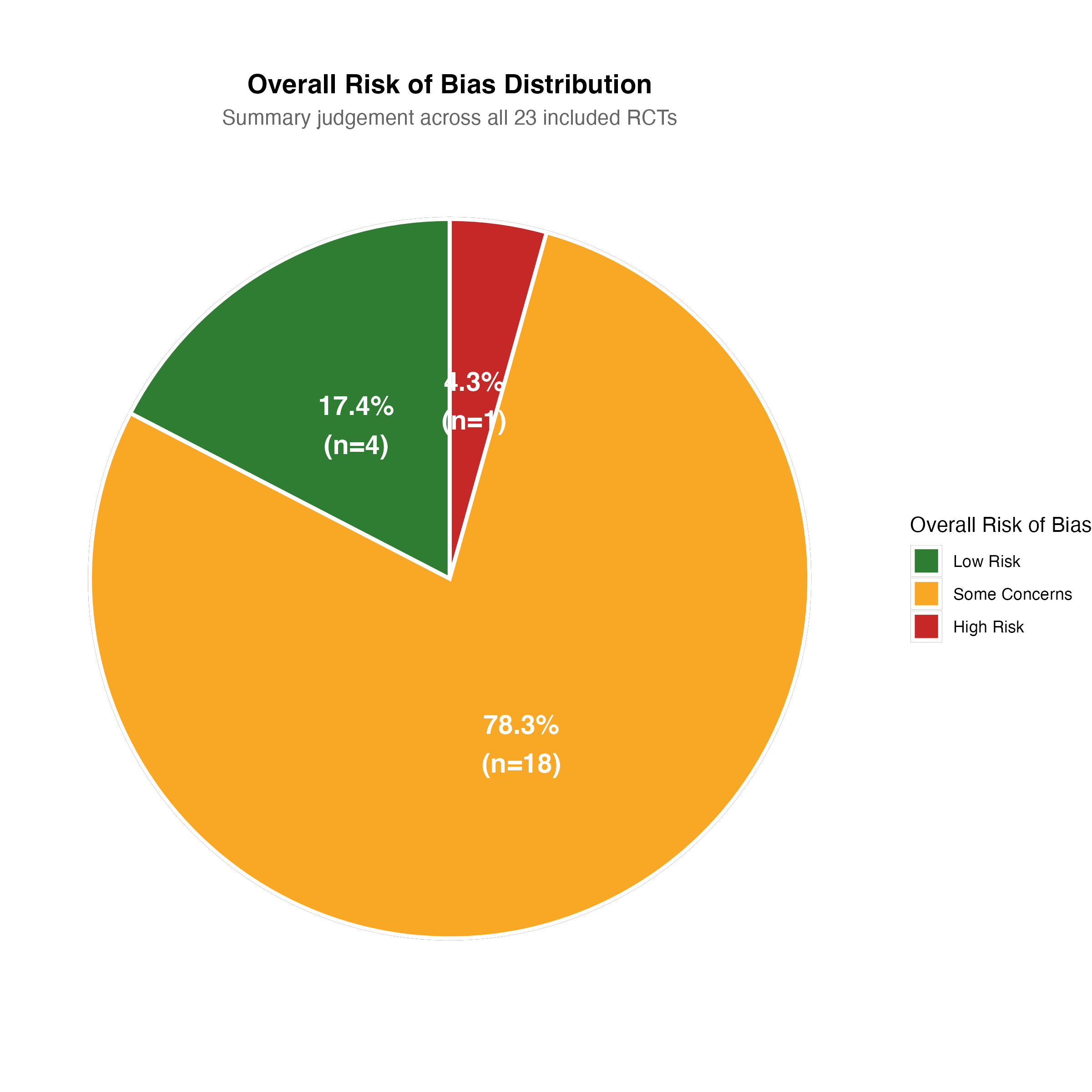

Supplement: Supplementary file 5 [file Datasheet1.zip › R scripts/3.tiff]

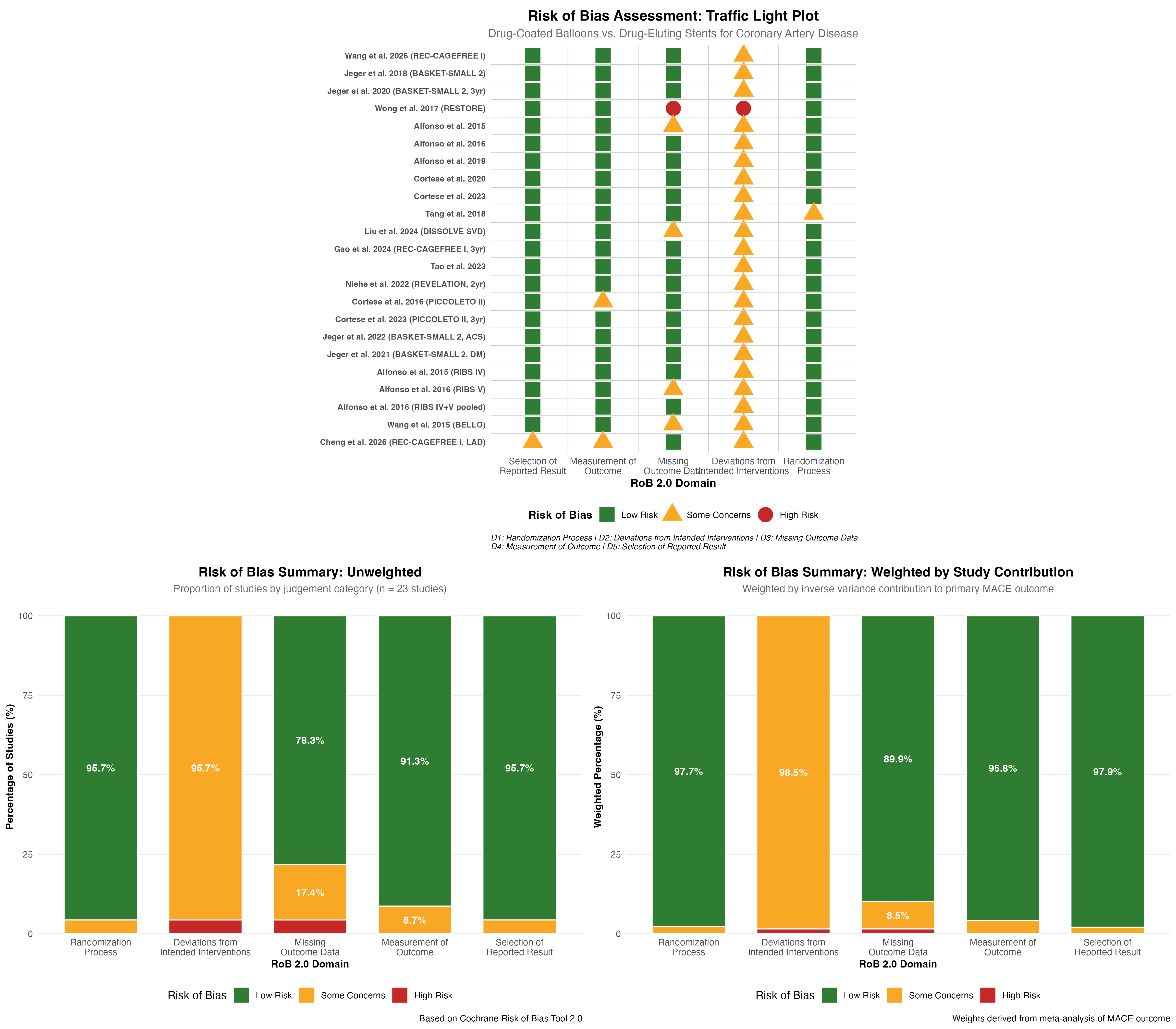

Supplement: Supplementary file 5 [file Datasheet1.zip › R scripts/2.tiff]

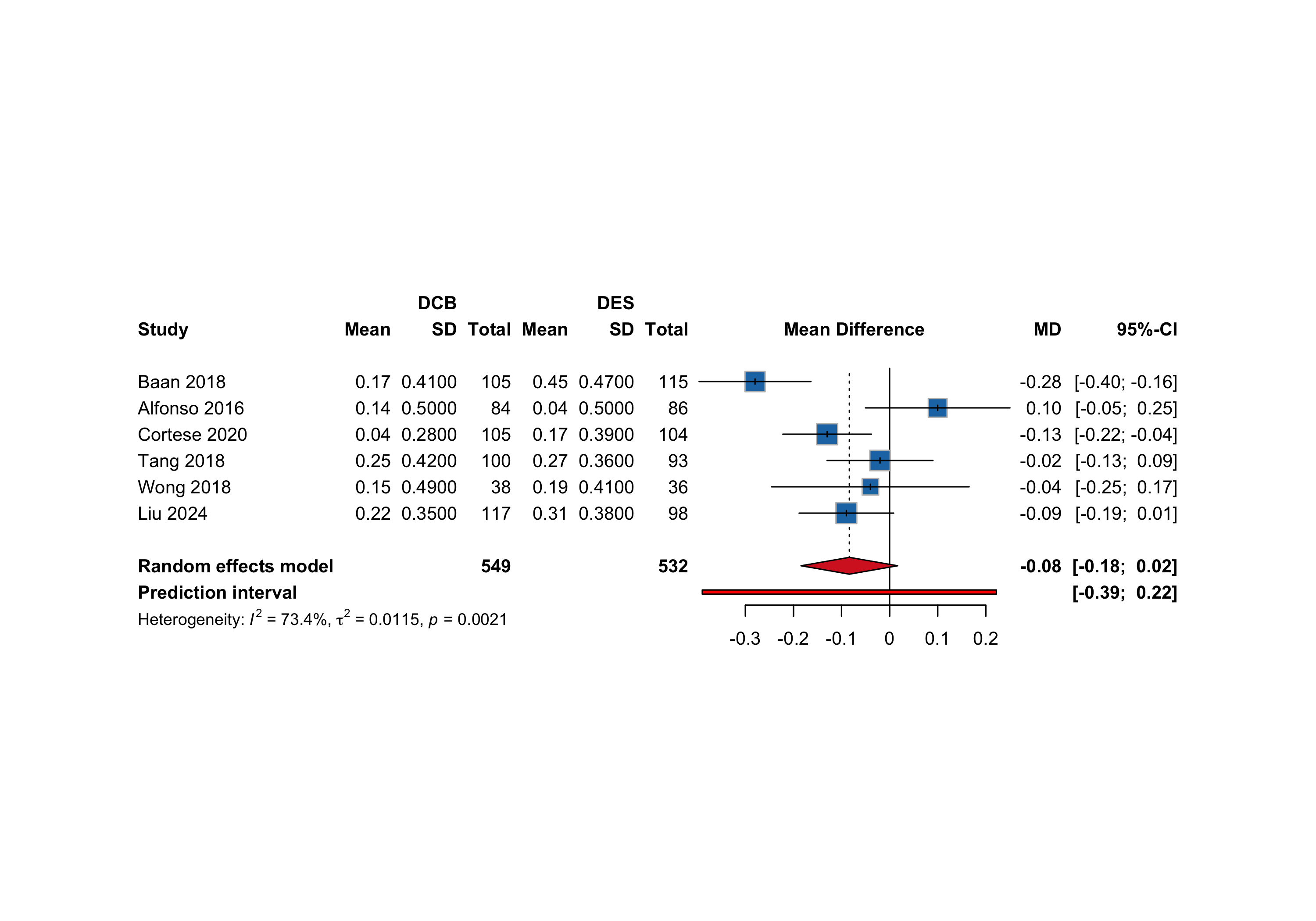

Supplement: Supplementary file 5 [file Datasheet1.zip › R scripts/13-S1/Late_lumen_loss_forest.tiff]

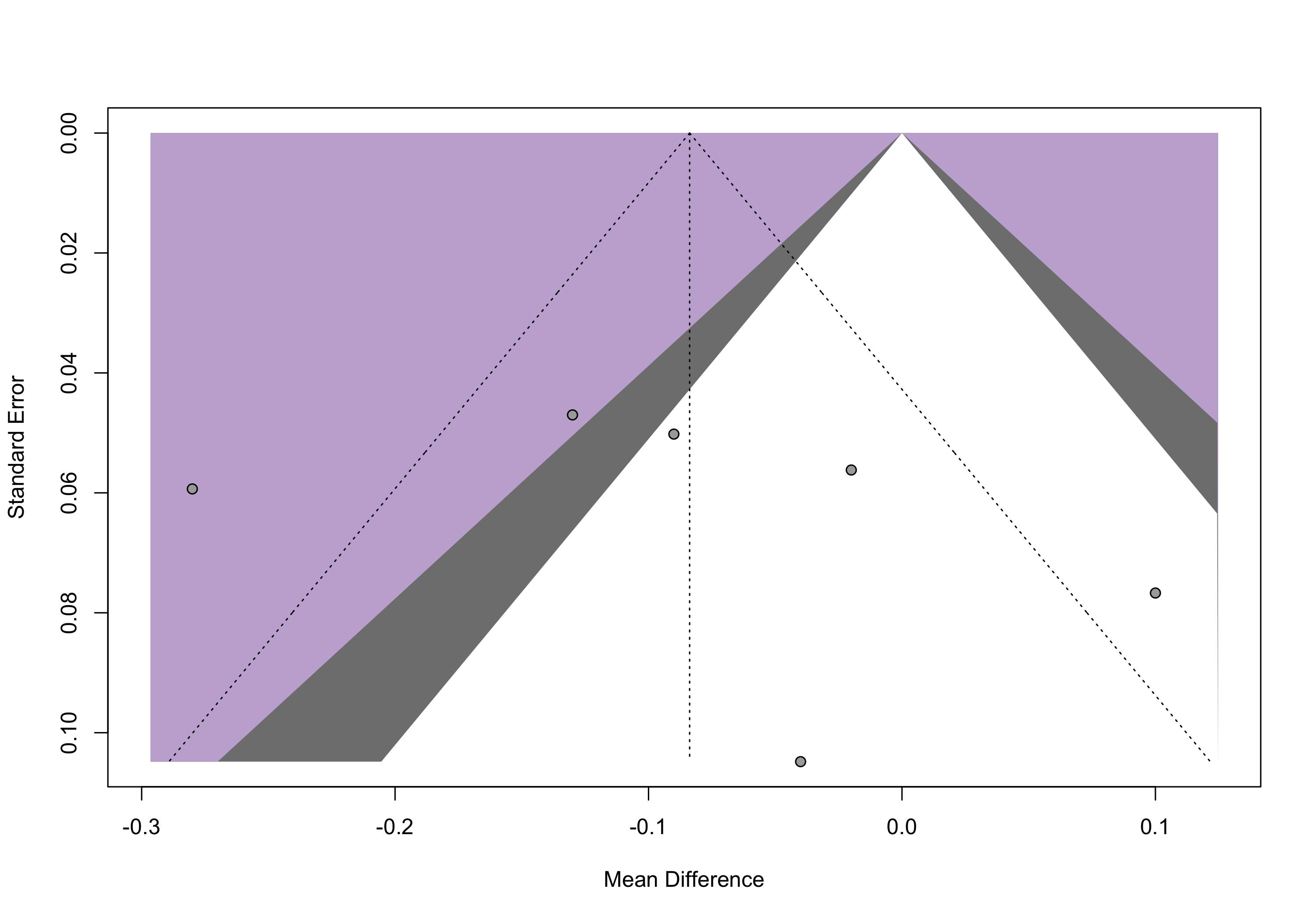

Supplement: Supplementary file 5 [file Datasheet1.zip › R scripts/13-S1/Late_lumen_loss_funnel.tiff]

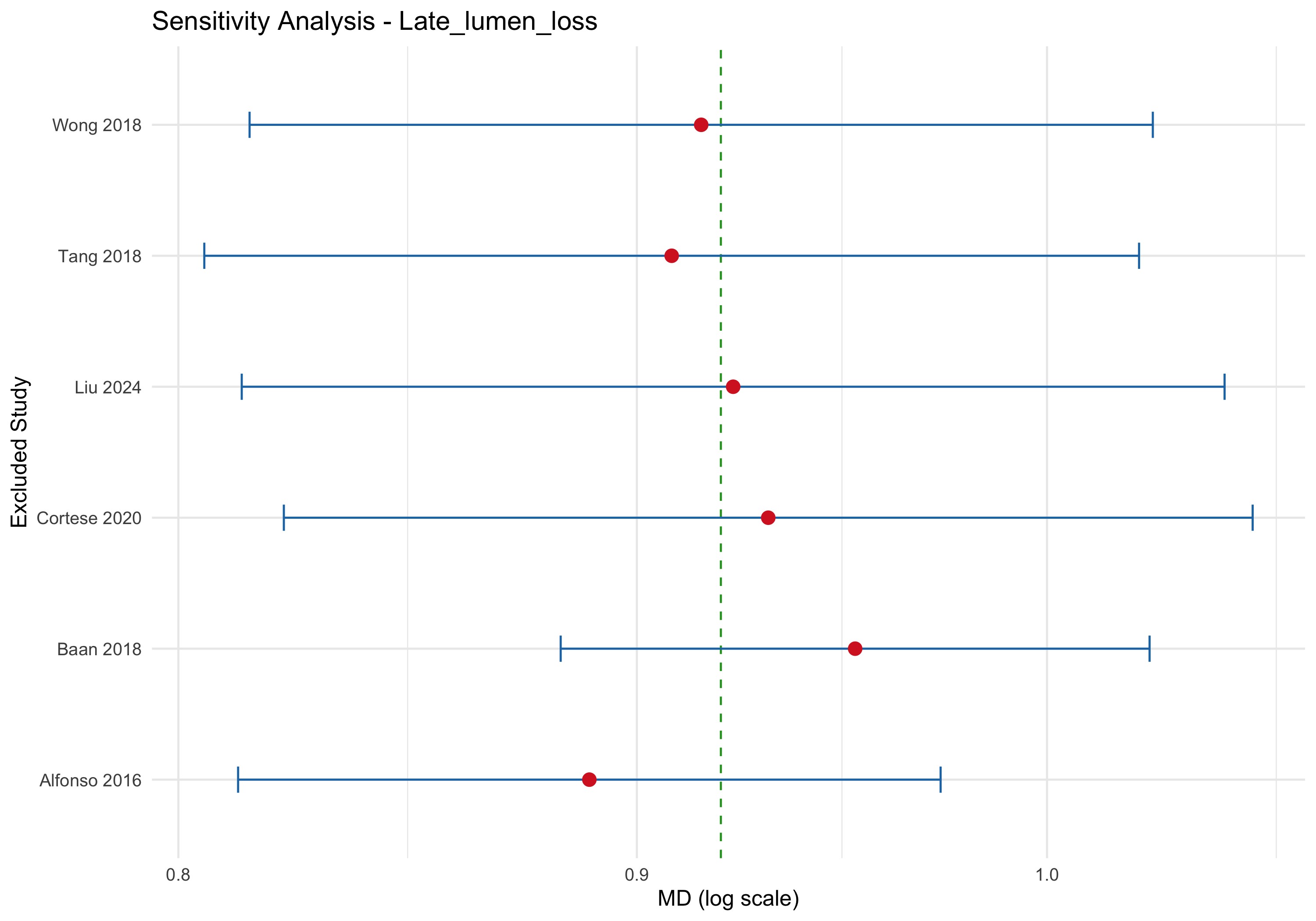

Supplement: Supplementary file 5 [file Datasheet1.zip › R scripts/13-S1/Late_lumen_loss_sensitivity.tiff]

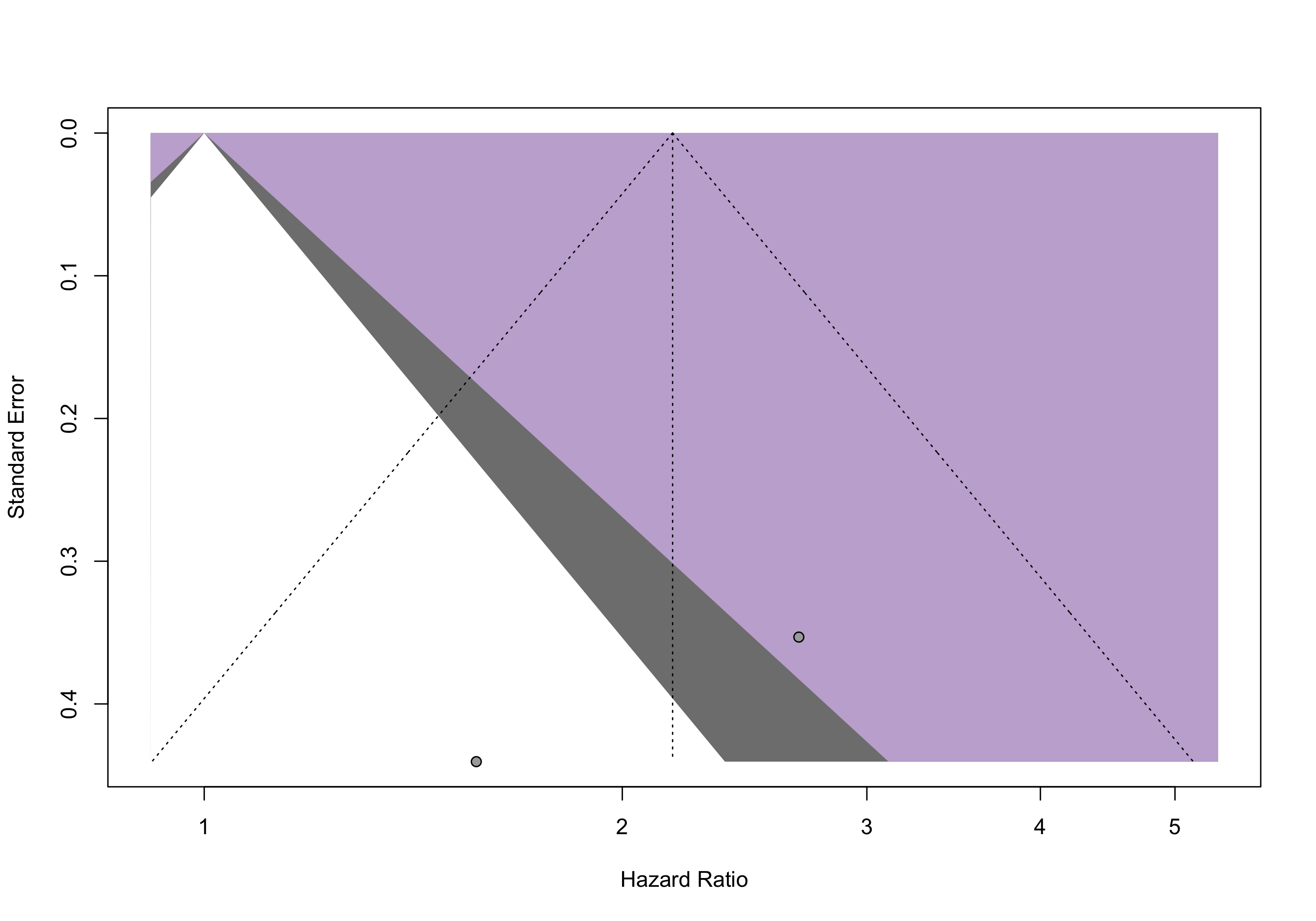

Supplement: Supplementary file 5 [file Datasheet1.zip › R scripts/15-S3/DoCE_HR_funnel.tiff]

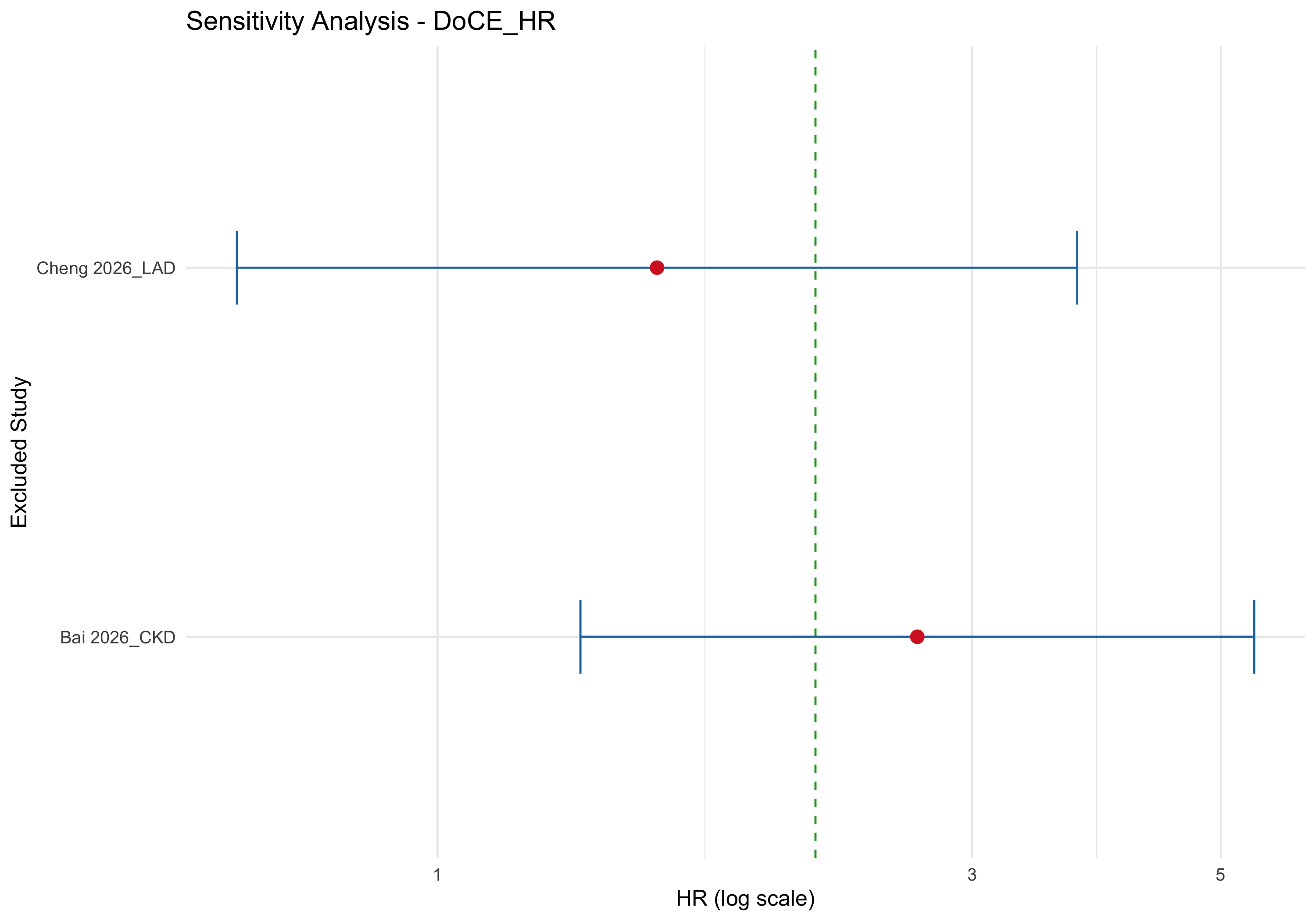

Supplement: Supplementary file 5 [file Datasheet1.zip › R scripts/15-S3/DoCE_HR_sensitivity.tiff]

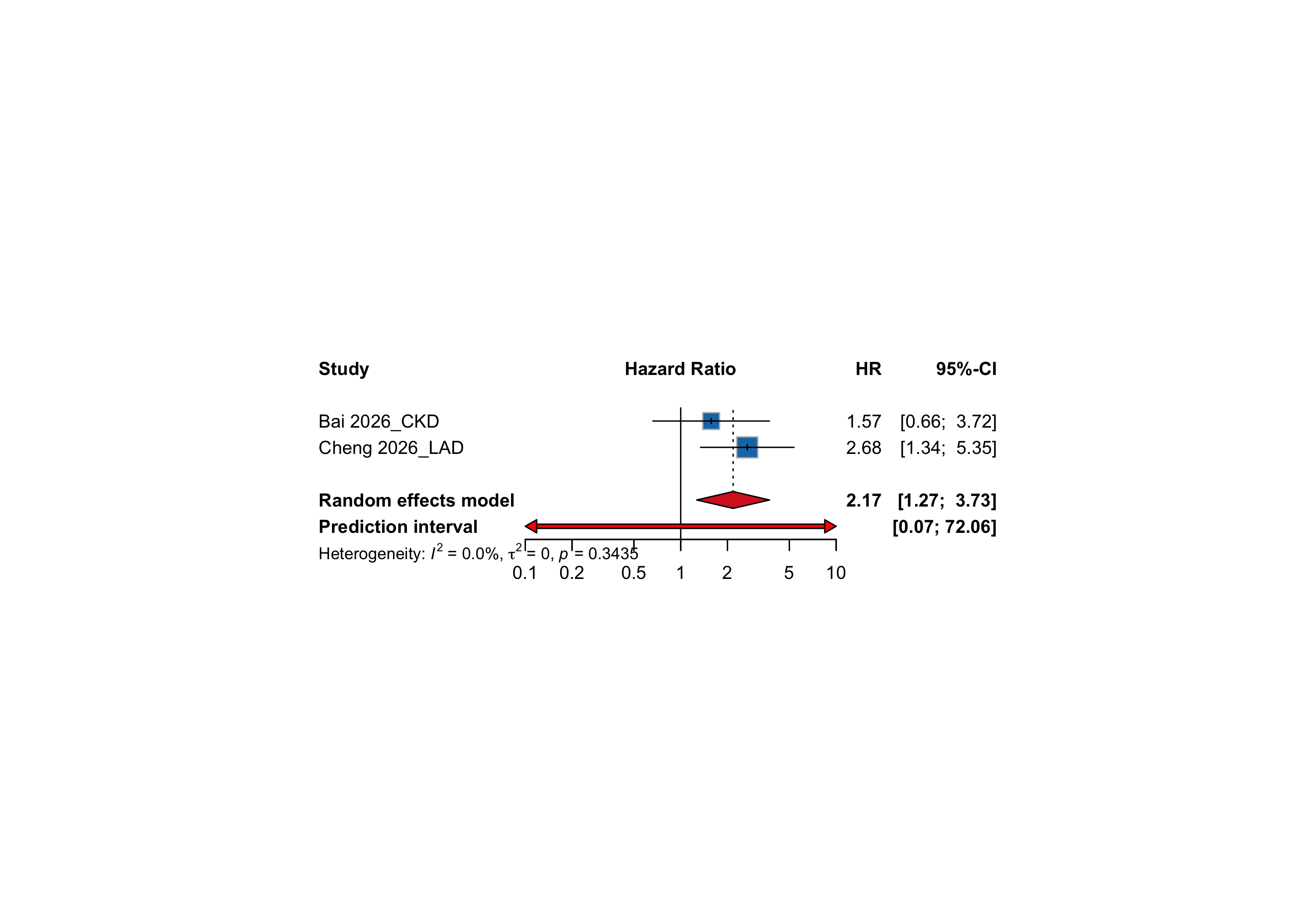

Supplement: Supplementary file 5 [file Datasheet1.zip › R scripts/15-S3/DoCE_HR_forest.tiff]

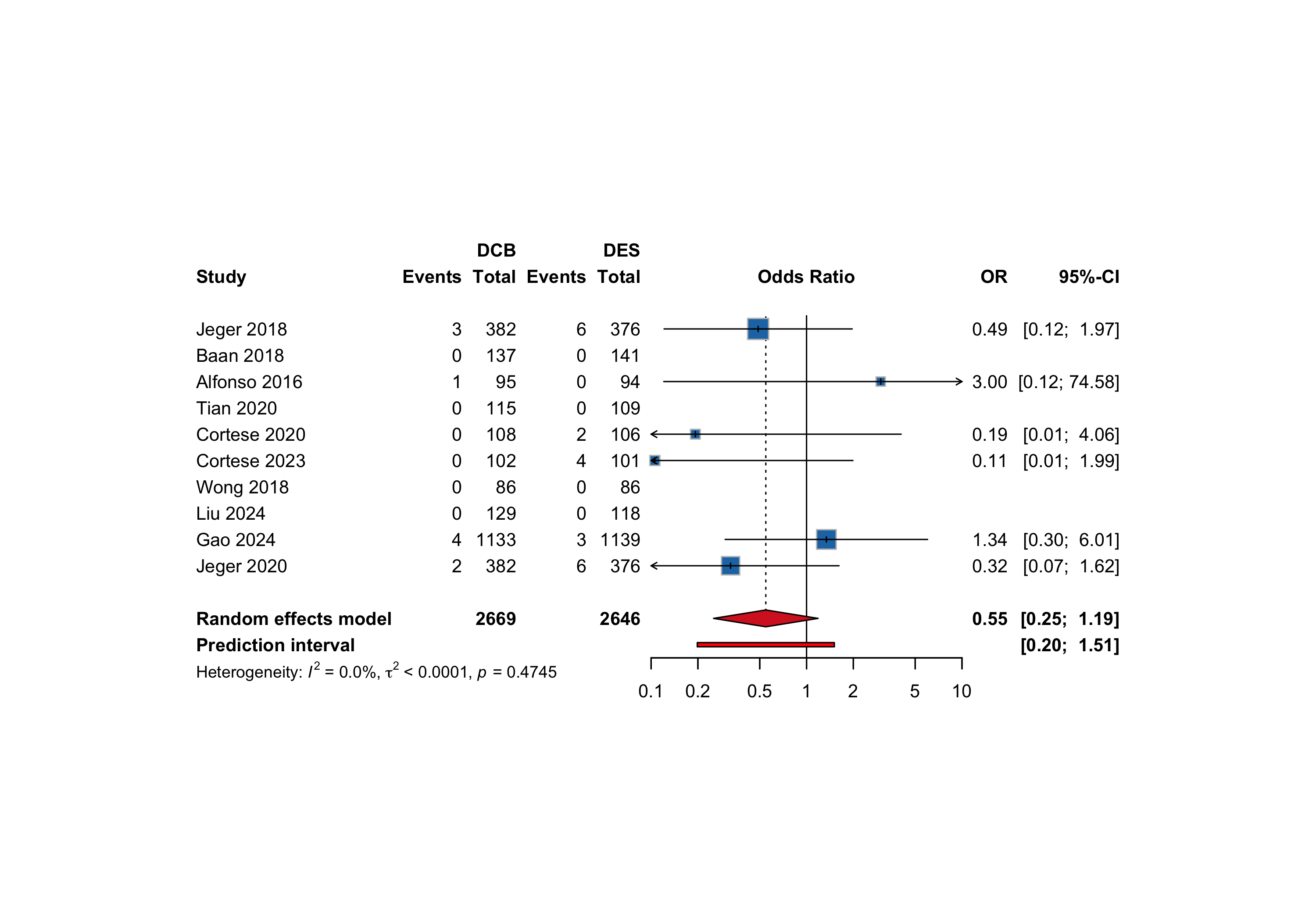

Supplement: Supplementary file 5 [file Datasheet1.zip › R scripts/9/Stent_thrombosis_forest.tiff]

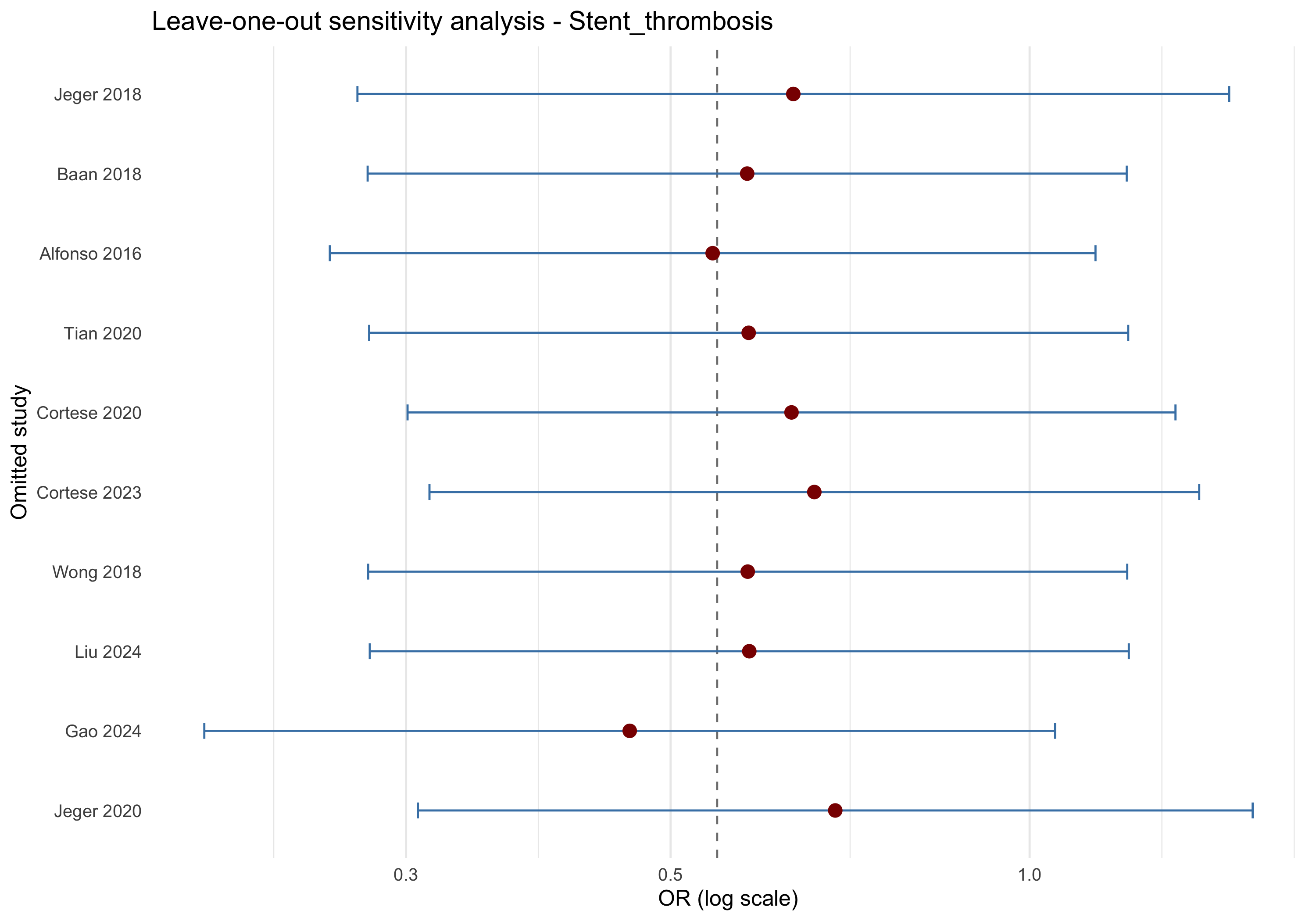

Supplement: Supplementary file 5 [file Datasheet1.zip › R scripts/9/Stent_thrombosis_sensitivity_leave1out.tiff]

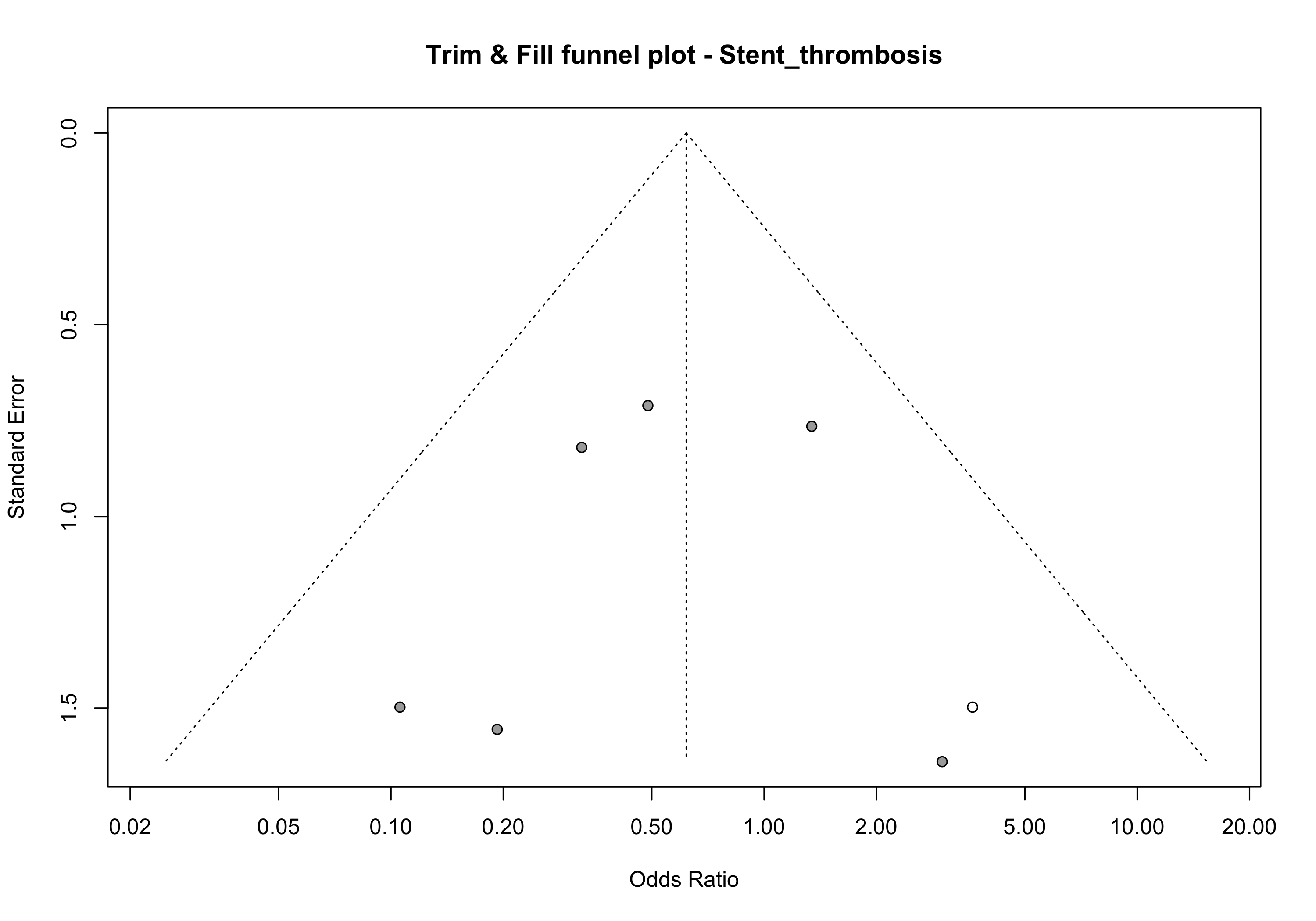

Supplement: Supplementary file 5 [file Datasheet1.zip › R scripts/9/Stent_thrombosis_trimfill_funnel.tiff]

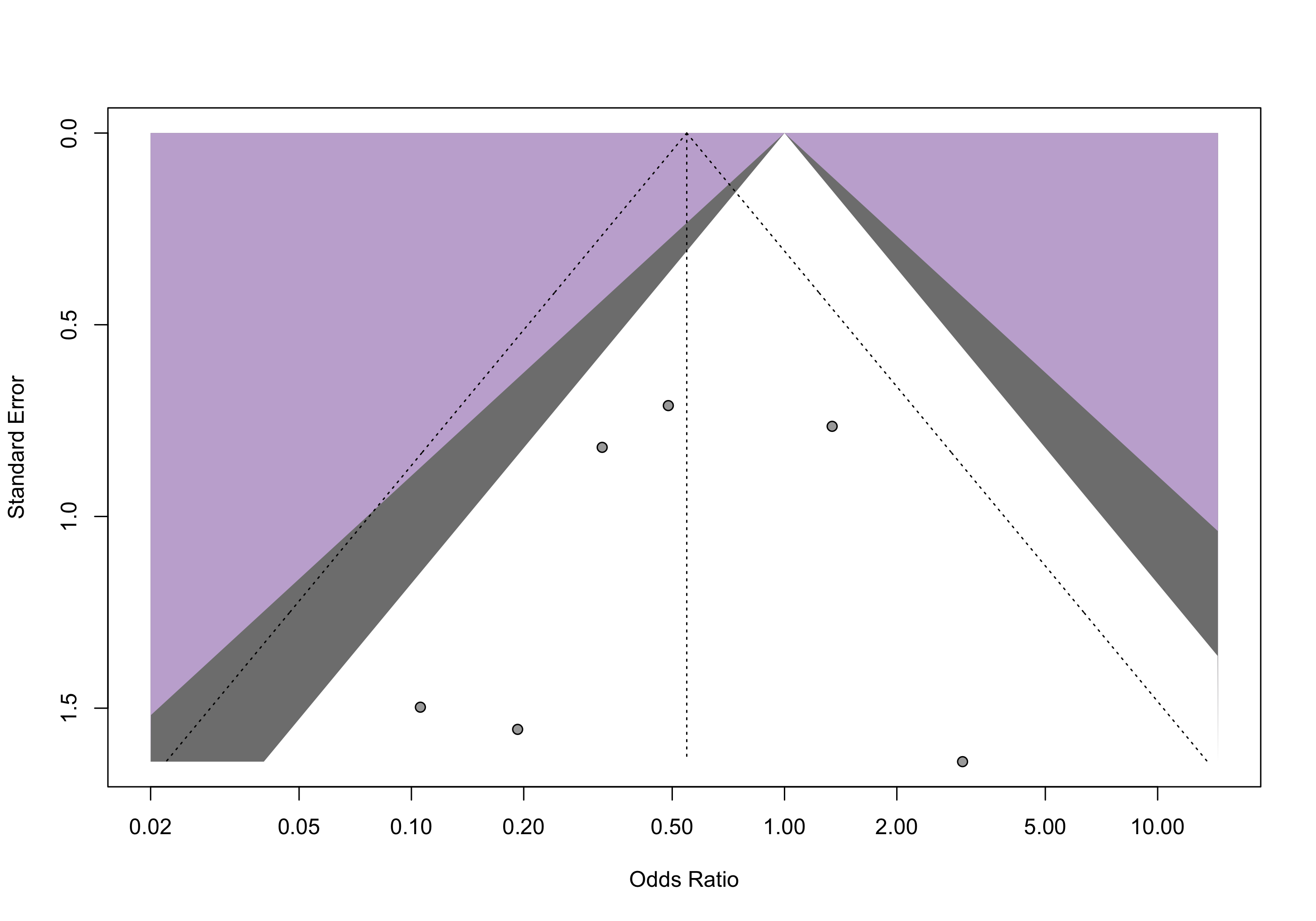

Supplement: Supplementary file 5 [file Datasheet1.zip › R scripts/9/Stent_thrombosis_funnel.tiff]

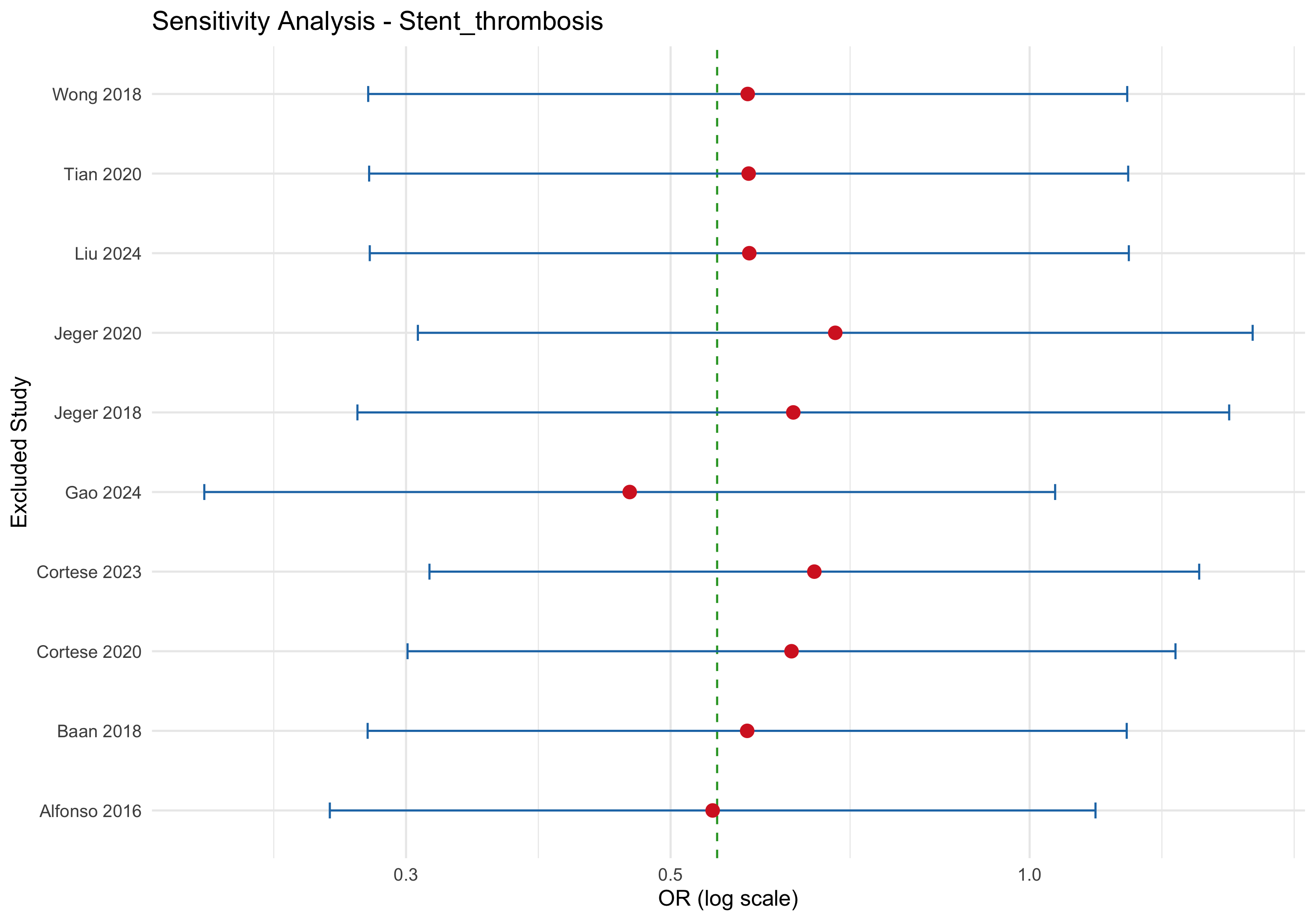

Supplement: Supplementary file 5 [file Datasheet1.zip › R scripts/9/Stent_thrombosis_sensitivity.tiff]

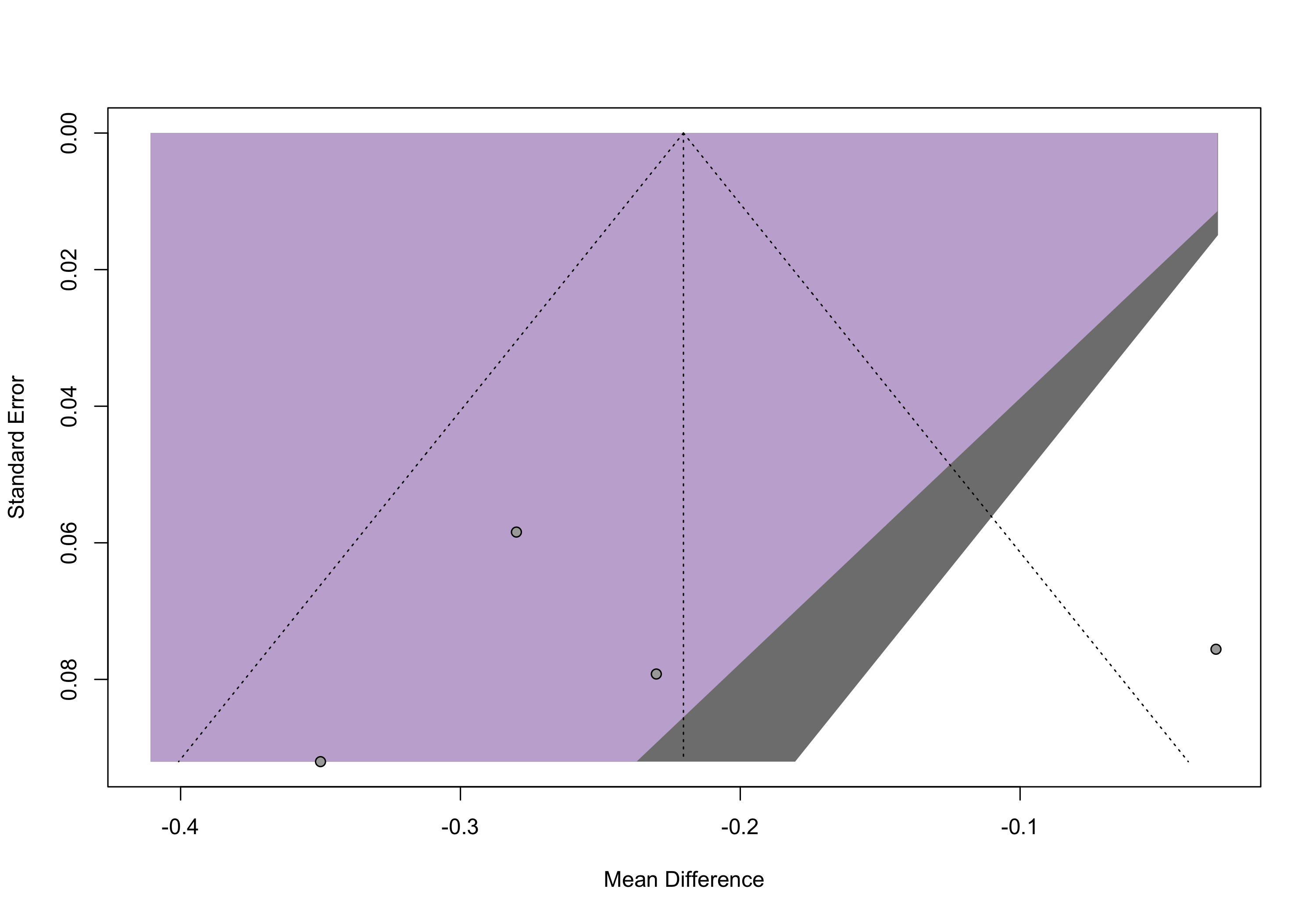

Supplement: Supplementary file 5 [file Datasheet1.zip › R scripts/14-S2/Minimal_lumen_diameter_funnel.tiff]

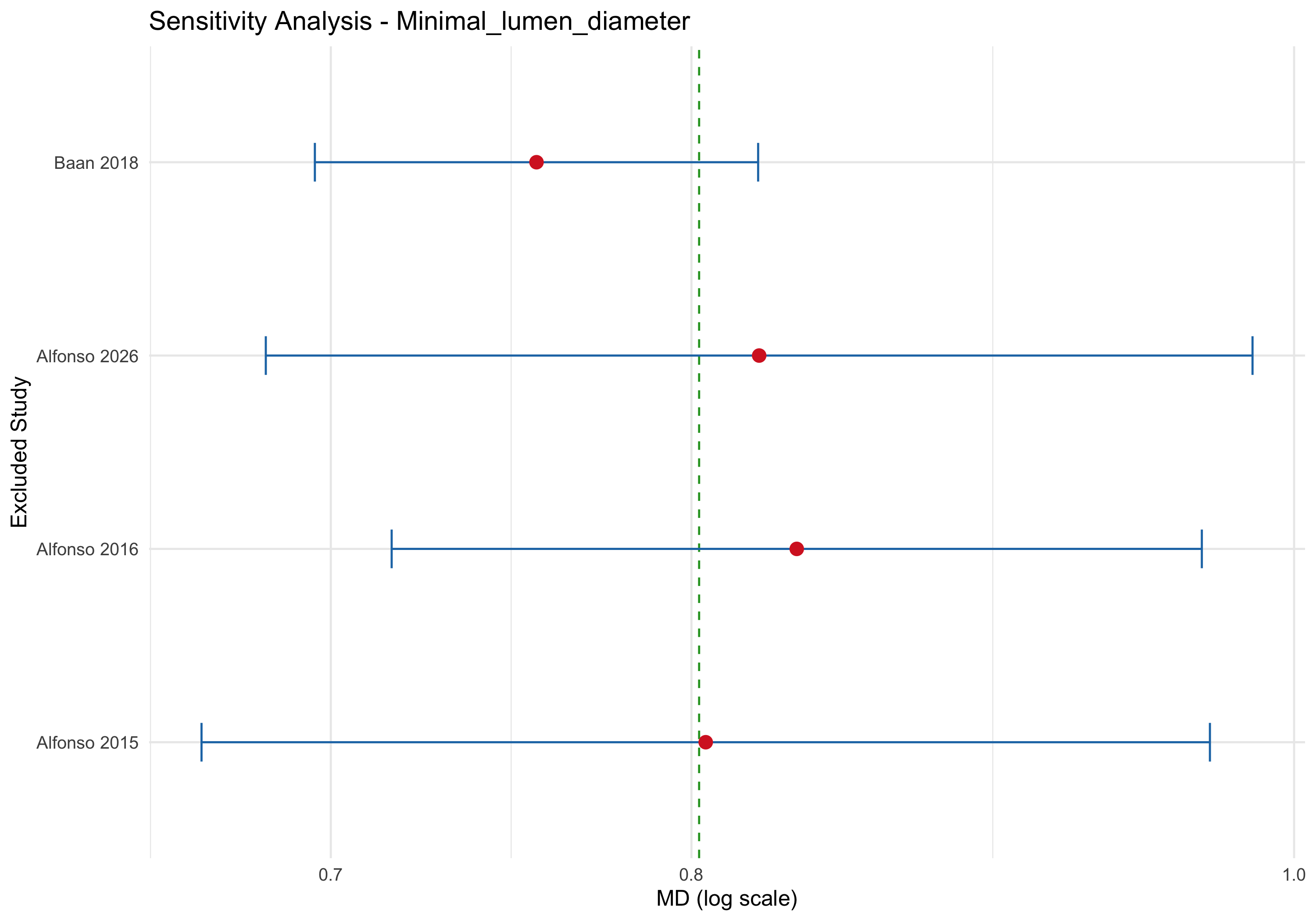

Supplement: Supplementary file 5 [file Datasheet1.zip › R scripts/14-S2/Minimal_lumen_diameter_sensitivity.tiff]

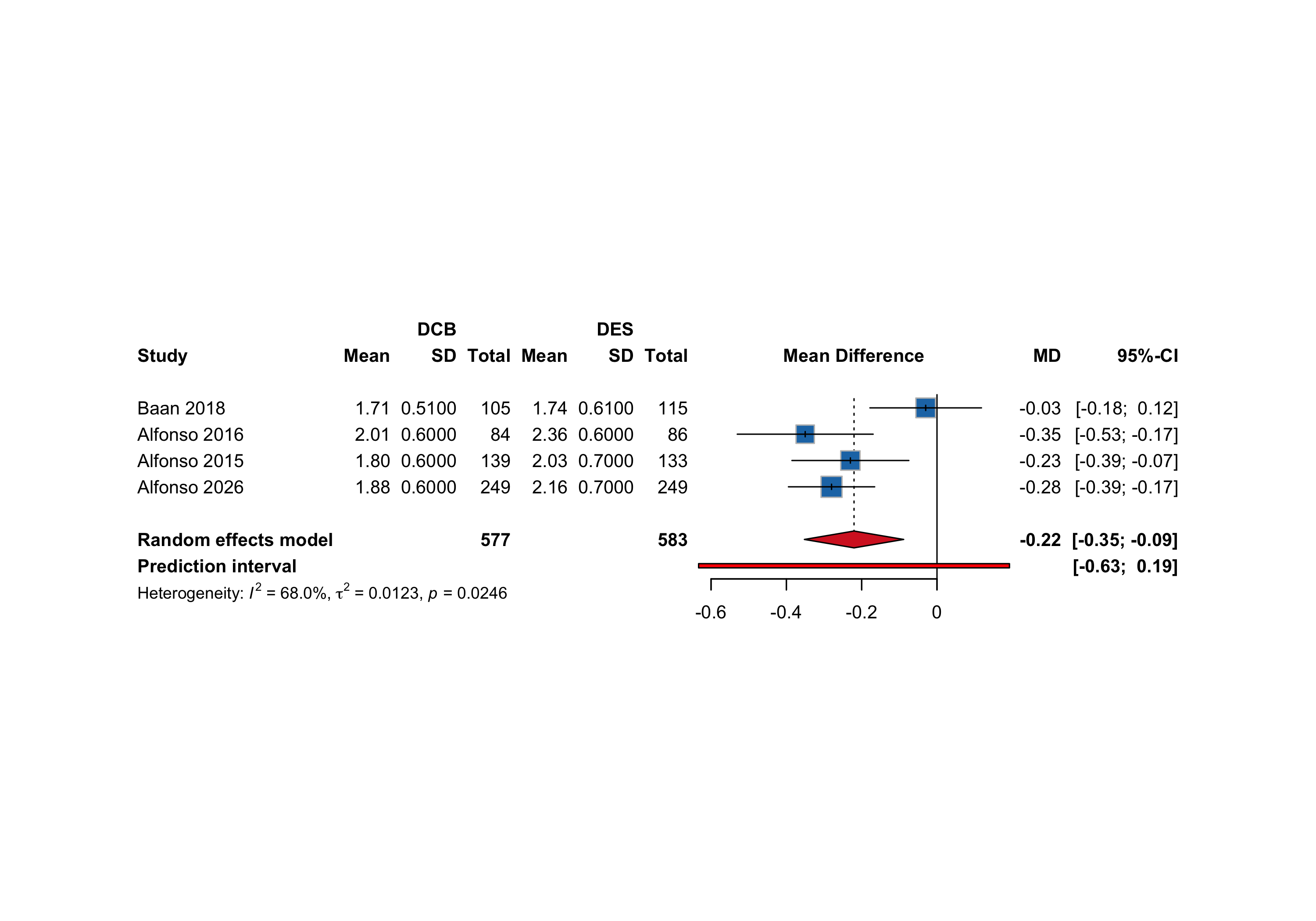

Supplement: Supplementary file 5 [file Datasheet1.zip › R scripts/14-S2/Minimal_lumen_diameter_forest.tiff]

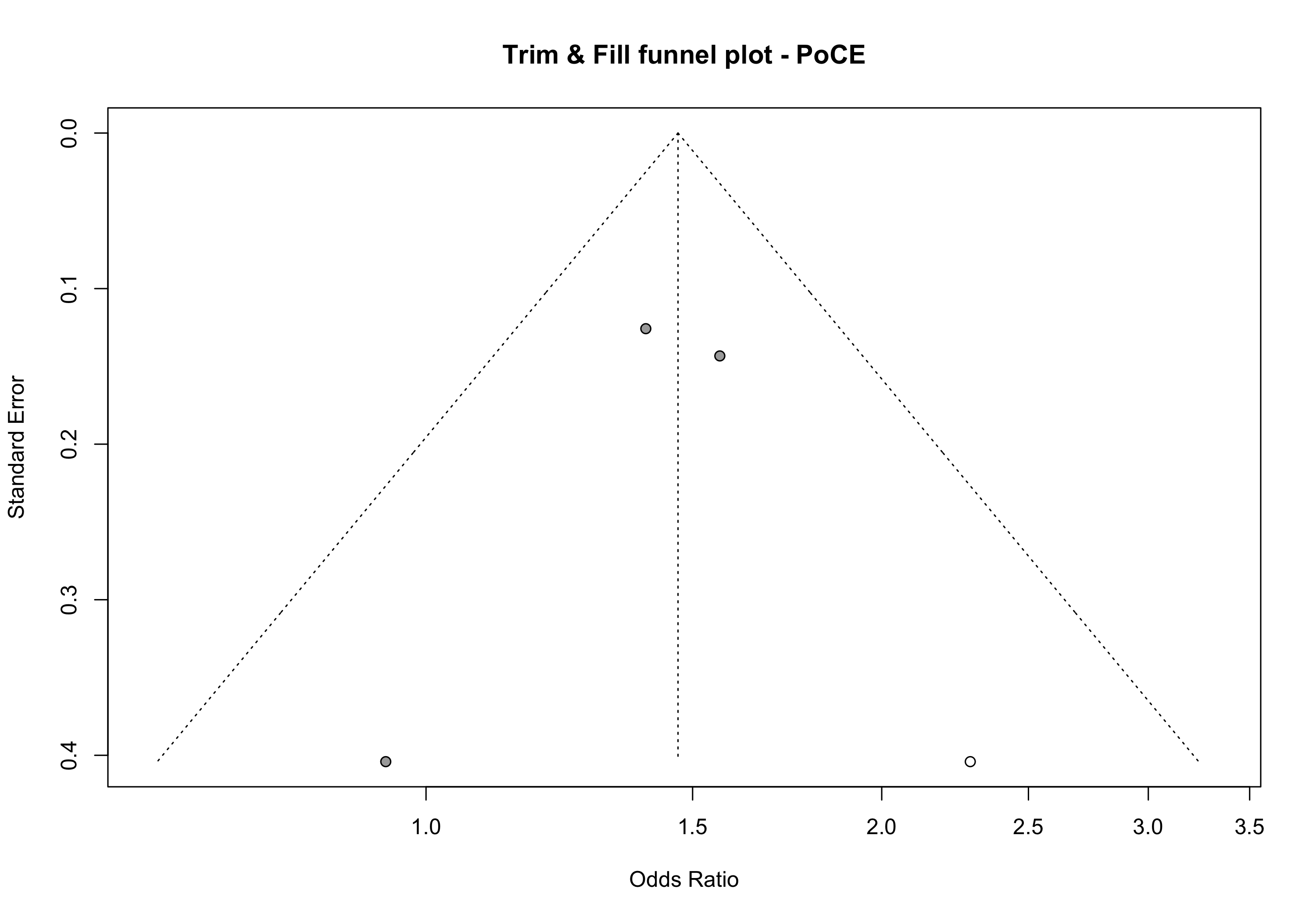

Supplement: Supplementary file 5 [file Datasheet1.zip › R scripts/11/PoCE_trimfill_funnel.tiff]

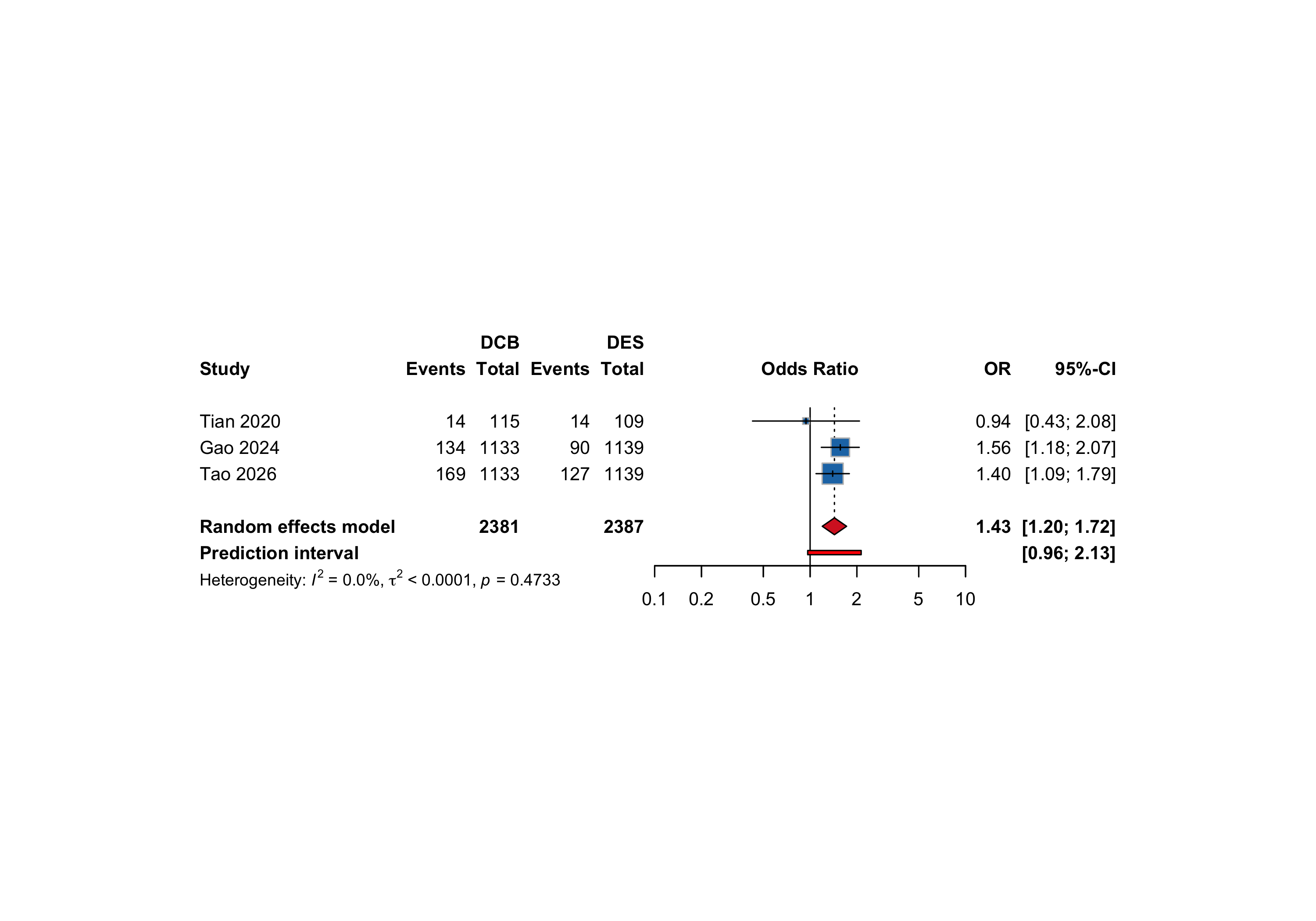

Supplement: Supplementary file 5 [file Datasheet1.zip › R scripts/11/PoCE_forest.tiff]

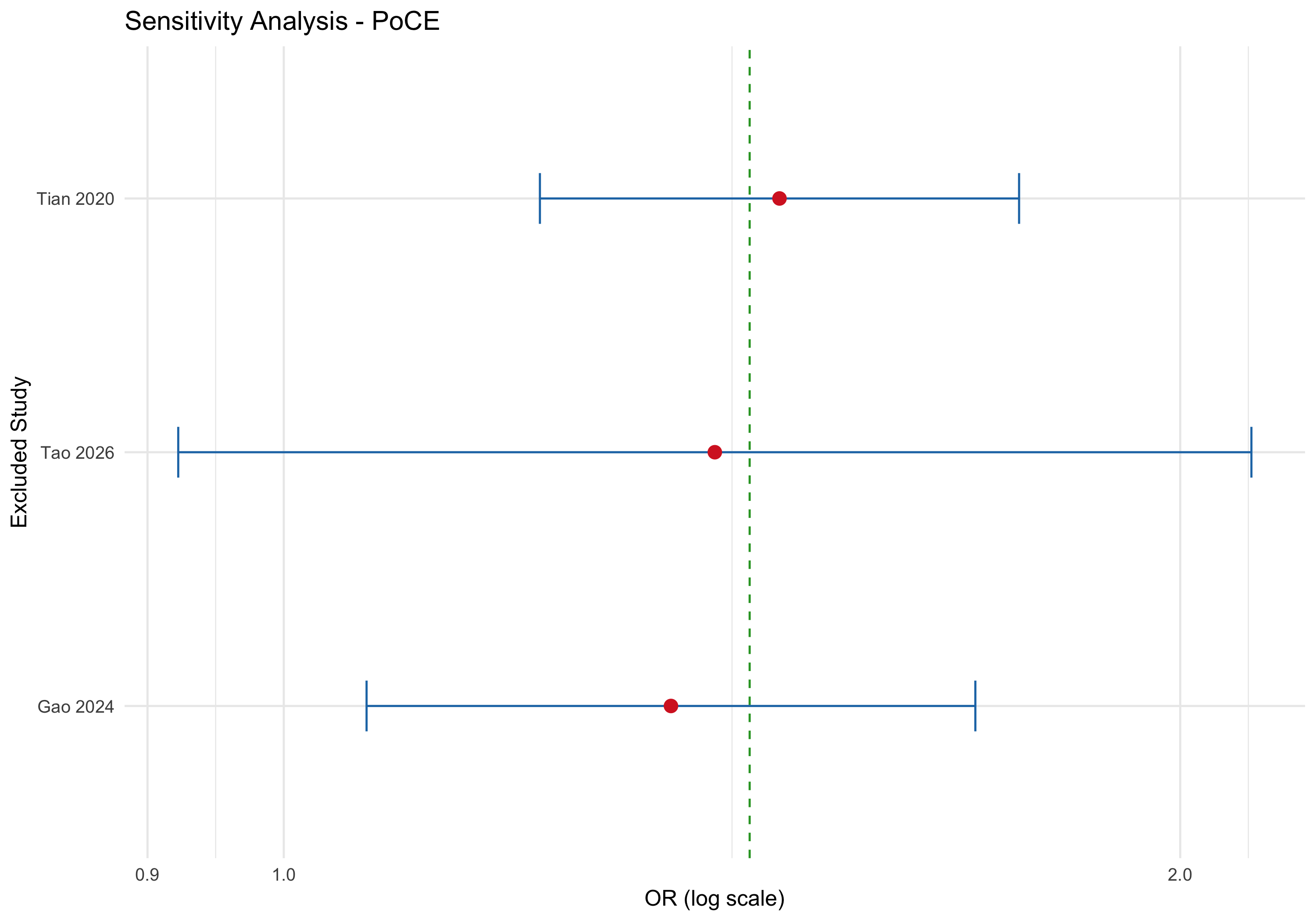

Supplement: Supplementary file 5 [file Datasheet1.zip › R scripts/11/PoCE_sensitivity.tiff]

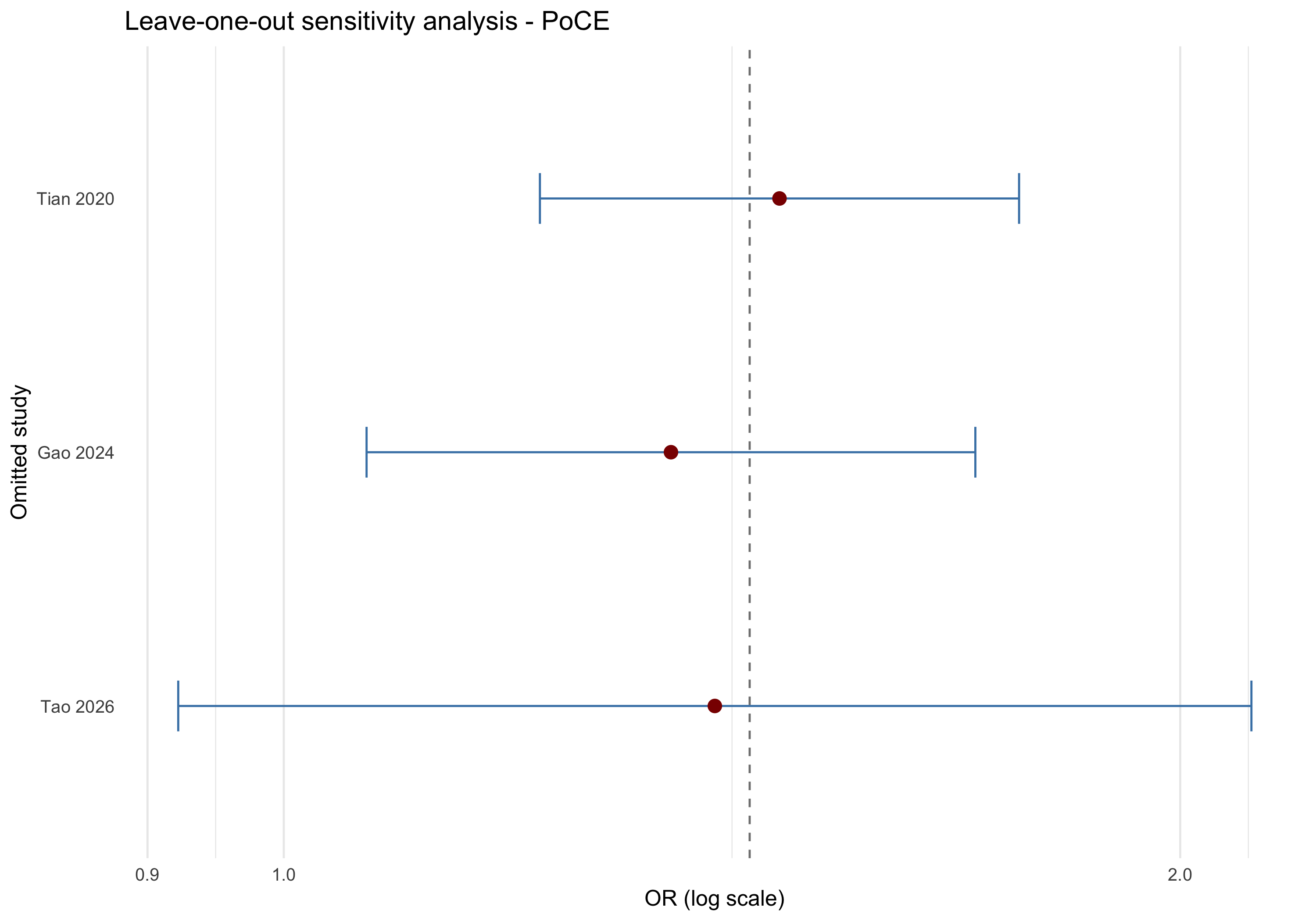

Supplement: Supplementary file 5 [file Datasheet1.zip › R scripts/11/PoCE_sensitivity_leave1out.tiff]

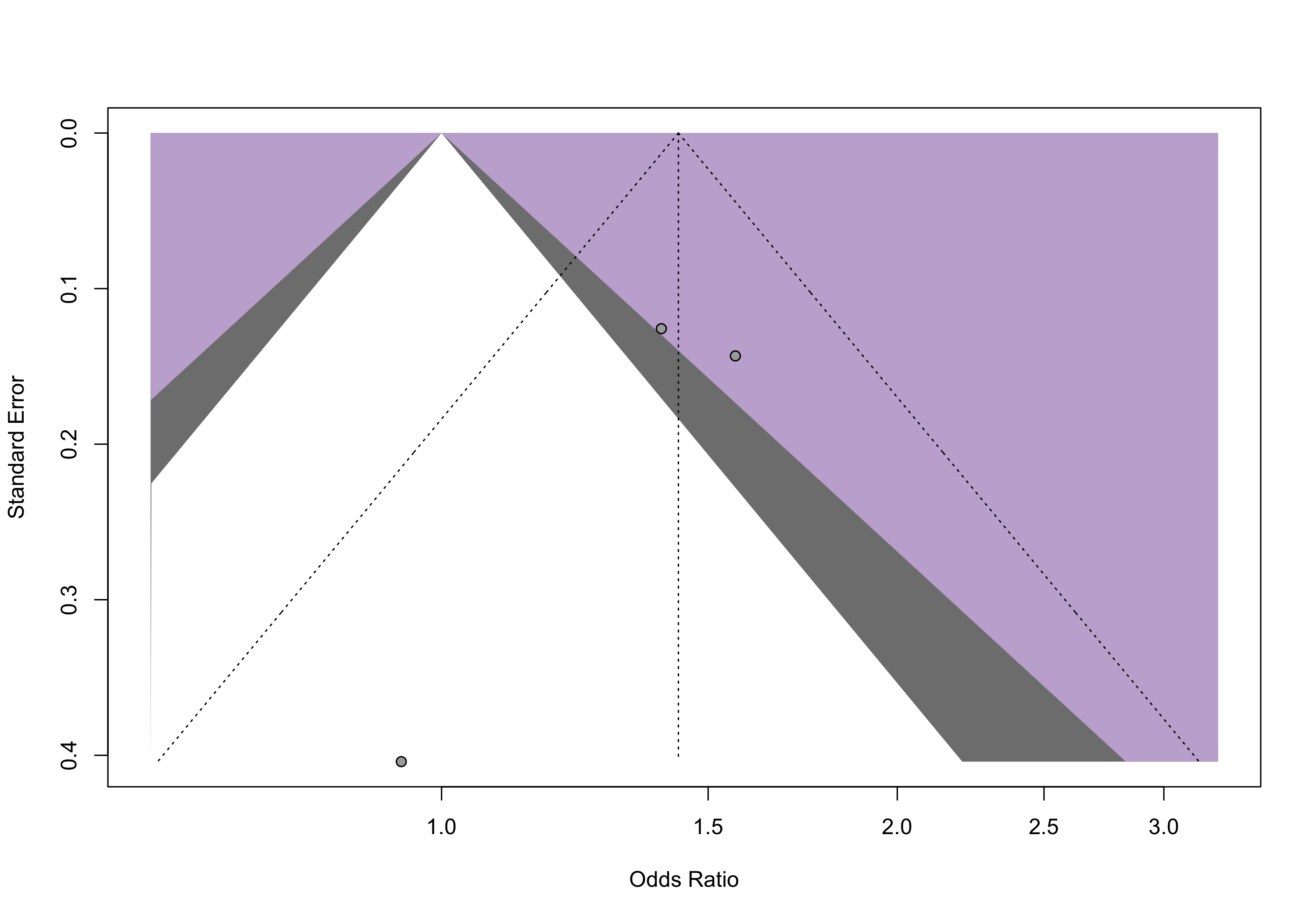

Supplement: Supplementary file 5 [file Datasheet1.zip › R scripts/11/PoCE_funnel.tiff]

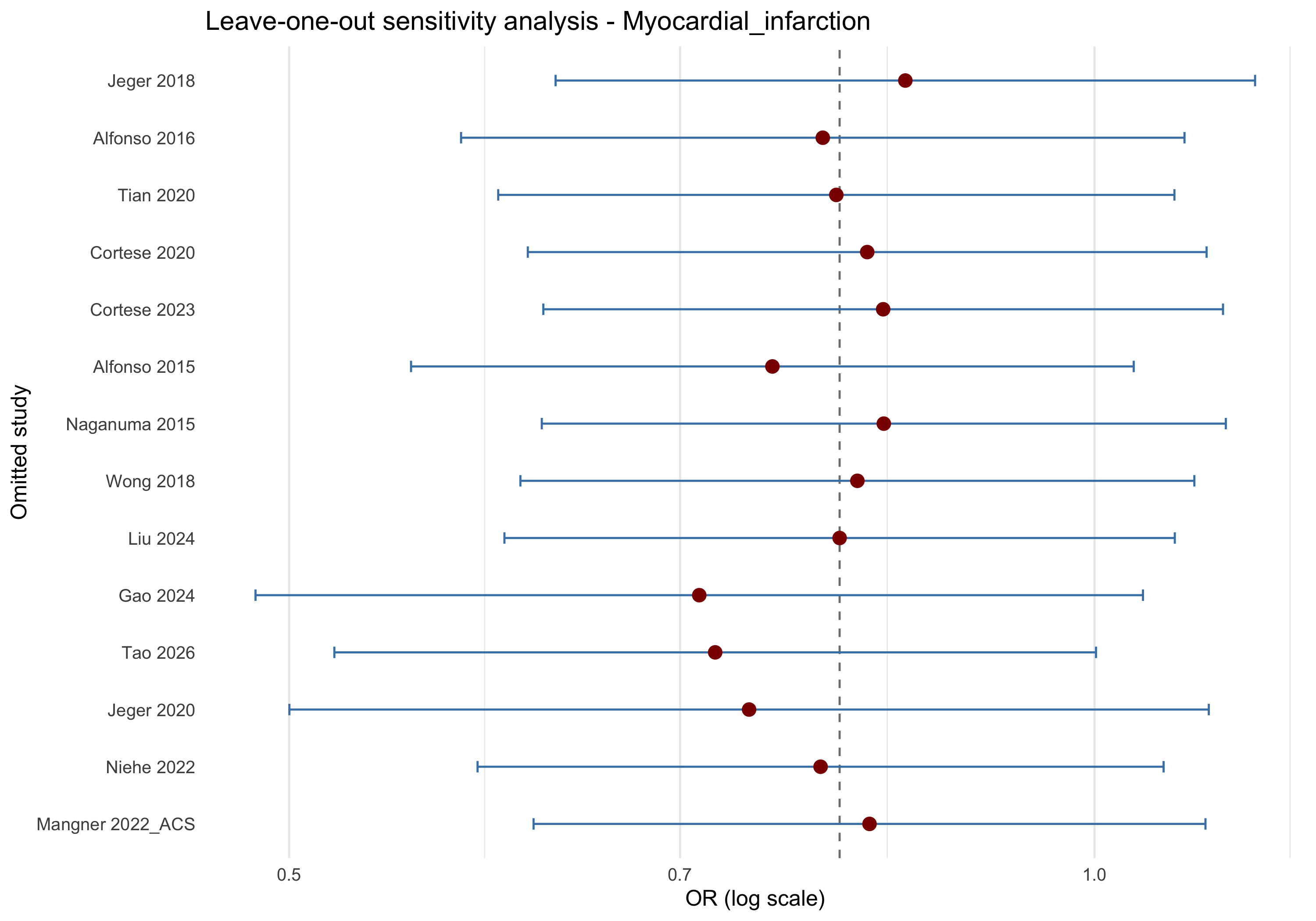

Supplement: Supplementary file 5 [file Datasheet1.zip › R scripts/7/Myocardial_infarction_sensitivity_leave1out.tiff]

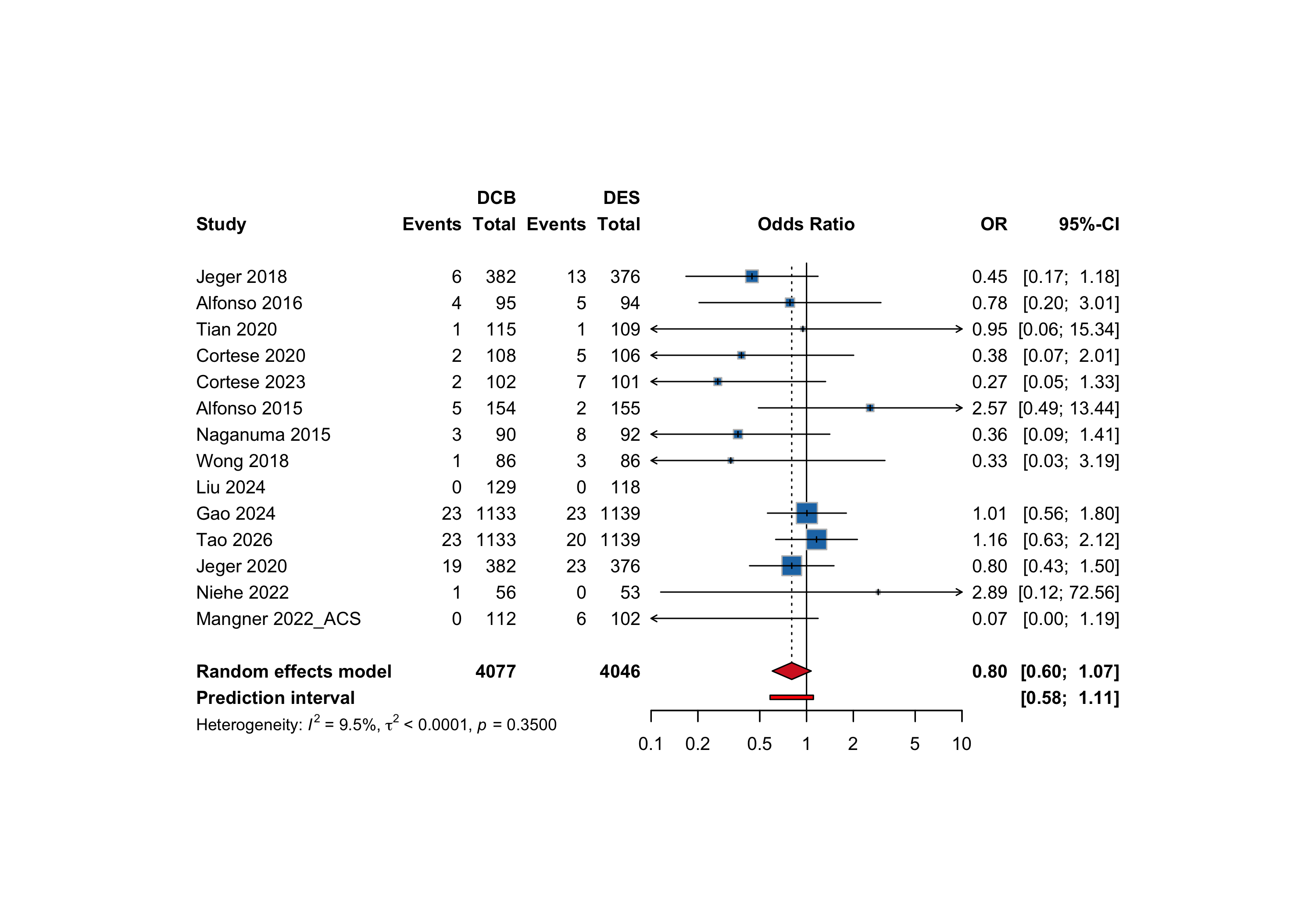

Supplement: Supplementary file 5 [file Datasheet1.zip › R scripts/7/Myocardial_infarction_forest.tiff]

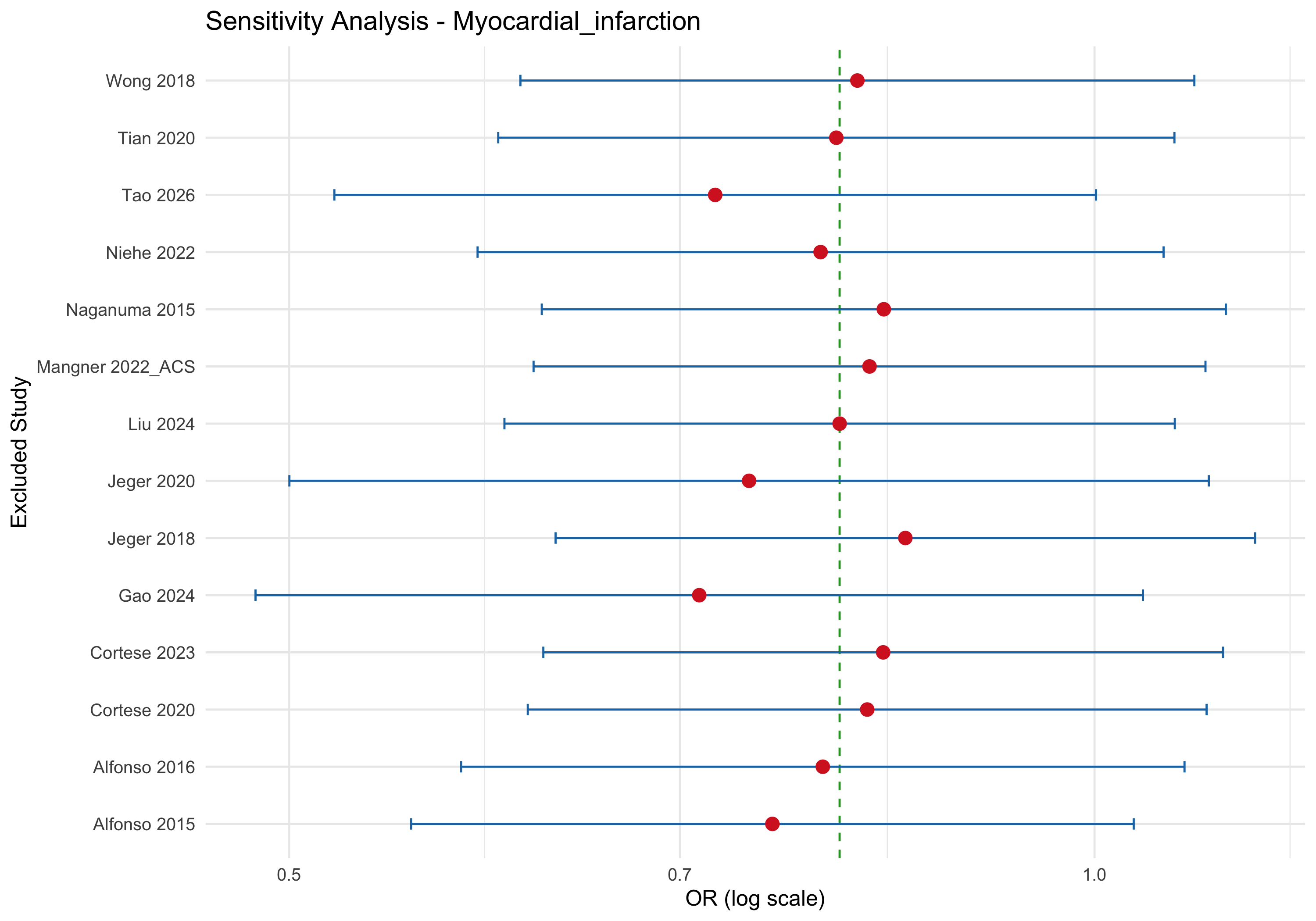

Supplement: Supplementary file 5 [file Datasheet1.zip › R scripts/7/Myocardial_infarction_sensitivity.tiff]

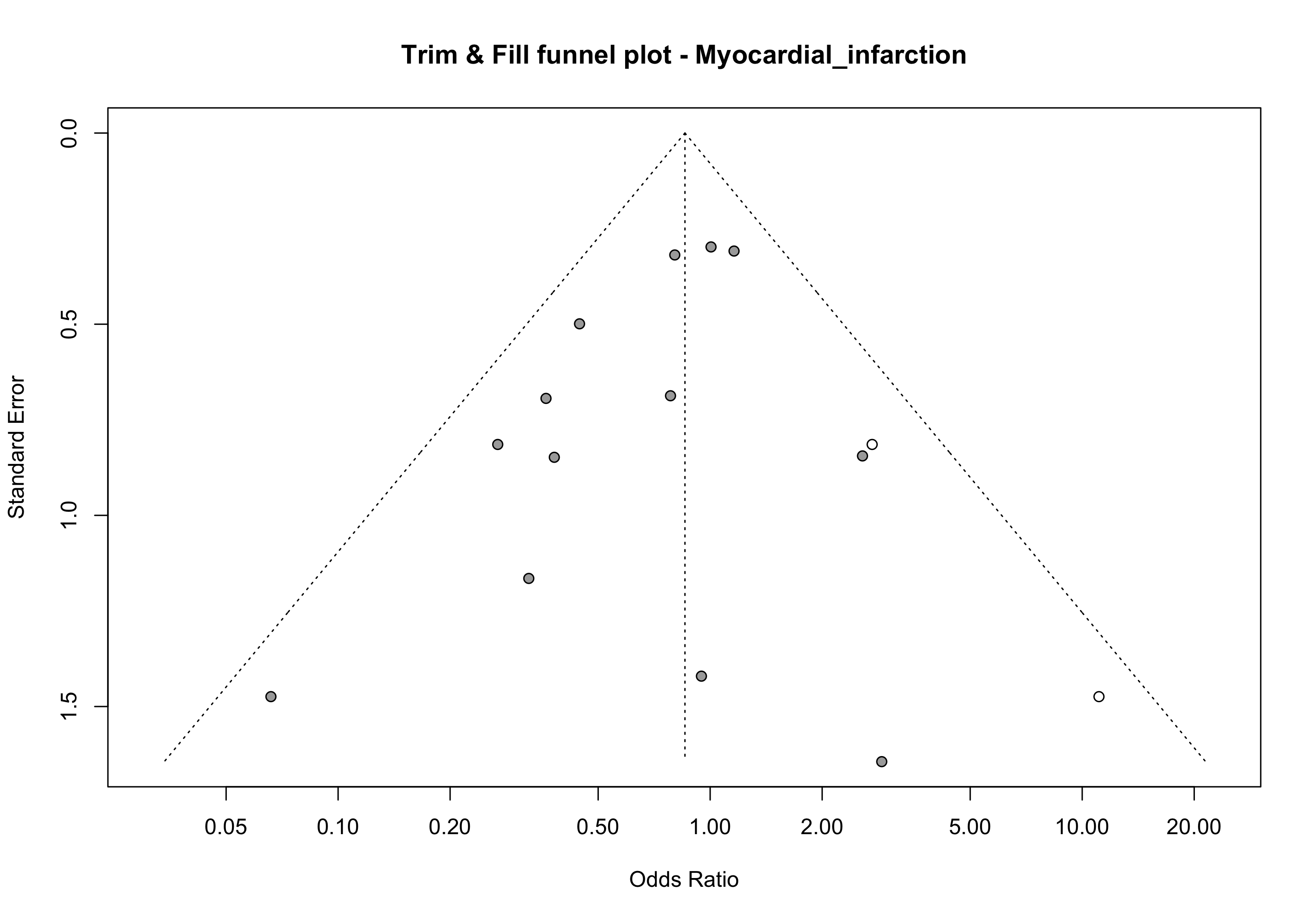

Supplement: Supplementary file 5 [file Datasheet1.zip › R scripts/7/Myocardial_infarction_trimfill_funnel.tiff]

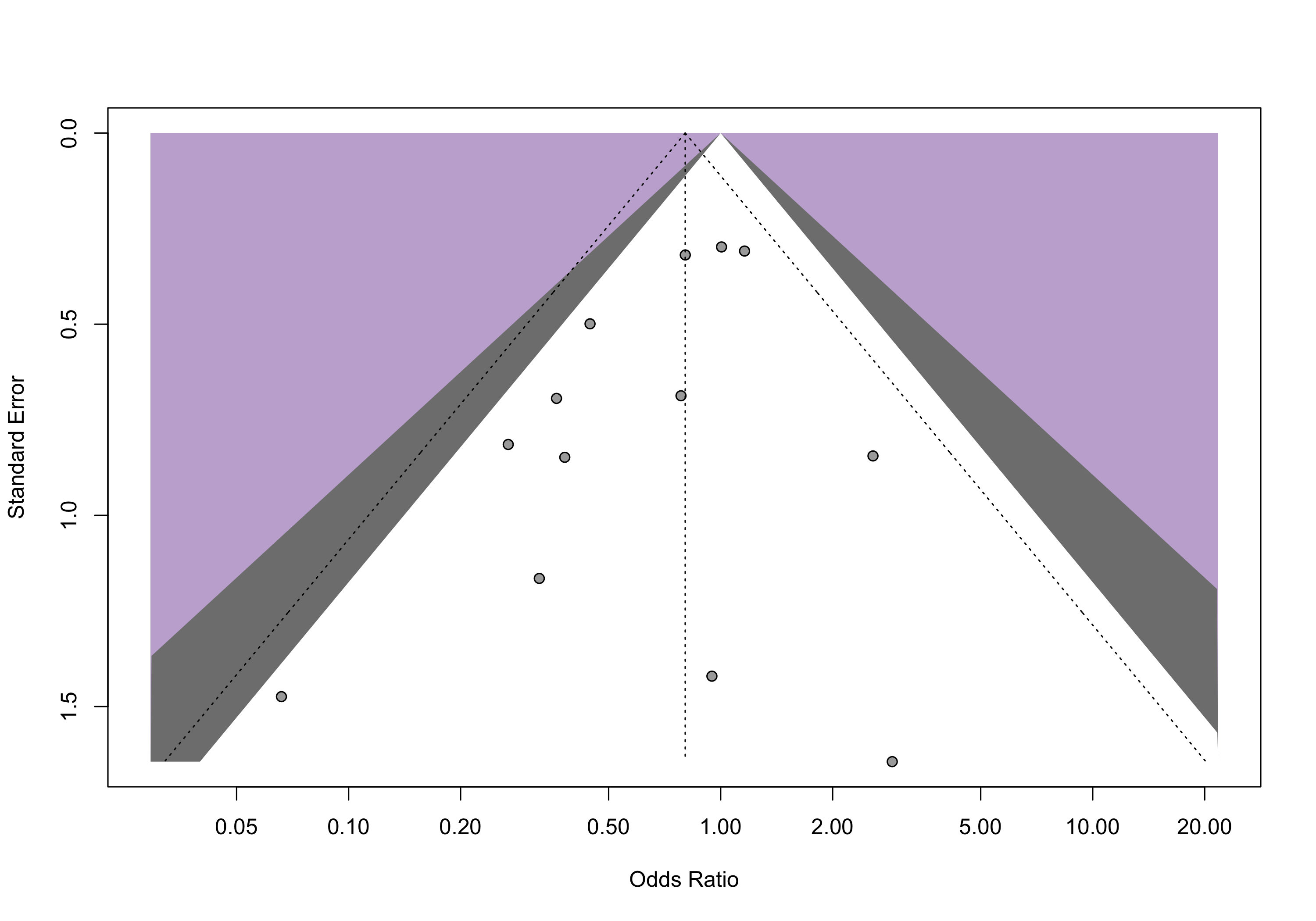

Supplement: Supplementary file 5 [file Datasheet1.zip › R scripts/7/Myocardial_infarction_funnel.tiff]

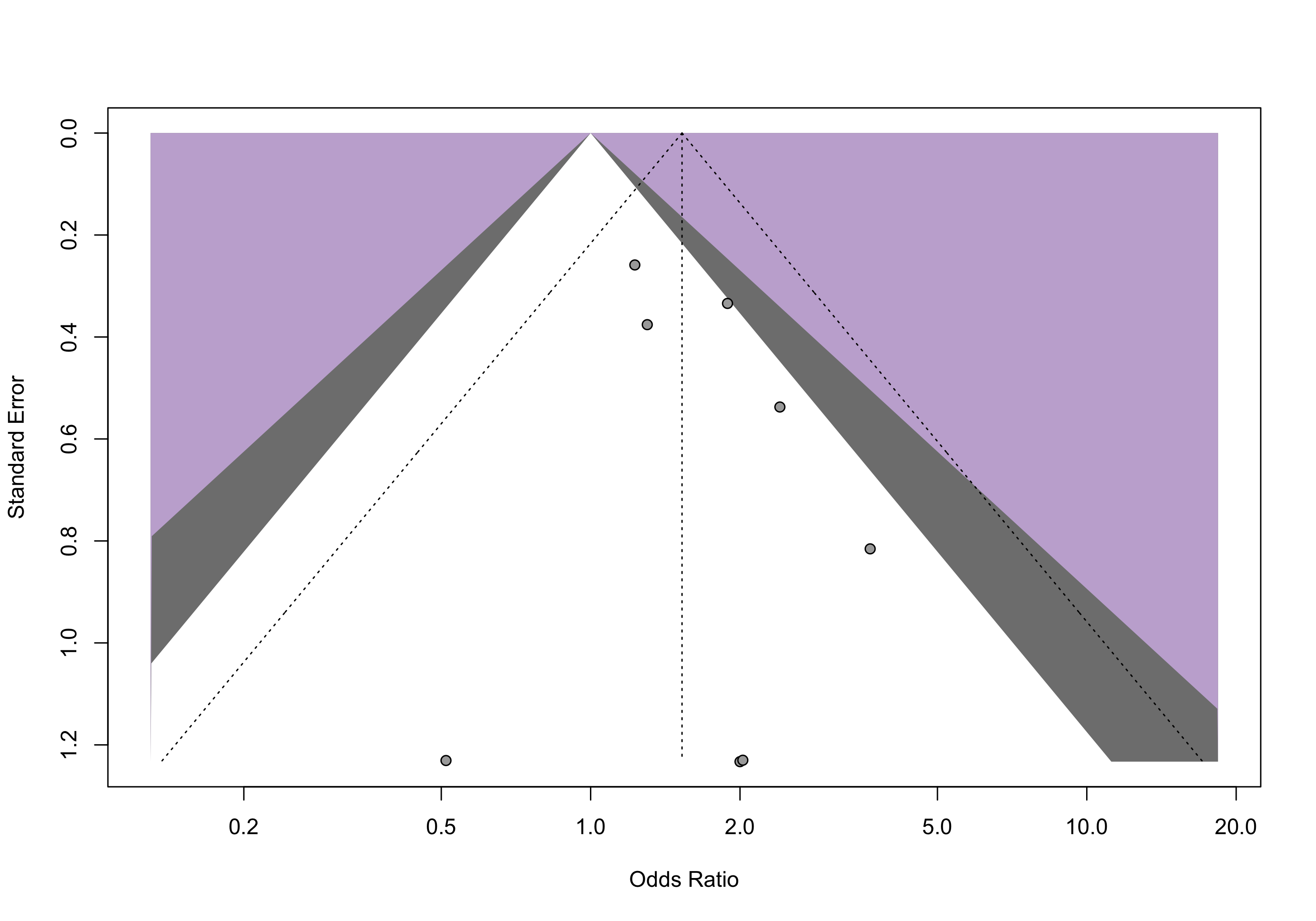

Supplement: Supplementary file 5 [file Datasheet1.zip › R scripts/6/Cardiac_death_funnel.tiff]

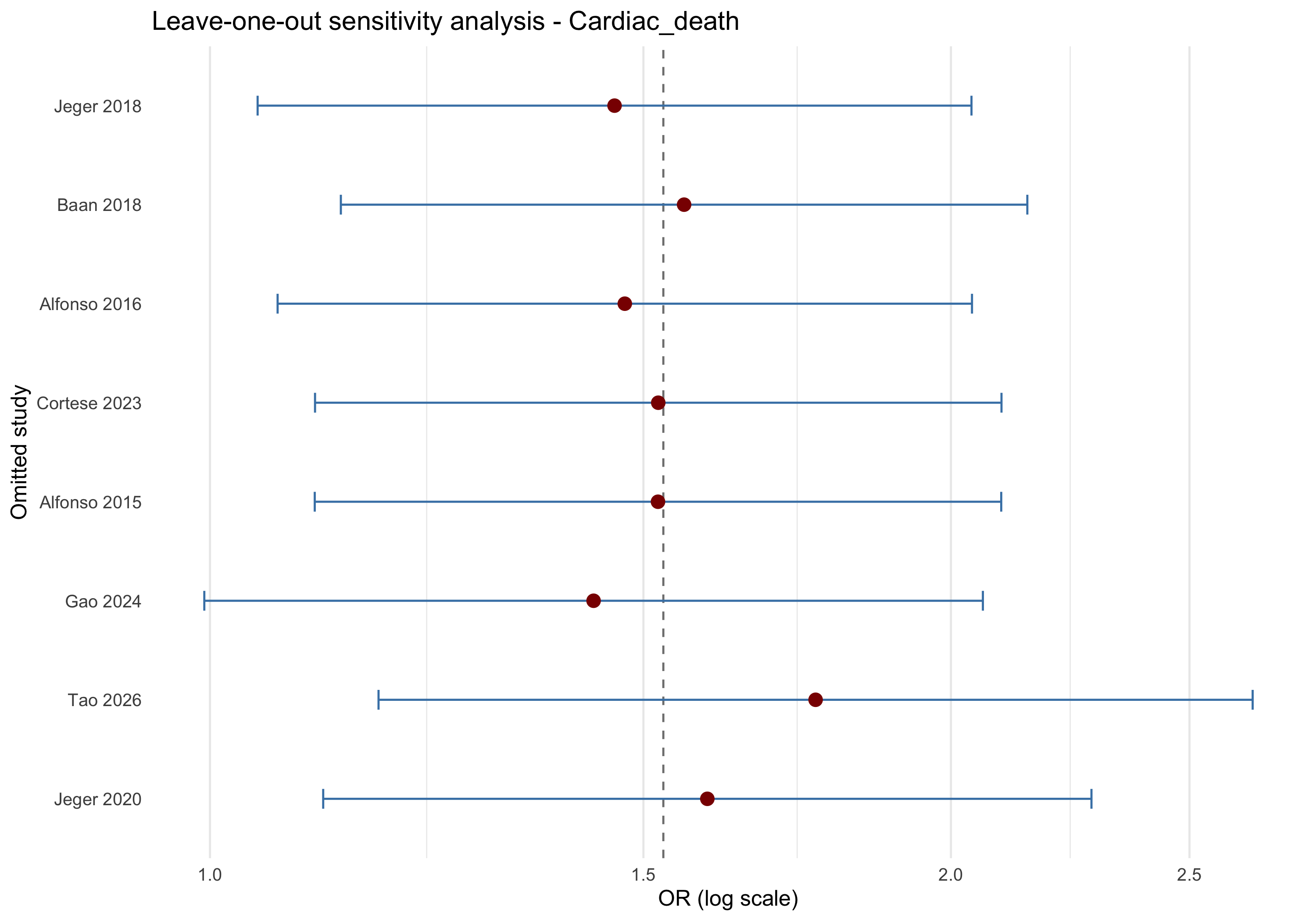

Supplement: Supplementary file 5 [file Datasheet1.zip › R scripts/6/Cardiac_death_sensitivity_leave1out.tiff]

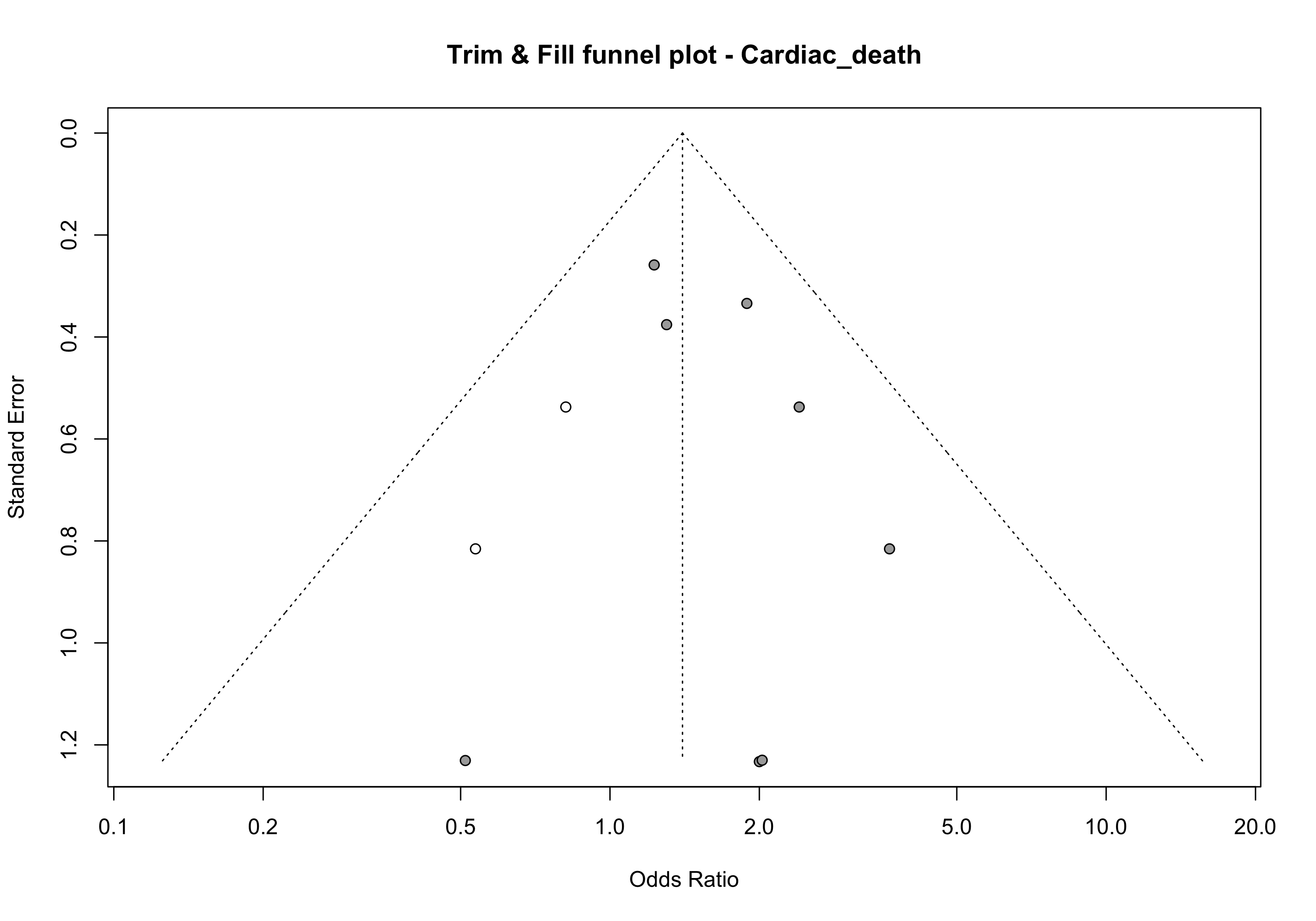

Supplement: Supplementary file 5 [file Datasheet1.zip › R scripts/6/Cardiac_death_trimfill_funnel.tiff]

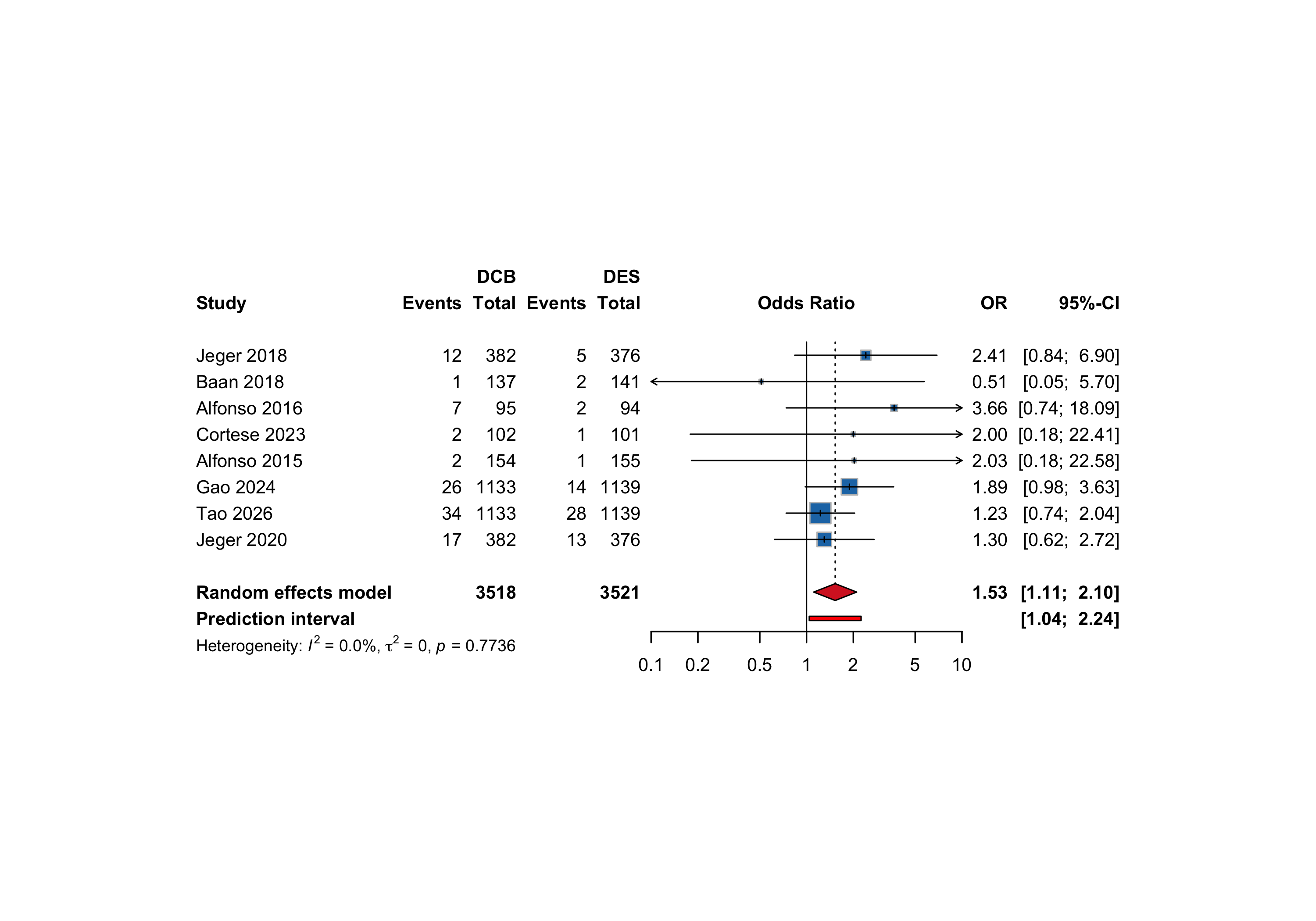

Supplement: Supplementary file 5 [file Datasheet1.zip › R scripts/6/Cardiac_death_forest.tiff]

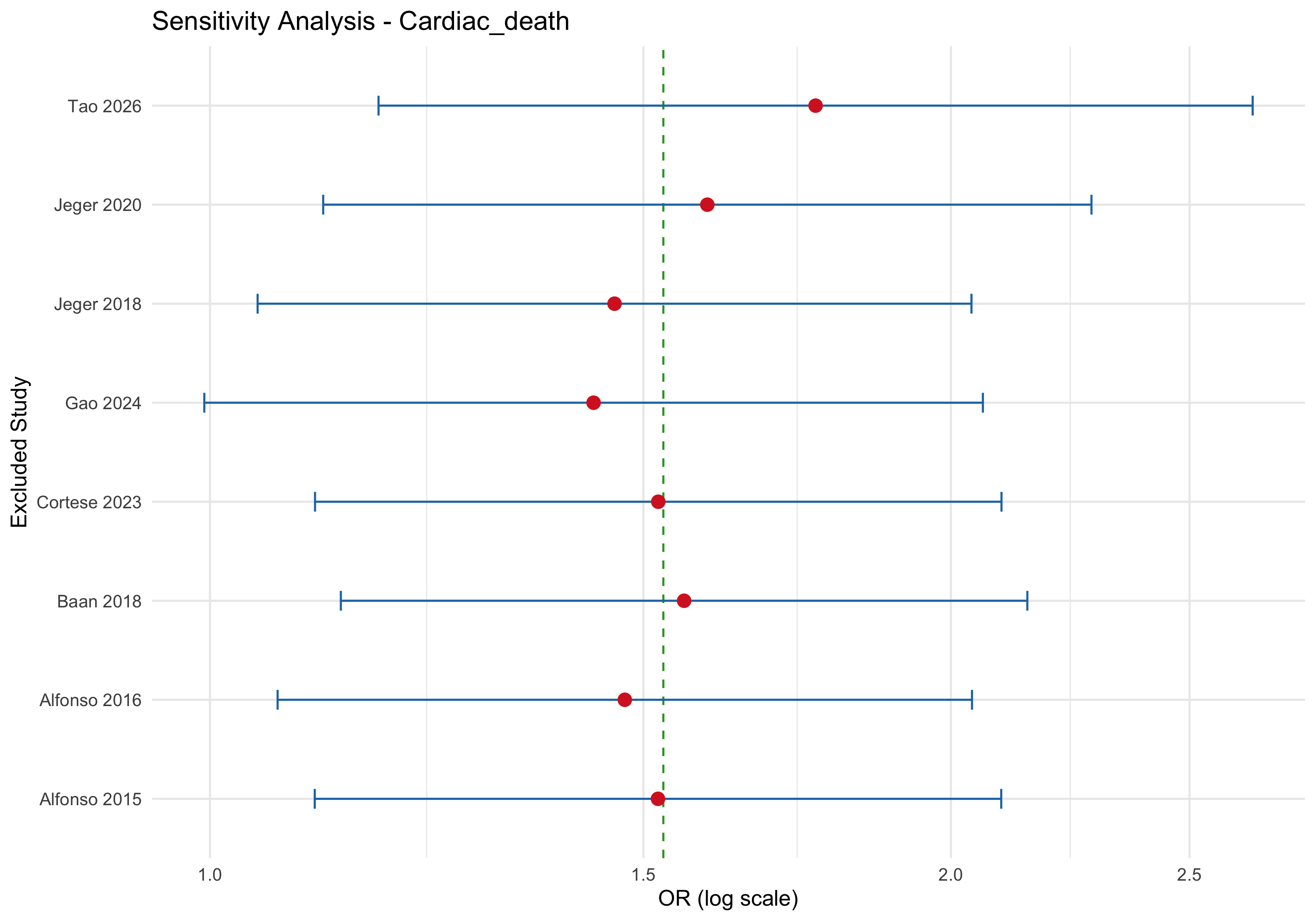

Supplement: Supplementary file 5 [file Datasheet1.zip › R scripts/6/Cardiac_death_sensitivity.tiff]

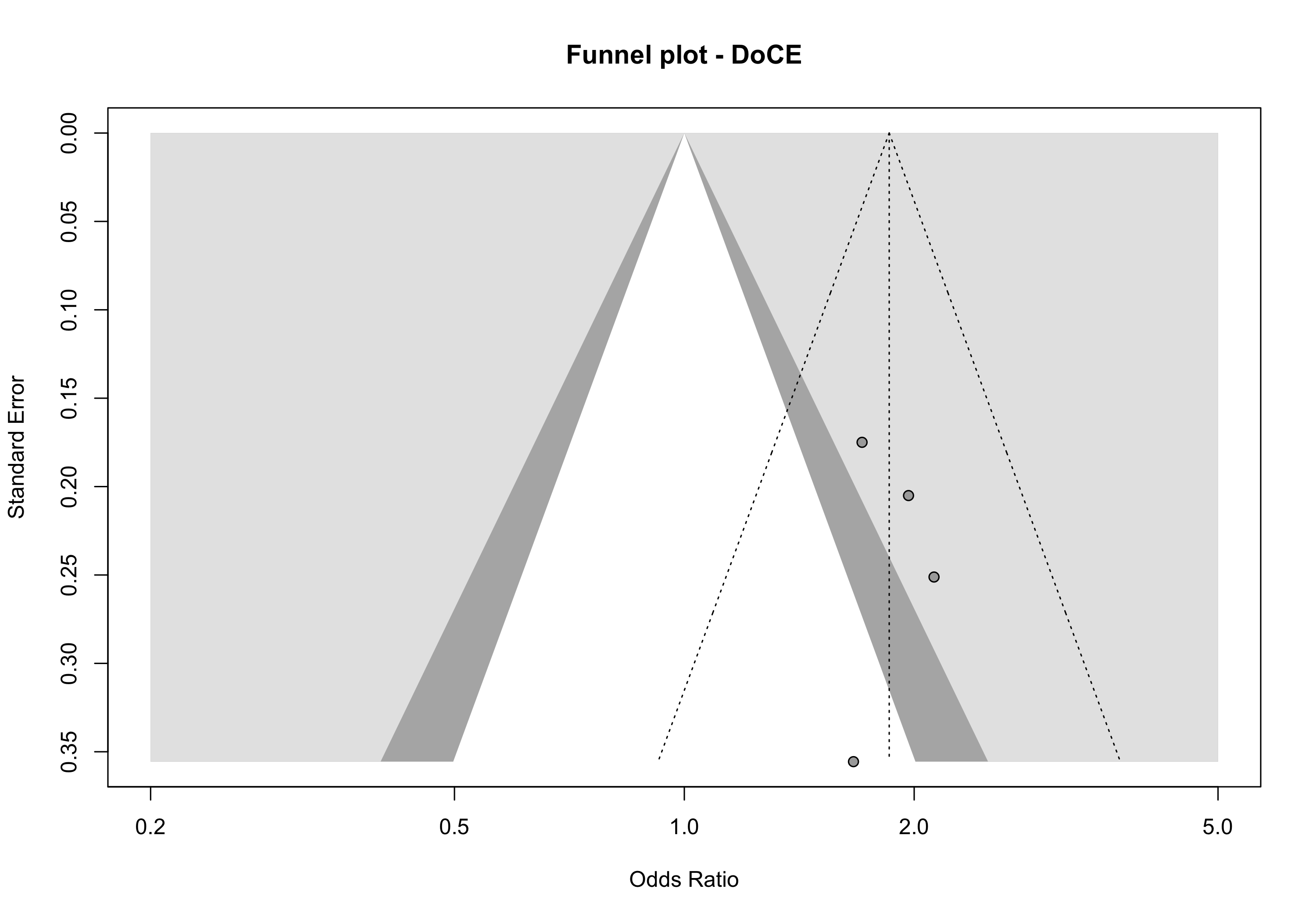

Supplement: Supplementary file 5 [file Datasheet1.zip › R scripts/10/DoCE_funnel.tiff]

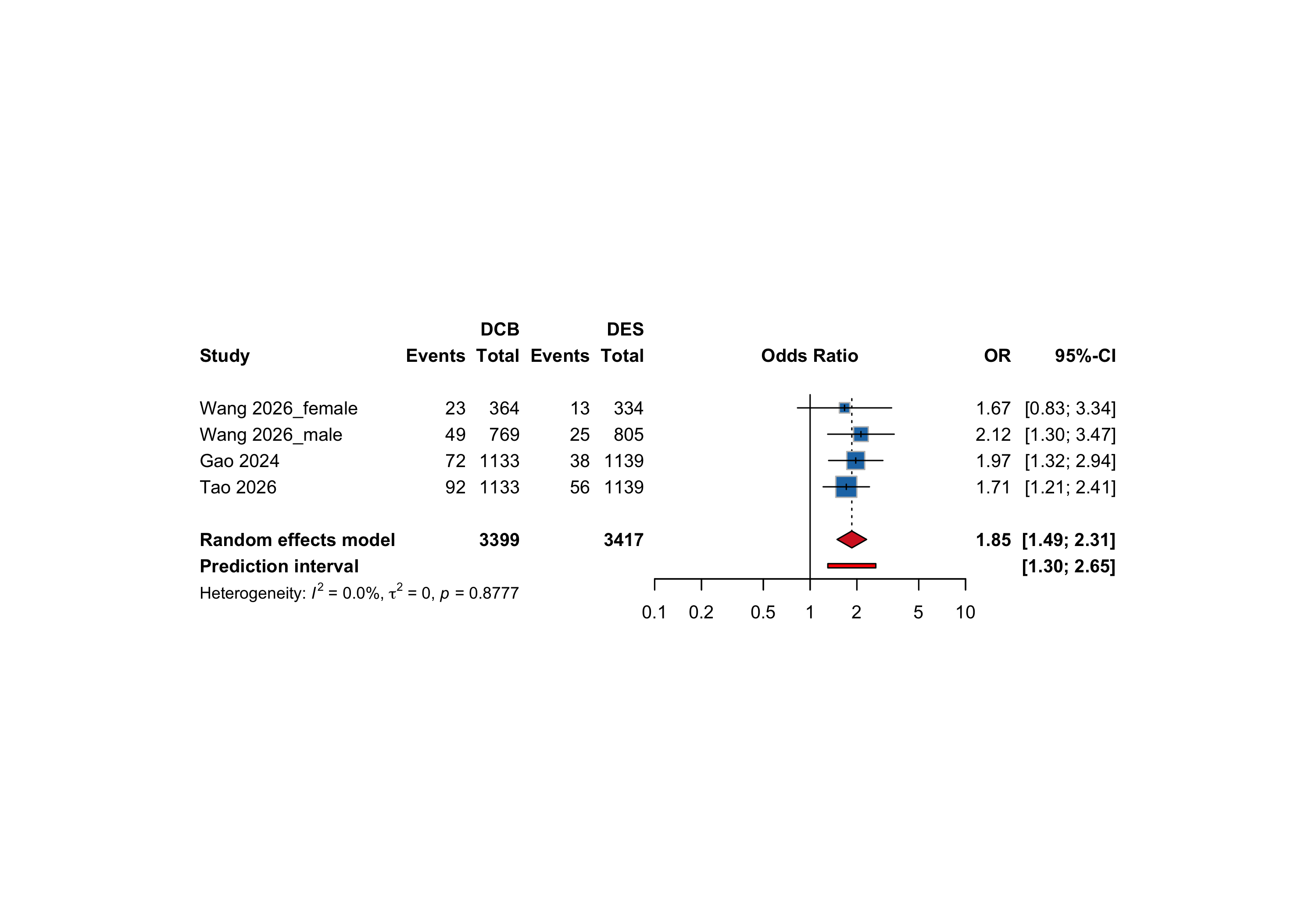

Supplement: Supplementary file 5 [file Datasheet1.zip › R scripts/10/DoCE_forest.tiff]

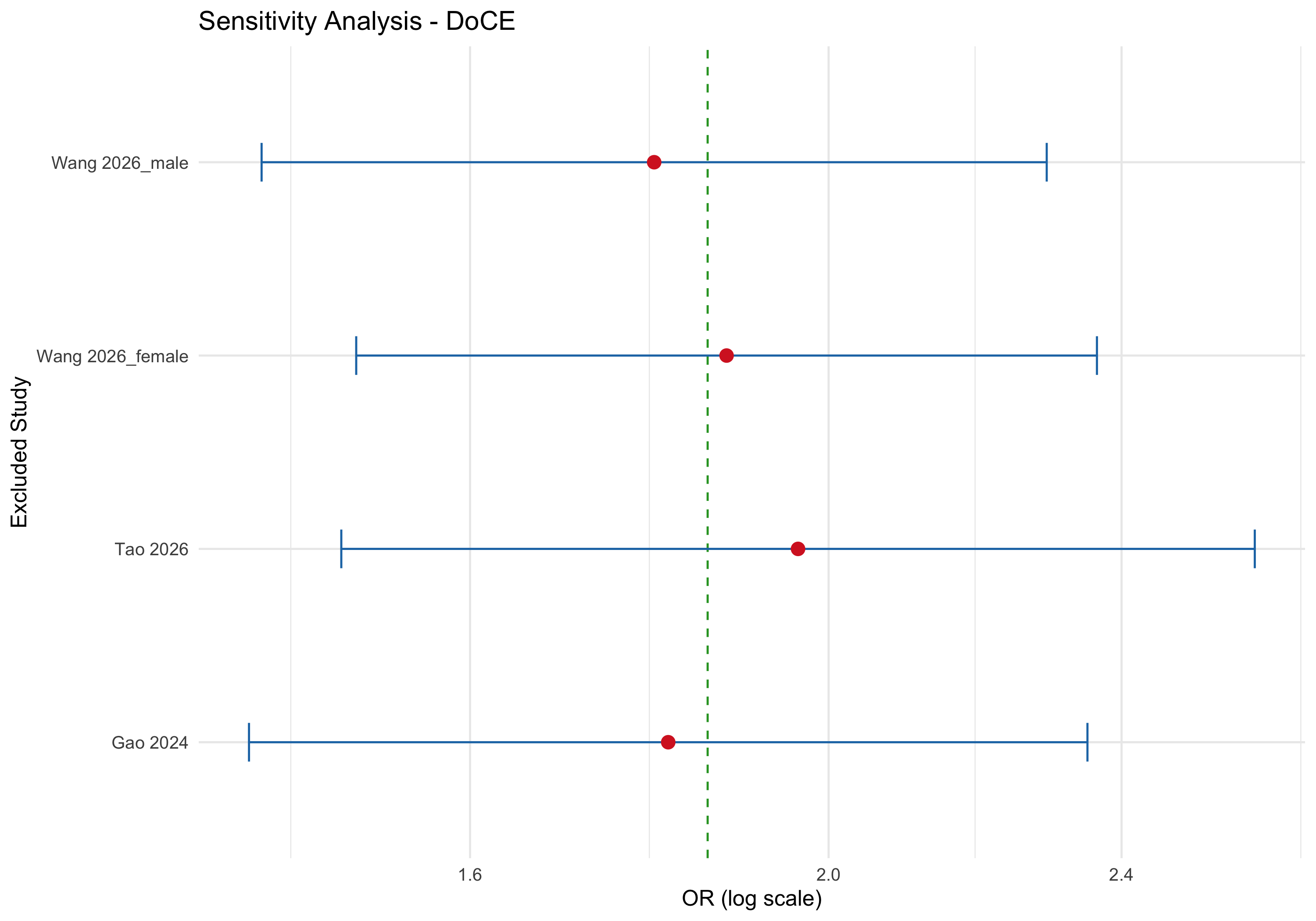

Supplement: Supplementary file 5 [file Datasheet1.zip › R scripts/10/DoCE_sensitivity.tiff]

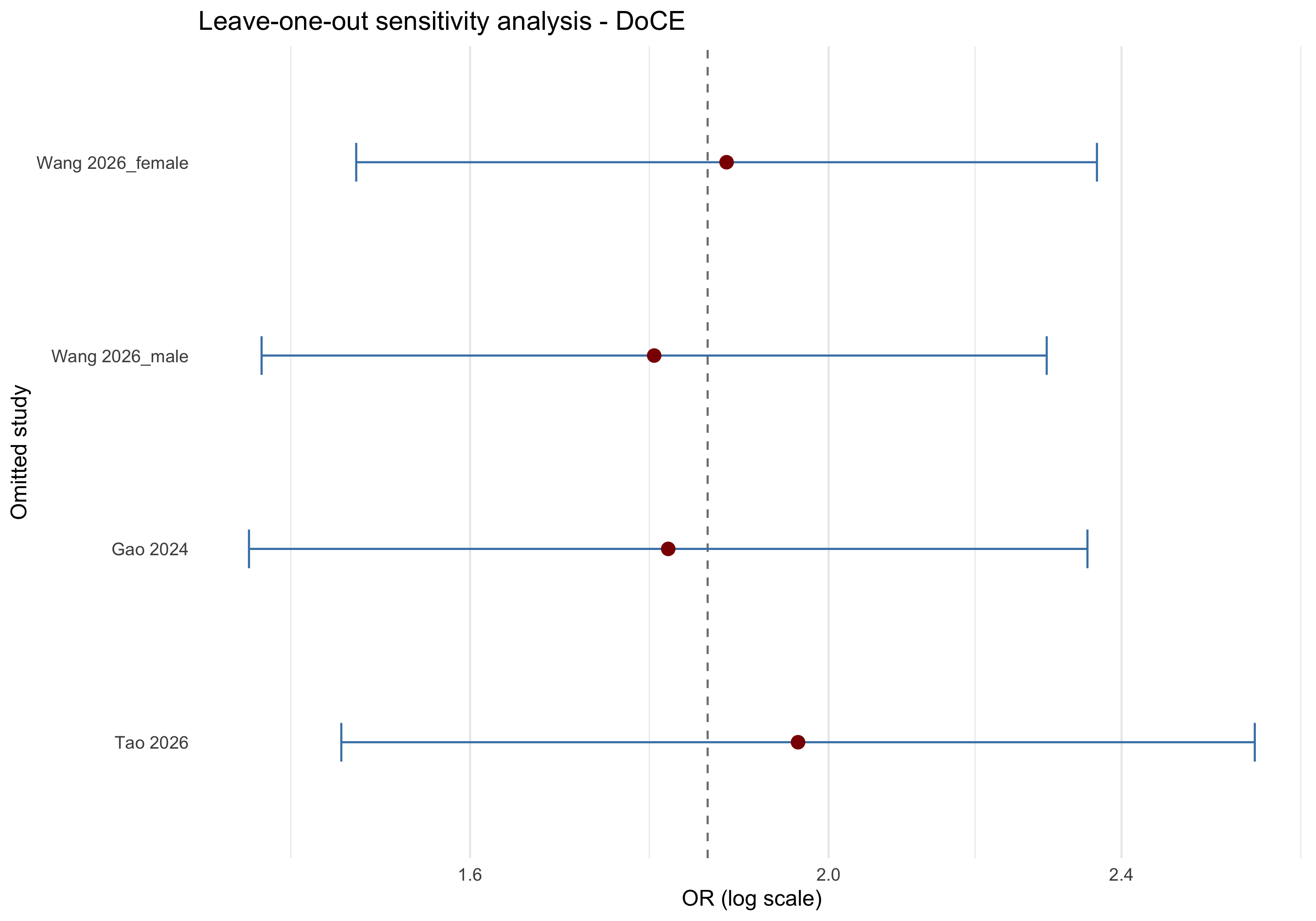

Supplement: Supplementary file 5 [file Datasheet1.zip › R scripts/10/DoCE_sensitivity_leave1out.tiff]

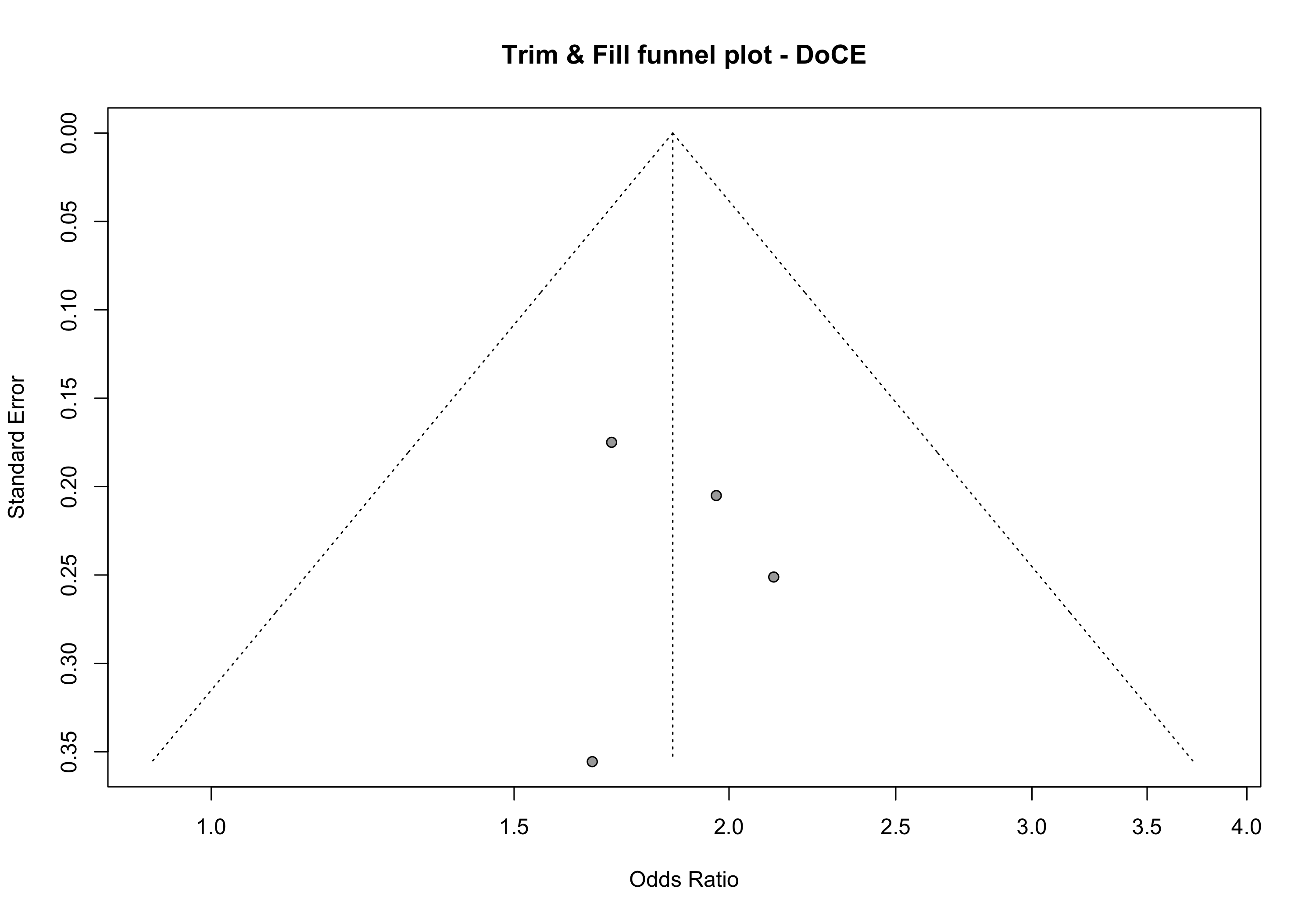

Supplement: Supplementary file 5 [file Datasheet1.zip › R scripts/10/DoCE_trimfill_funnel.tiff]

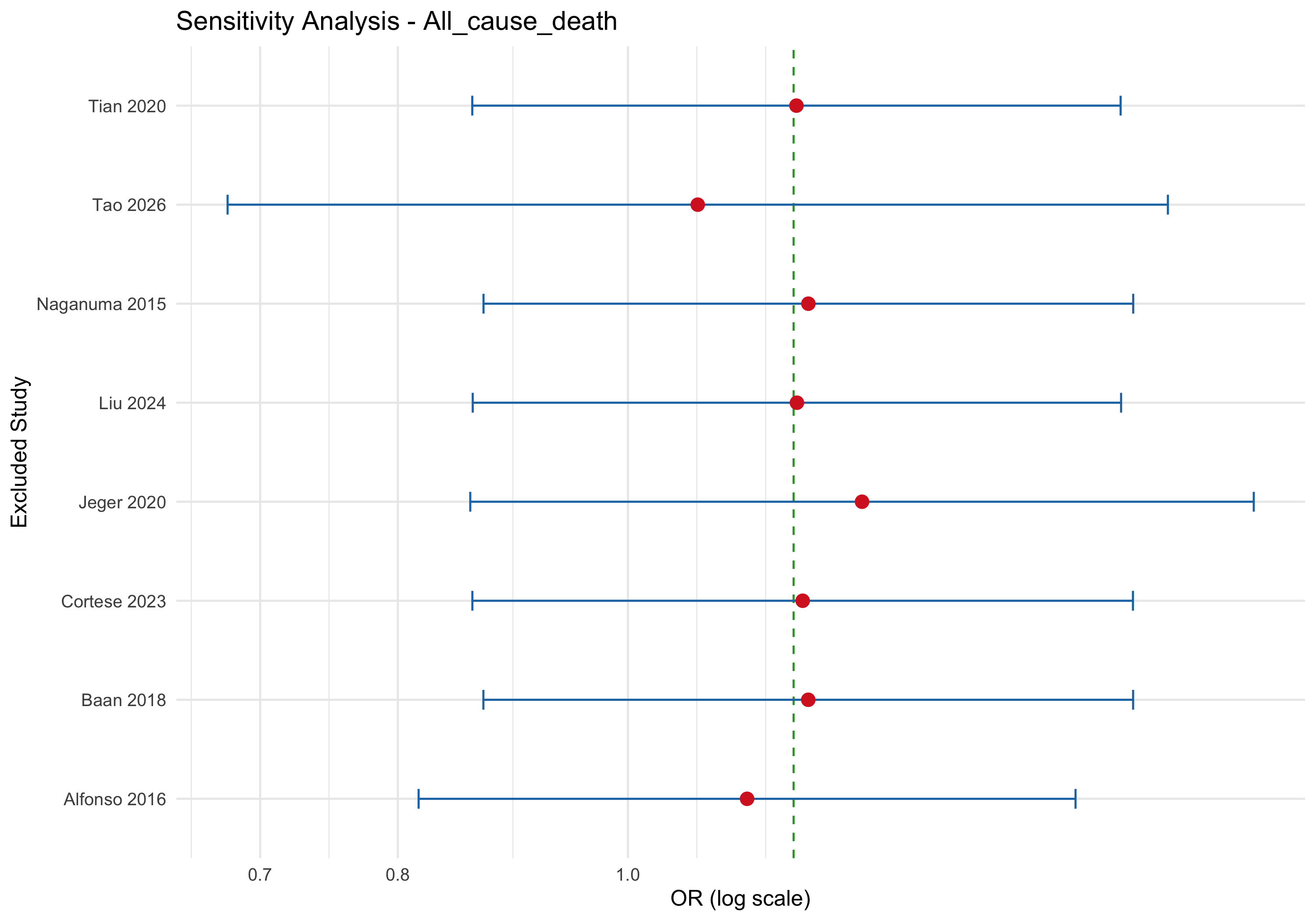

Supplement: Supplementary file 5 [file Datasheet1.zip › R scripts/8/All_cause_death_sensitivity.tiff]

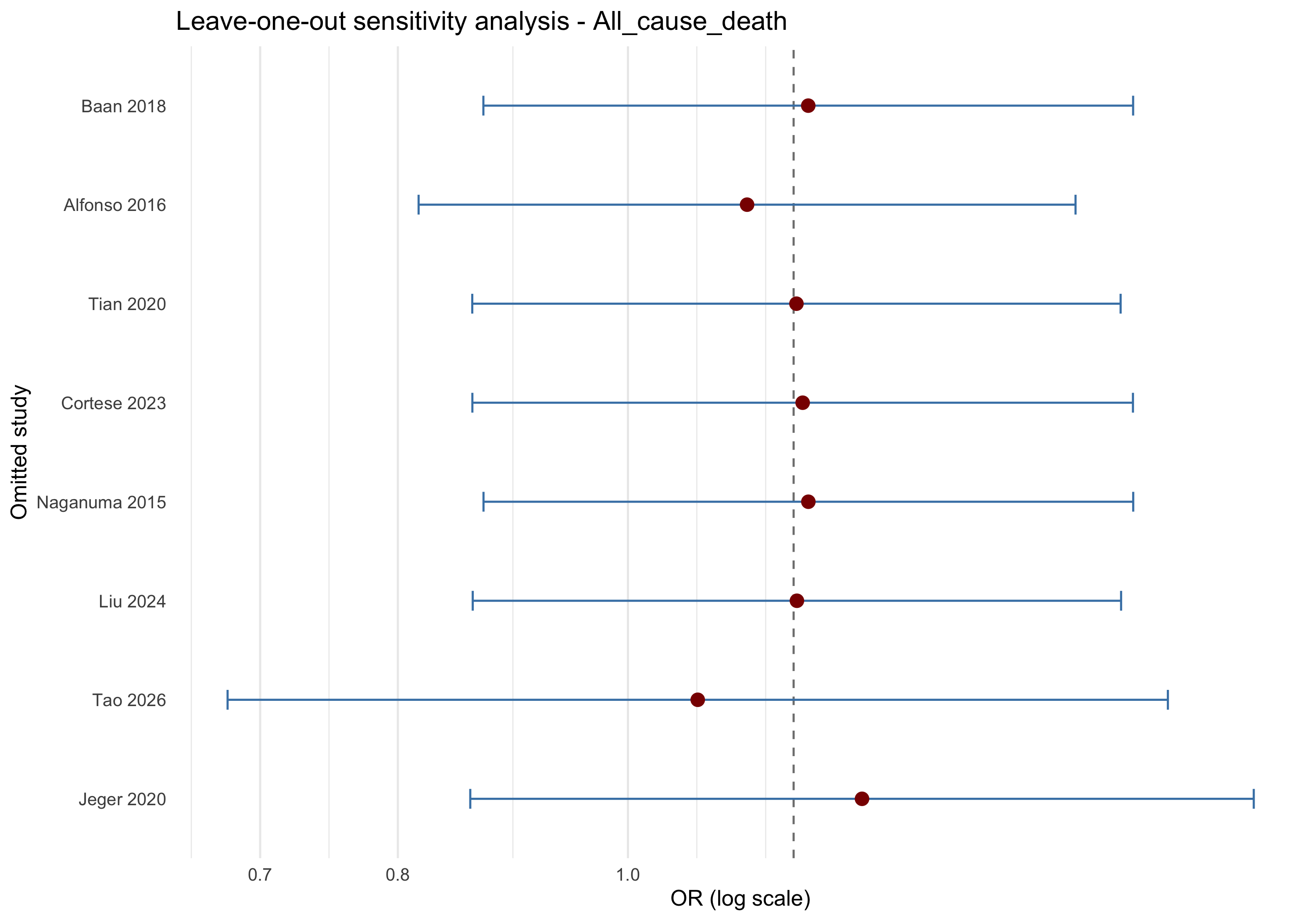

Supplement: Supplementary file 5 [file Datasheet1.zip › R scripts/8/All_cause_death_sensitivity_leave1out.tiff]

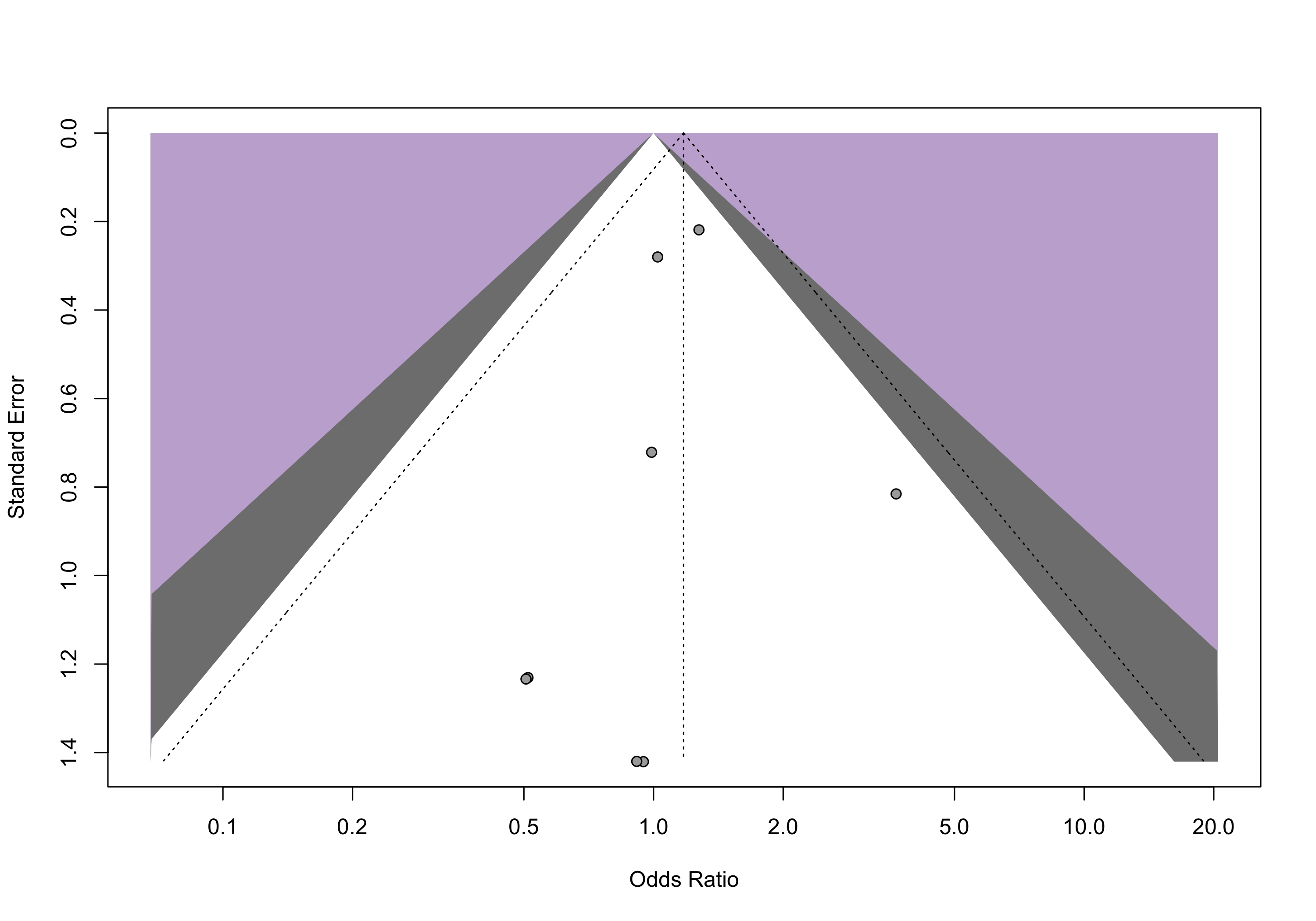

Supplement: Supplementary file 5 [file Datasheet1.zip › R scripts/8/All_cause_death_funnel.tiff]

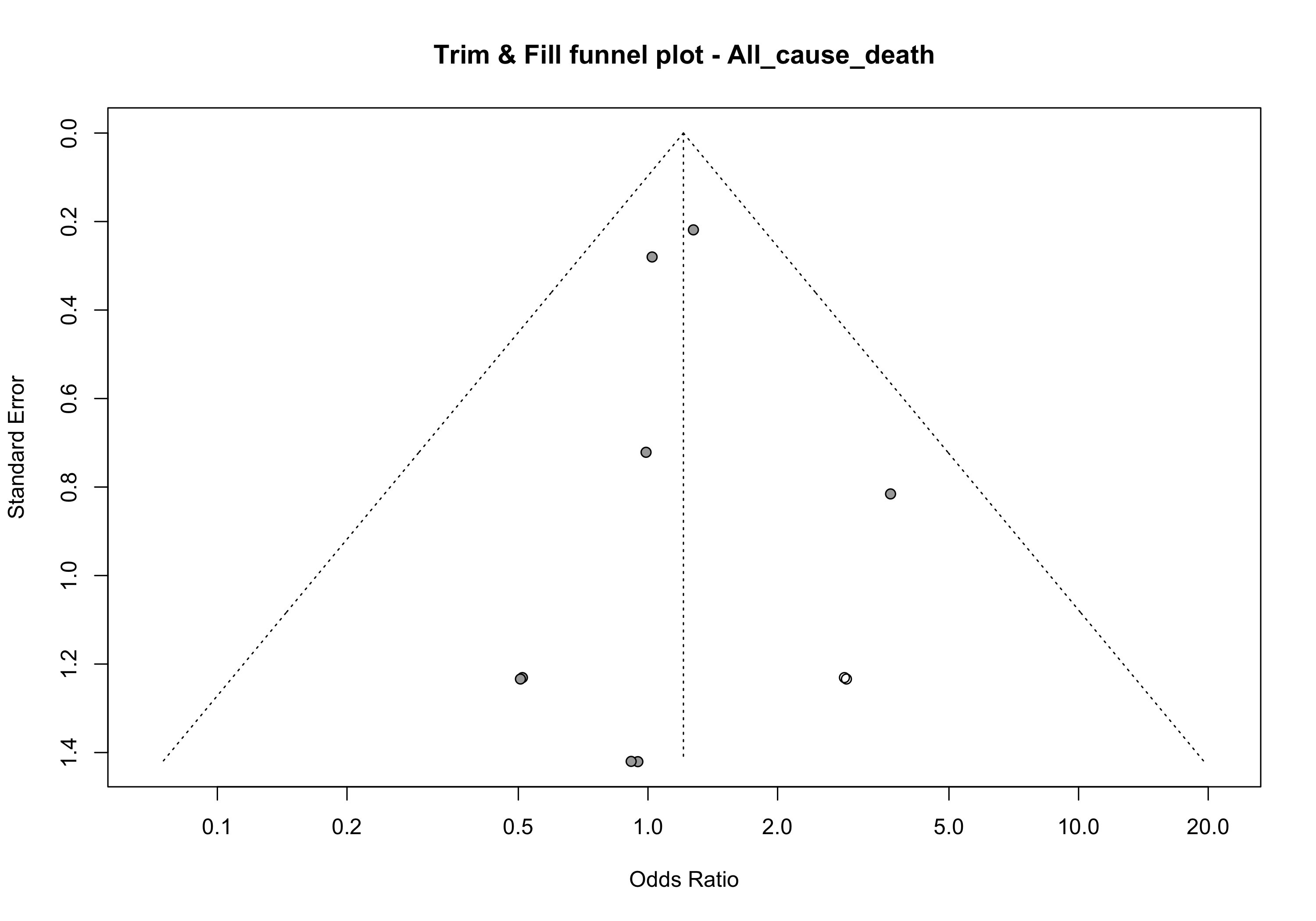

Supplement: Supplementary file 5 [file Datasheet1.zip › R scripts/8/All_cause_death_trimfill_funnel.tiff]

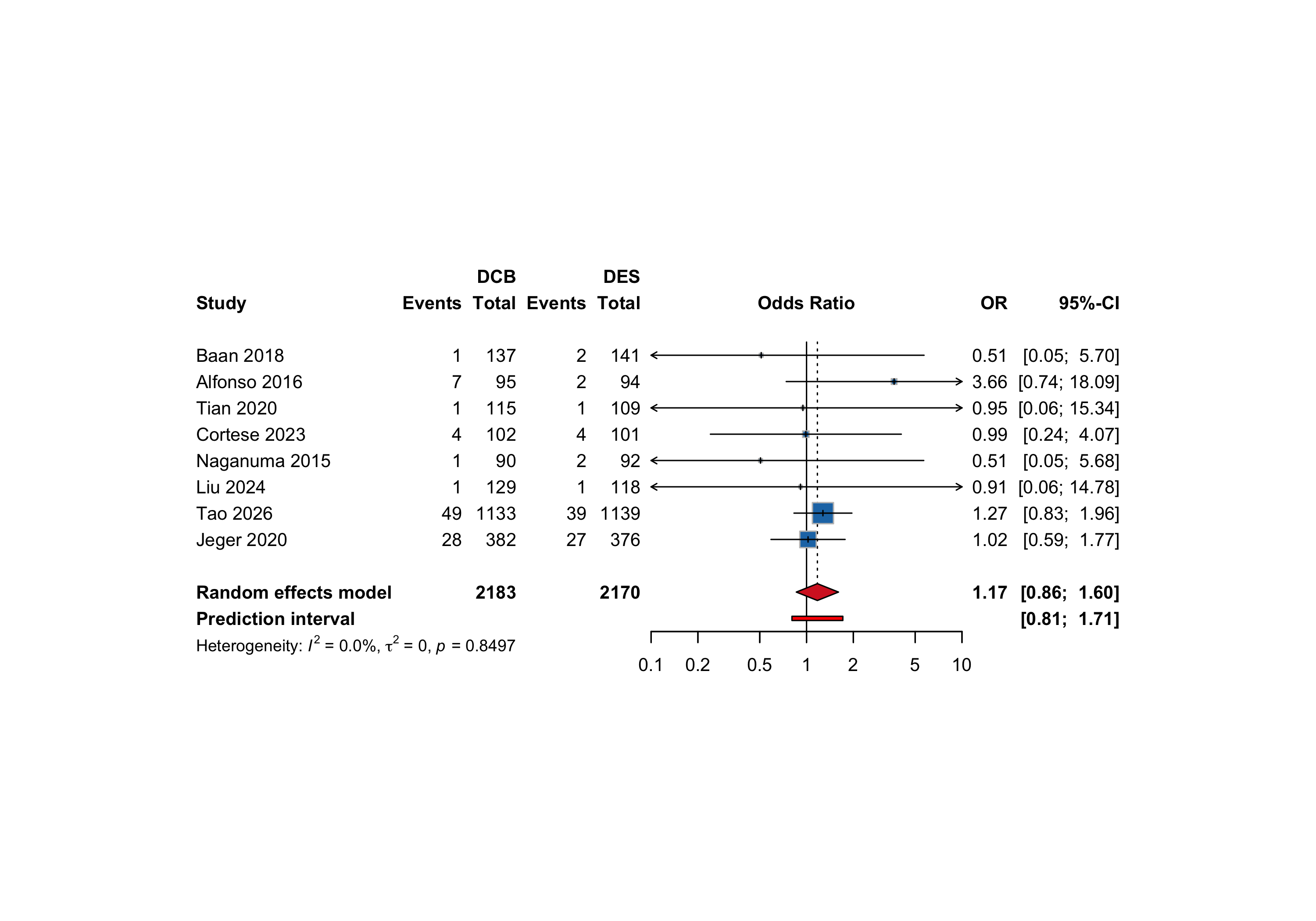

Supplement: Supplementary file 5 [file Datasheet1.zip › R scripts/8/All_cause_death_forest.tiff]

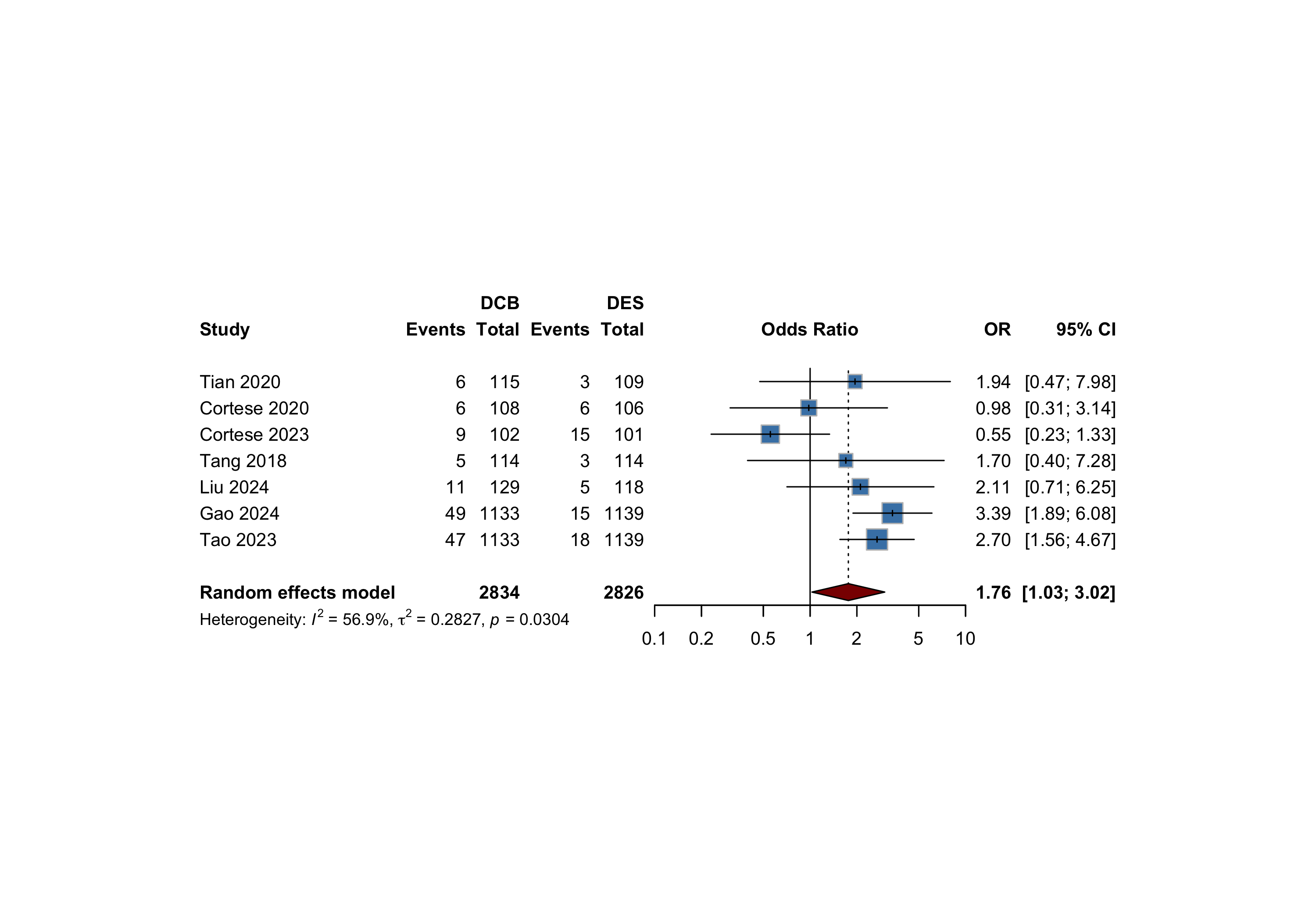

Supplement: Supplementary file 5 [file Datasheet1.zip › R scripts/16-S4/TLR_subgroup_de_novo_forest.tiff]

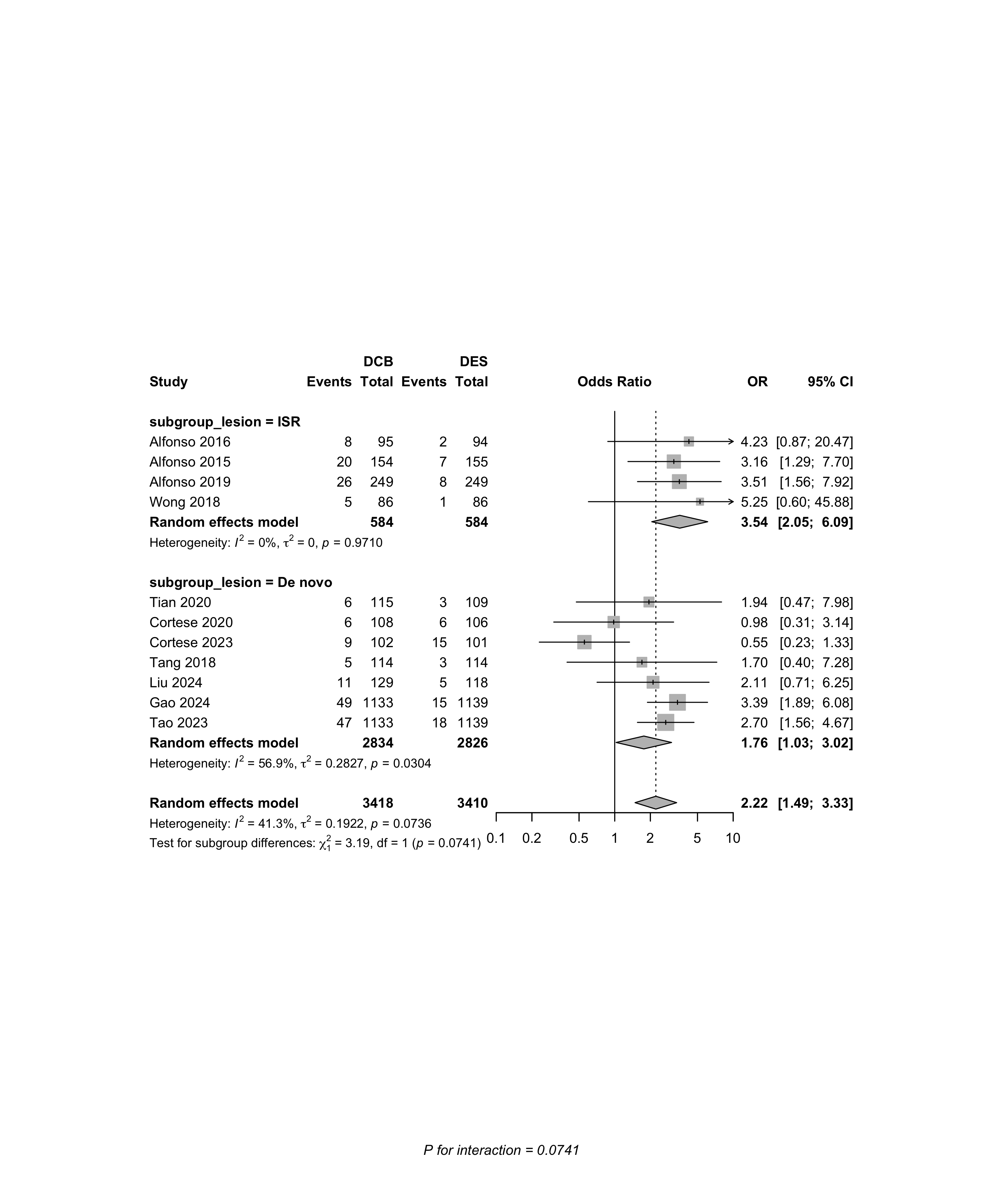

Supplement: Supplementary file 5 [file Datasheet1.zip › R scripts/16-S4/TLR_subgroup_lesion_type_forest.tiff]

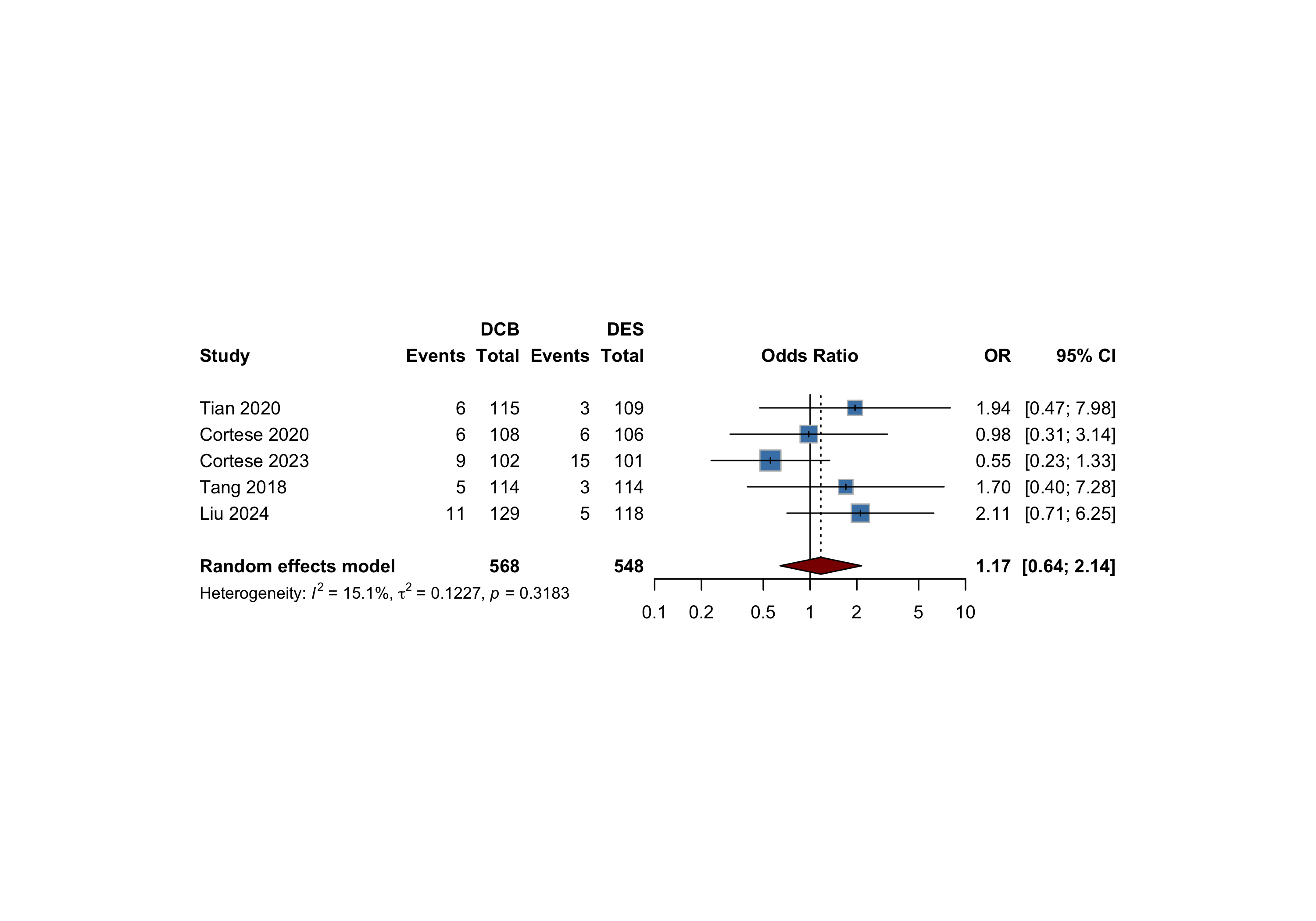

Supplement: Supplementary file 5 [file Datasheet1.zip › R scripts/16-S4/TLR_subgroup_small_vessel_forest.tiff]

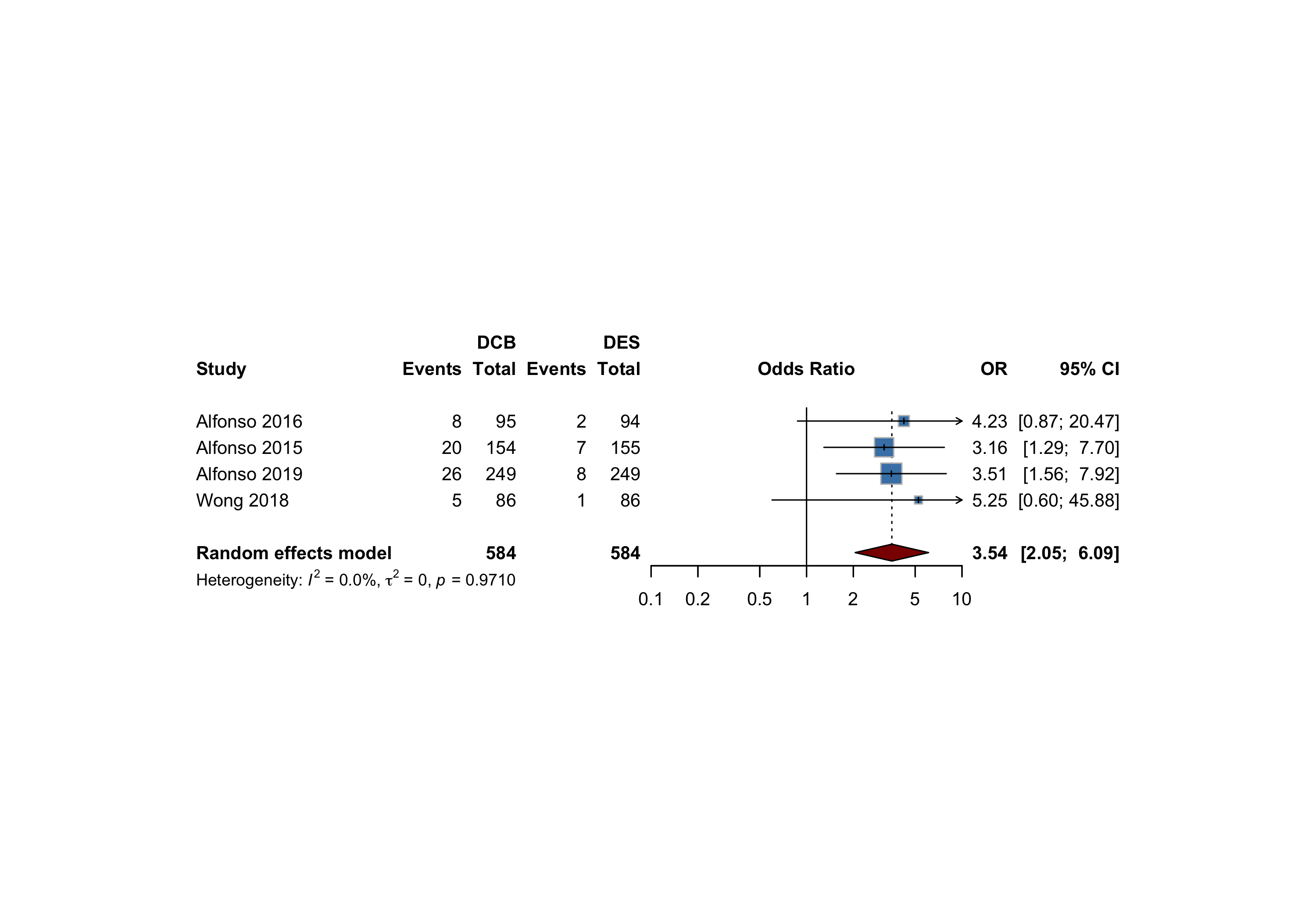

Supplement: Supplementary file 5 [file Datasheet1.zip › R scripts/16-S4/TLR_subgroup_ISR_forest.tiff]

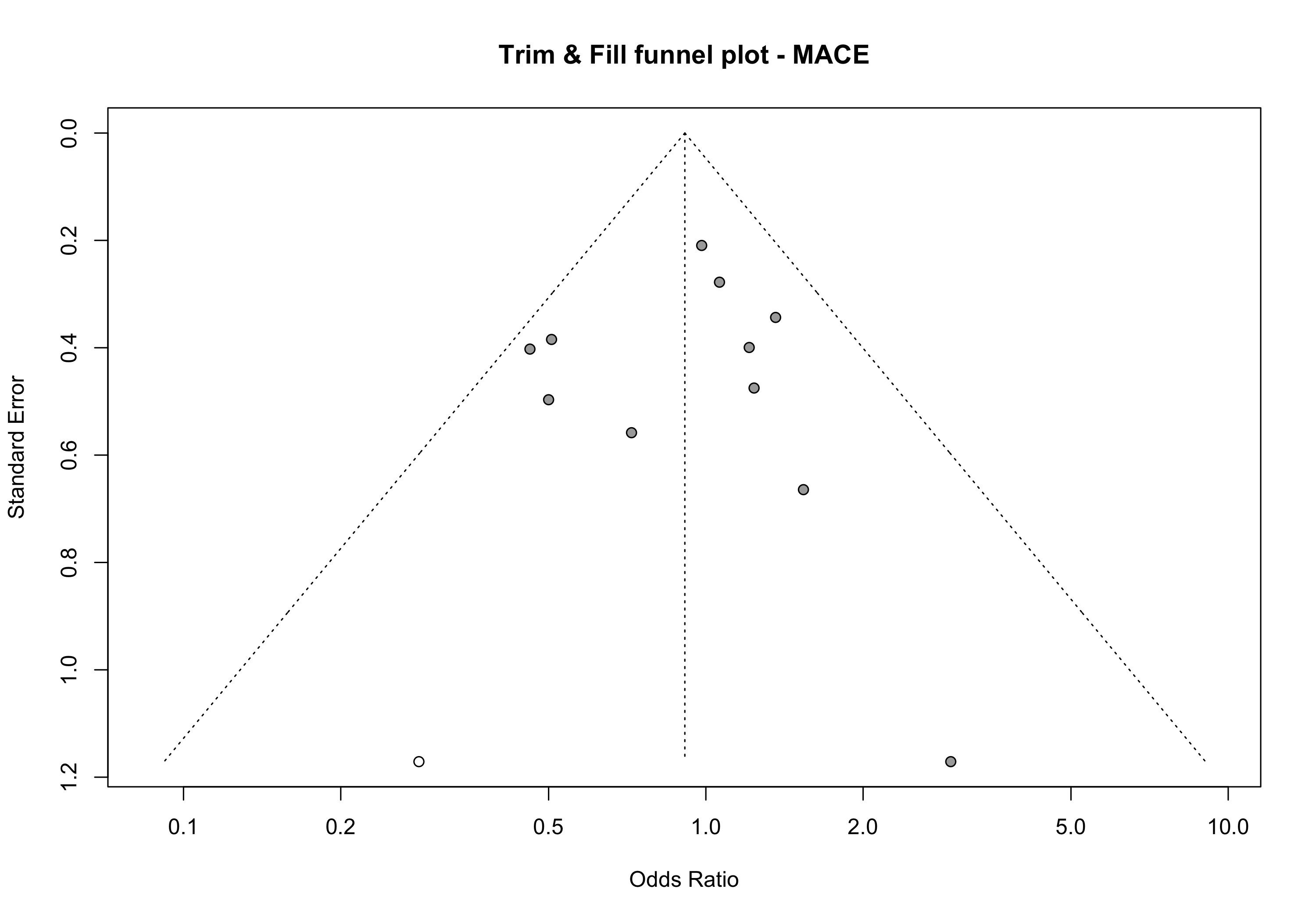

Supplement: Supplementary file 5 [file Datasheet1.zip › R scripts/4/MACE_trimfill_funnel.tiff]

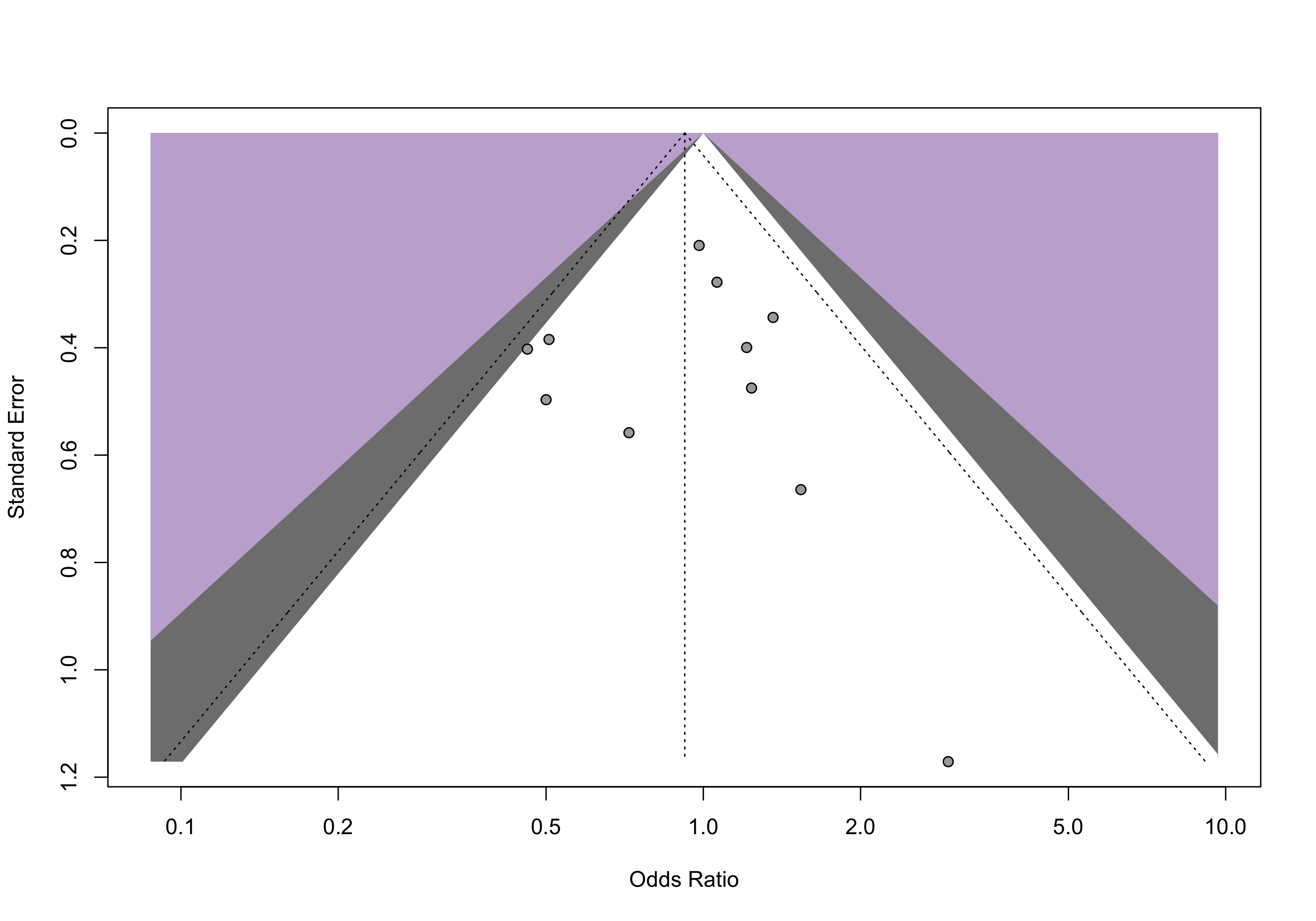

Supplement: Supplementary file 5 [file Datasheet1.zip › R scripts/4/MACE_funnel.tiff]

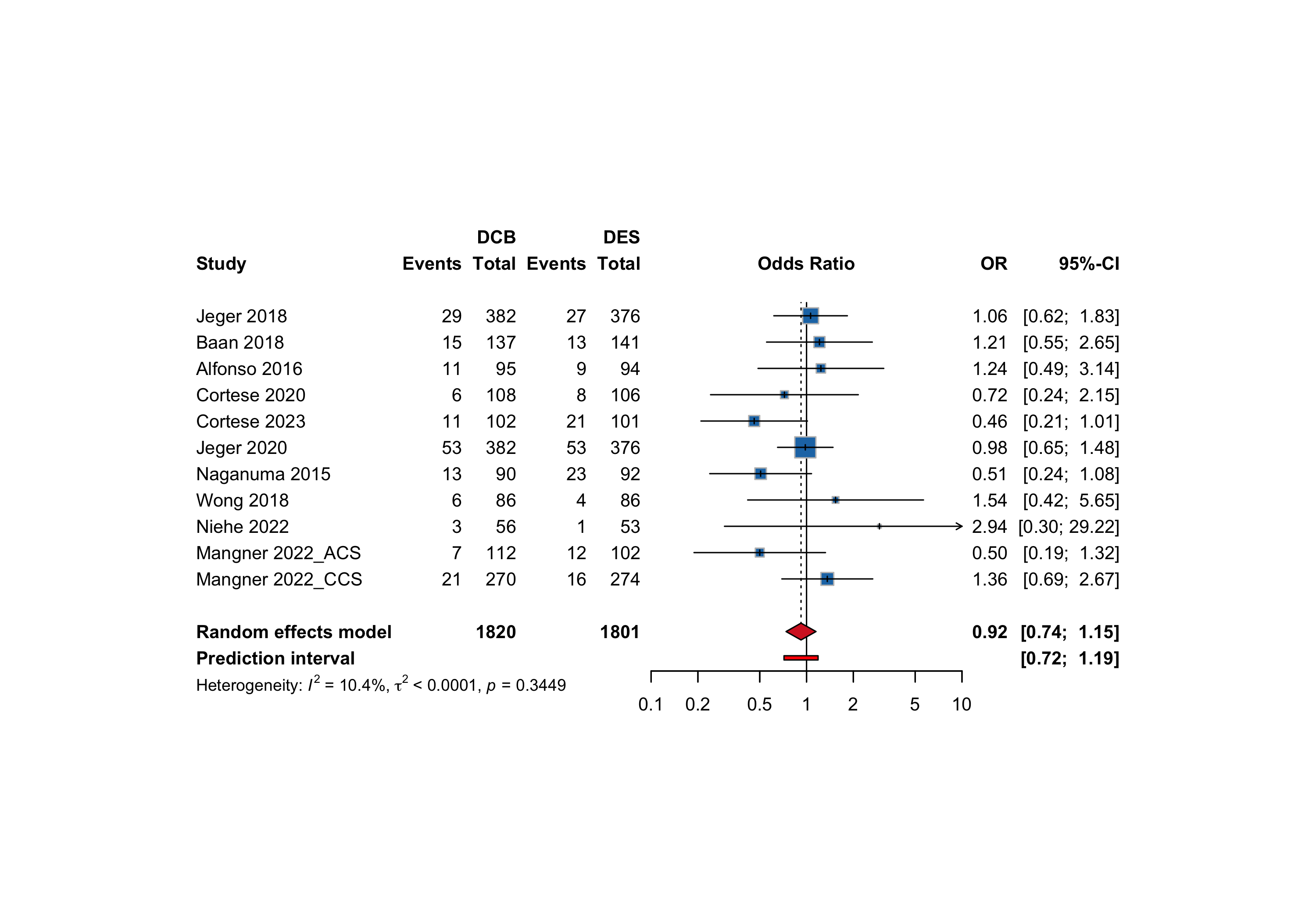

Supplement: Supplementary file 5 [file Datasheet1.zip › R scripts/4/MACE_forest.tiff]

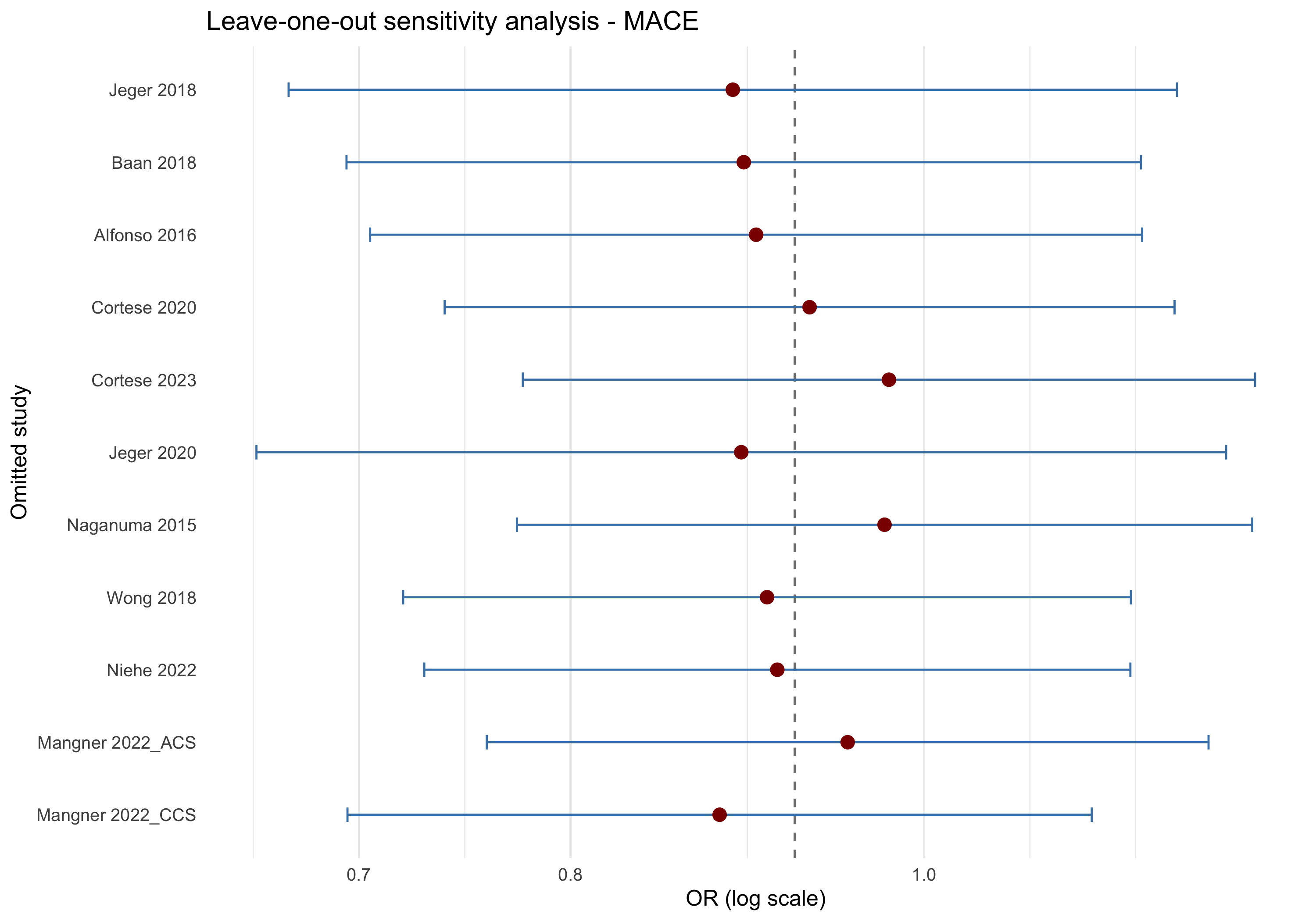

Supplement: Supplementary file 5 [file Datasheet1.zip › R scripts/4/MACE_sensitivity_leave1out.tiff]

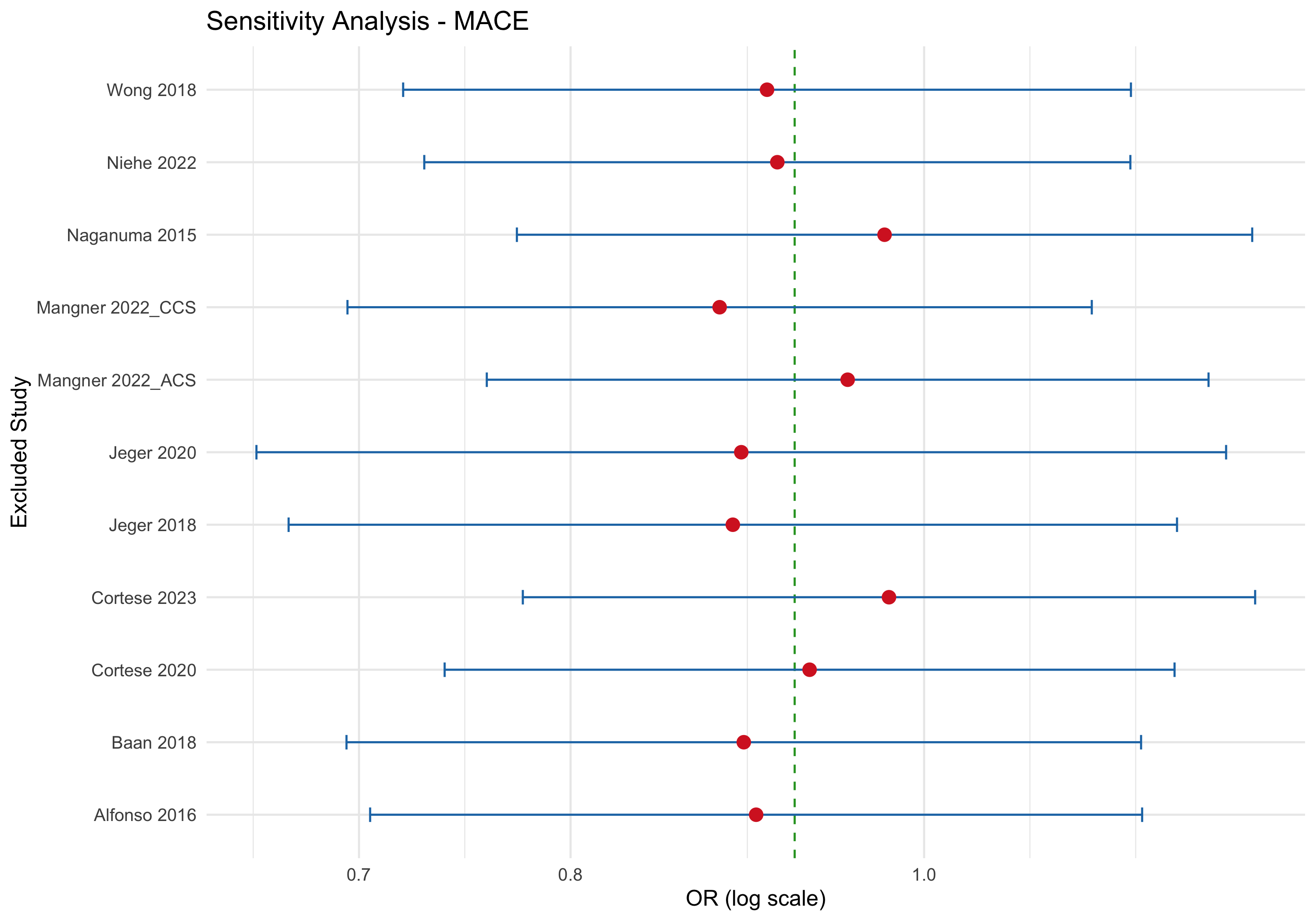

Supplement: Supplementary file 5 [file Datasheet1.zip › R scripts/4/MACE_sensitivity.tiff]

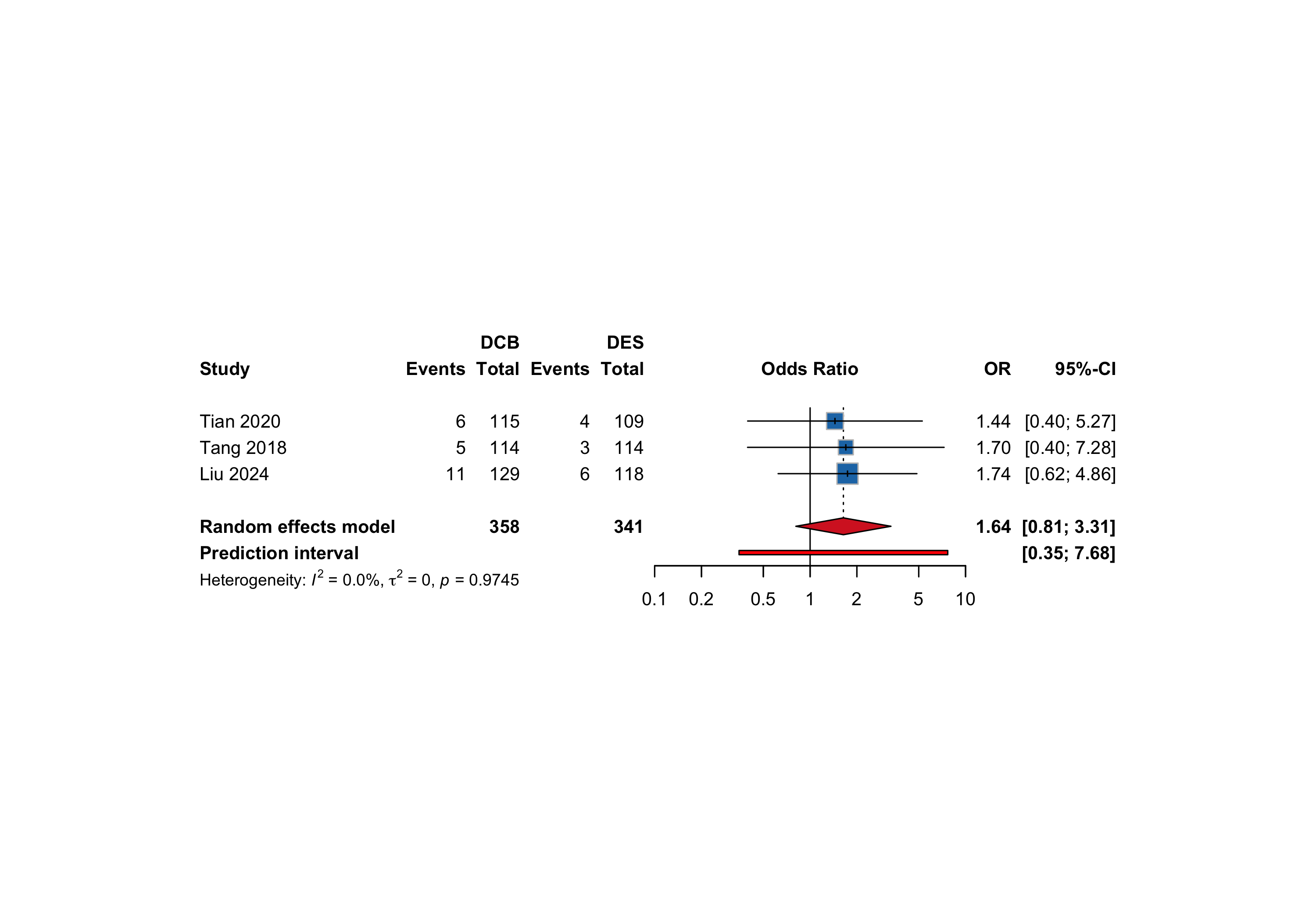

Supplement: Supplementary file 5 [file Datasheet1.zip › R scripts/12/TLF_forest.tiff]

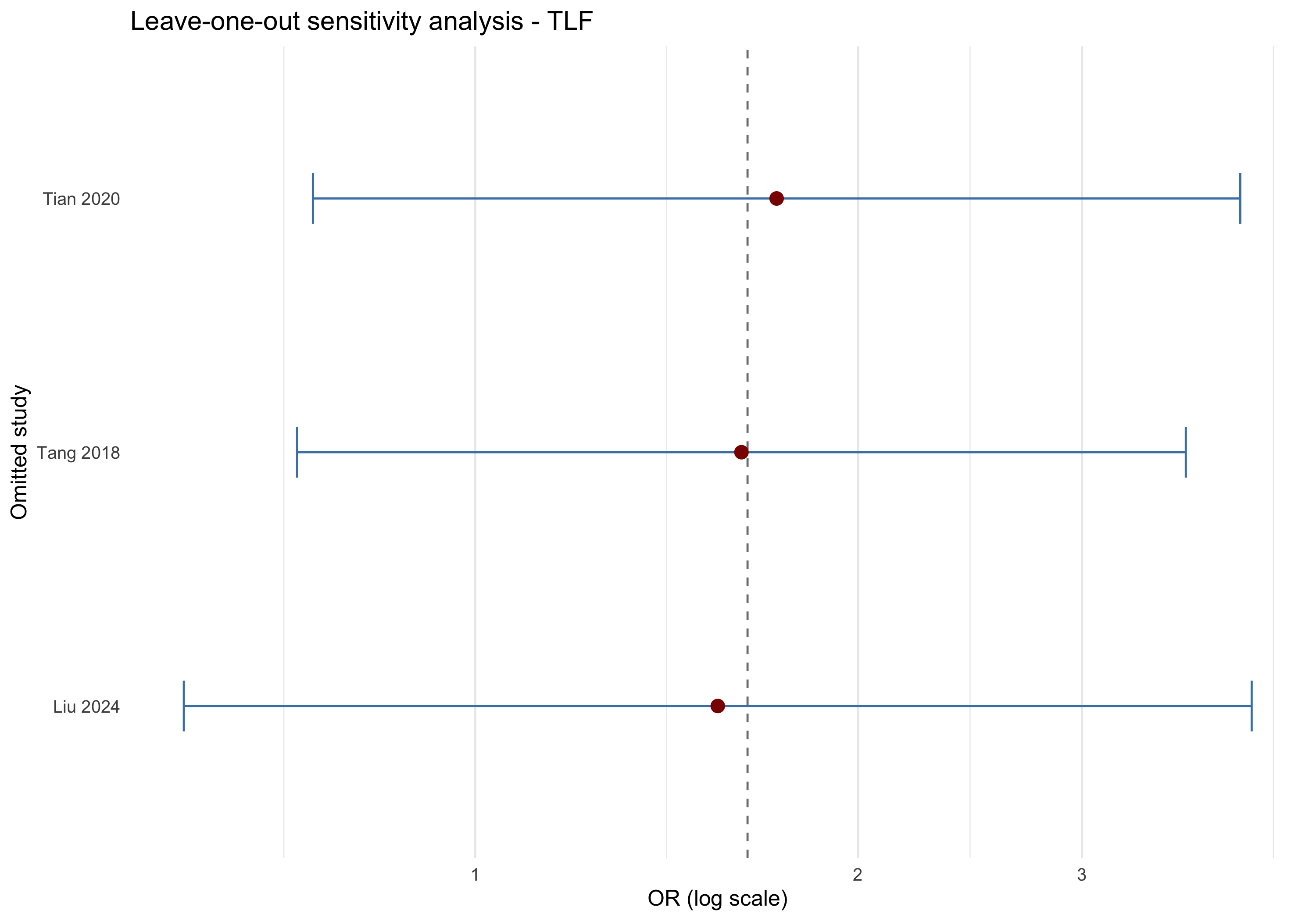

Supplement: Supplementary file 5 [file Datasheet1.zip › R scripts/12/TLF_sensitivity_leave1out.tiff]

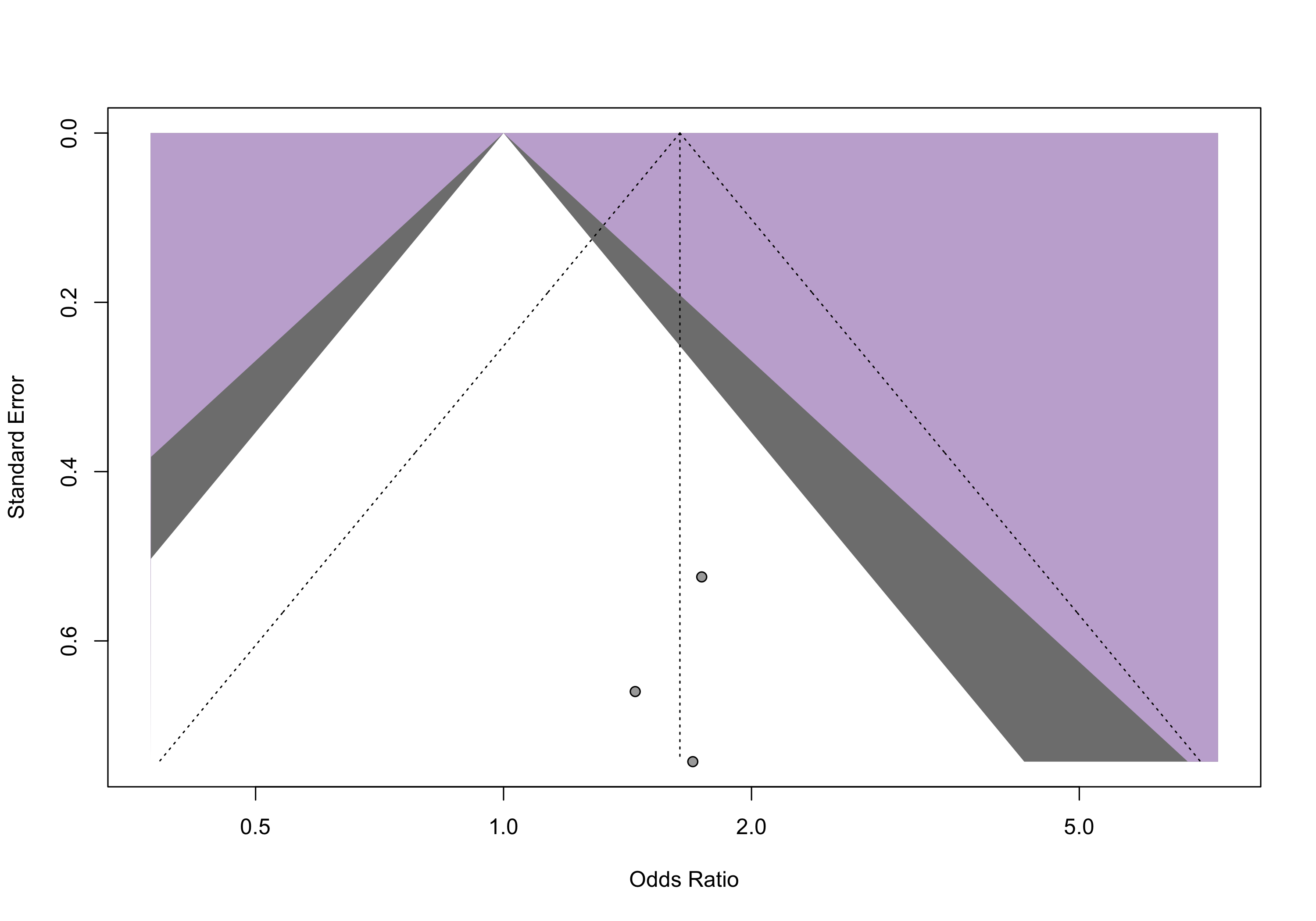

Supplement: Supplementary file 5 [file Datasheet1.zip › R scripts/12/TLF_funnel.tiff]

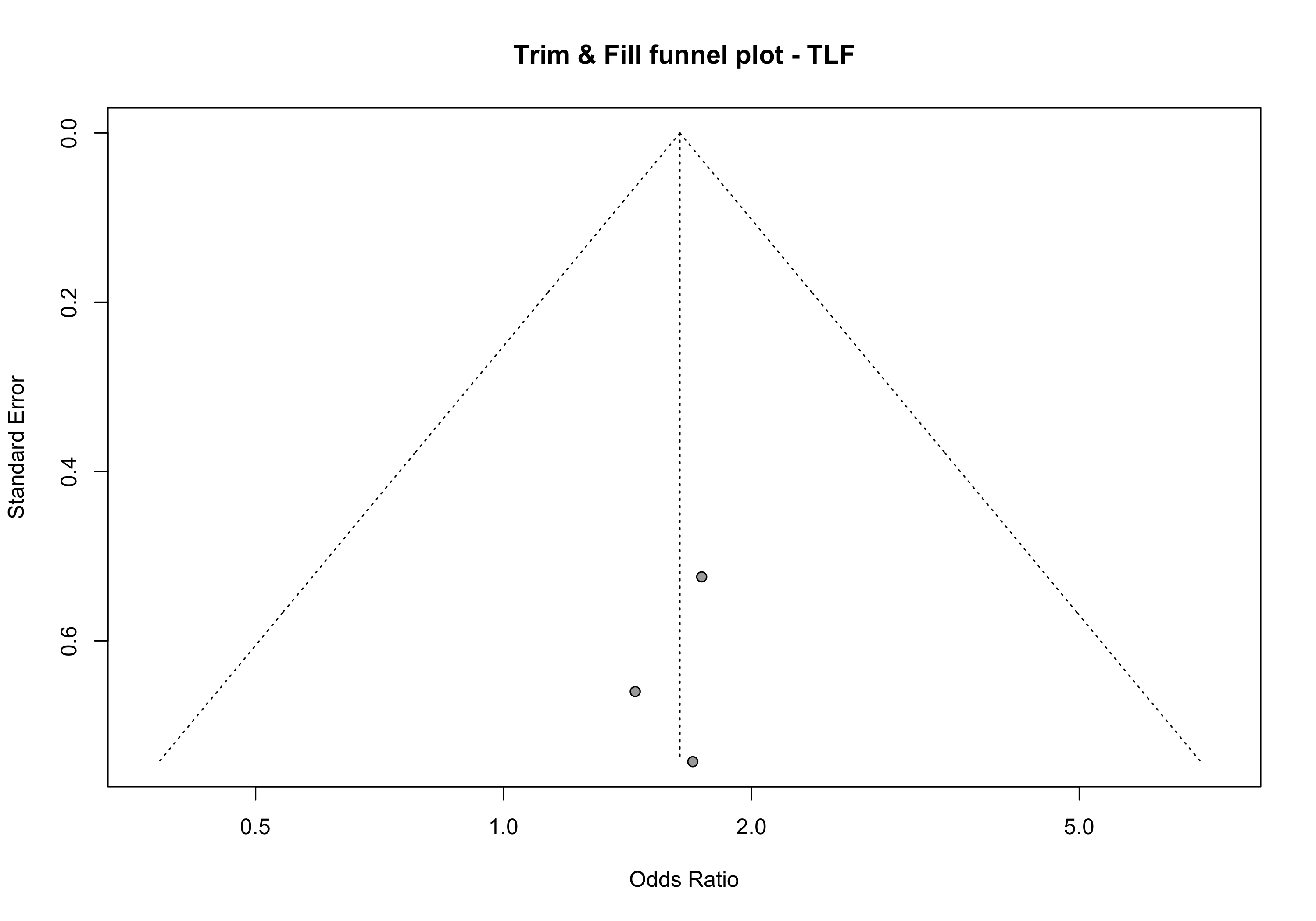

Supplement: Supplementary file 5 [file Datasheet1.zip › R scripts/12/TLF_trimfill_funnel.tiff]

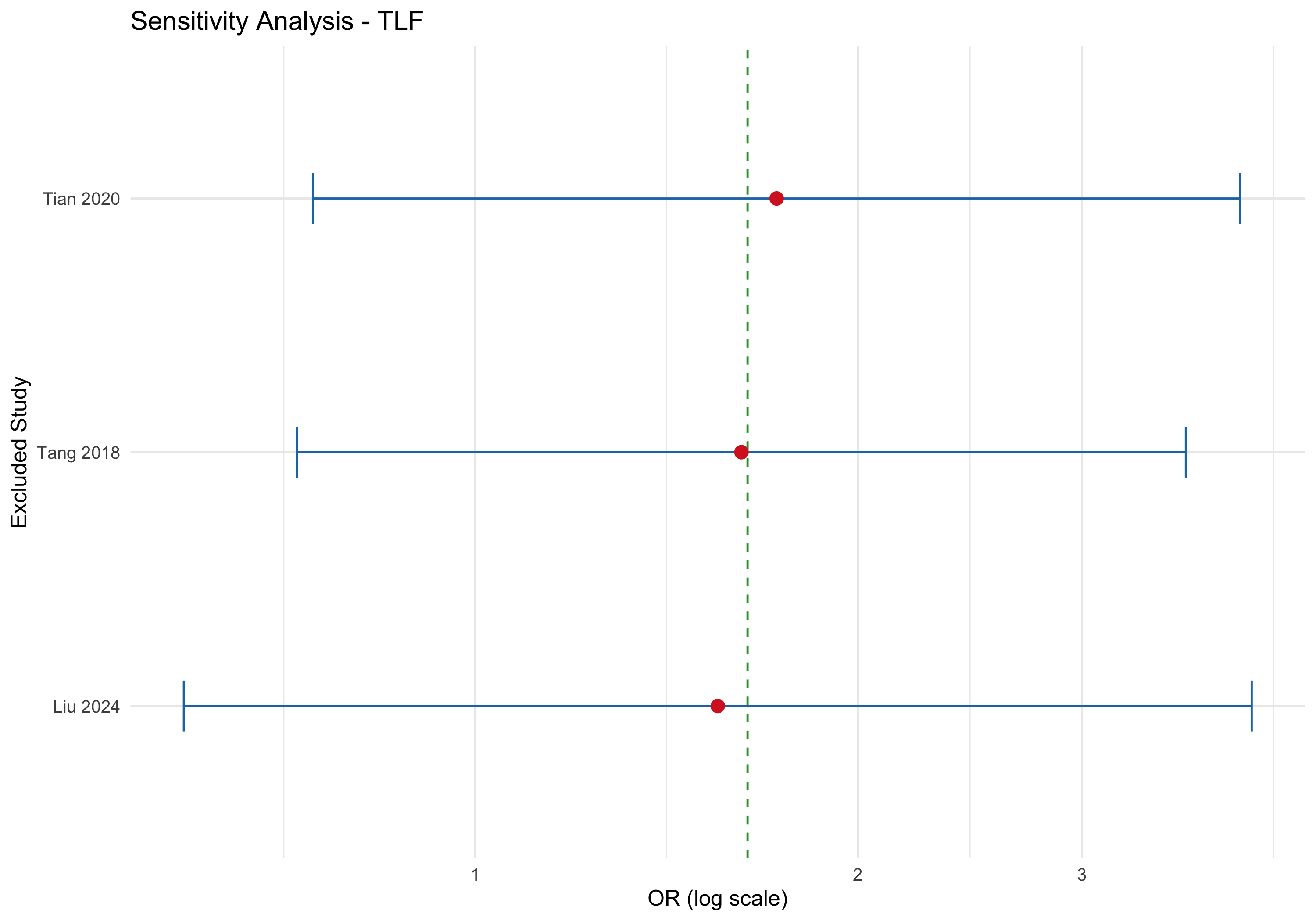

Supplement: Supplementary file 5 [file Datasheet1.zip › R scripts/12/TLF_sensitivity.tiff]

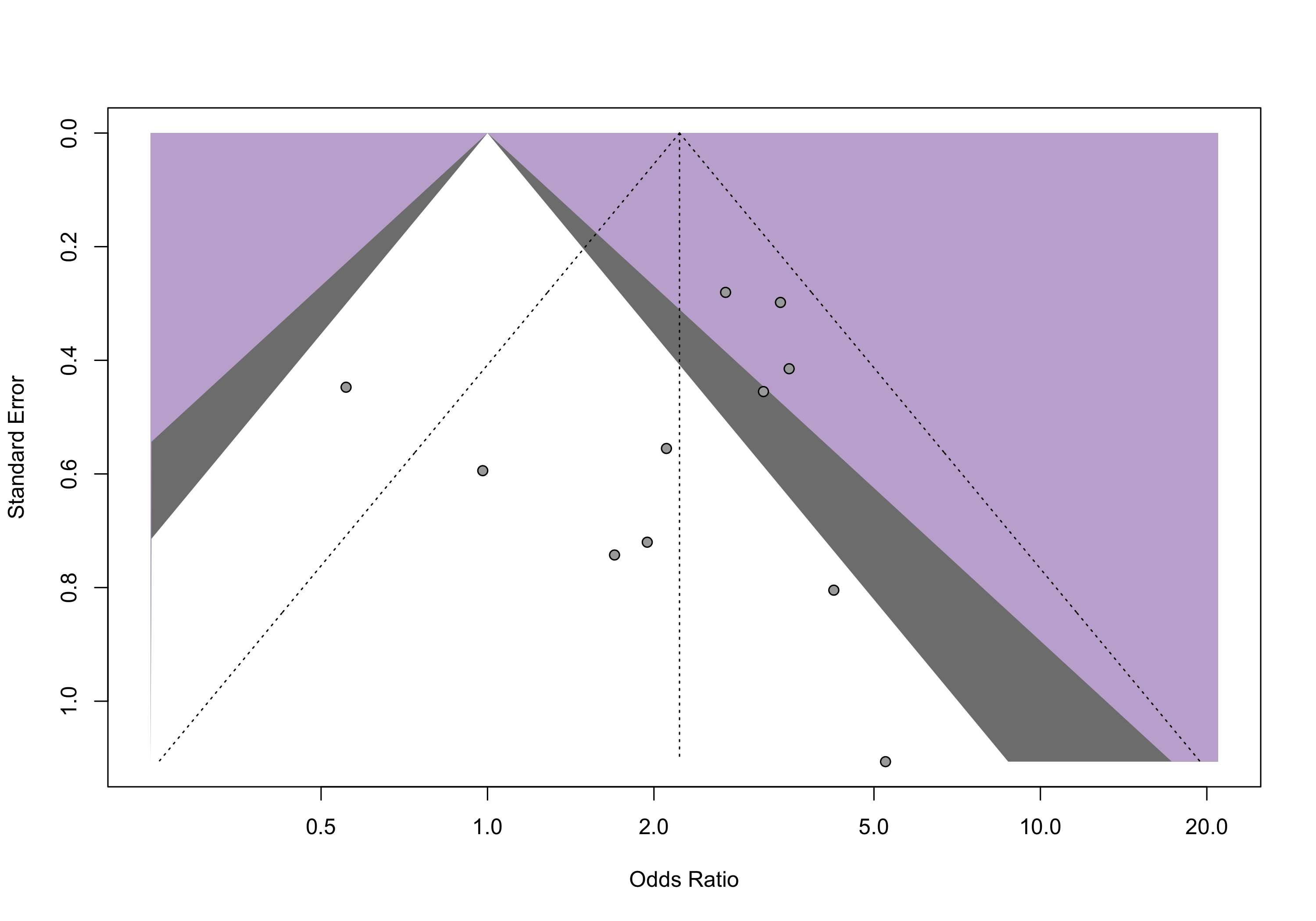

Supplement: Supplementary file 5 [file Datasheet1.zip › R scripts/5/TLR_funnel.tiff]

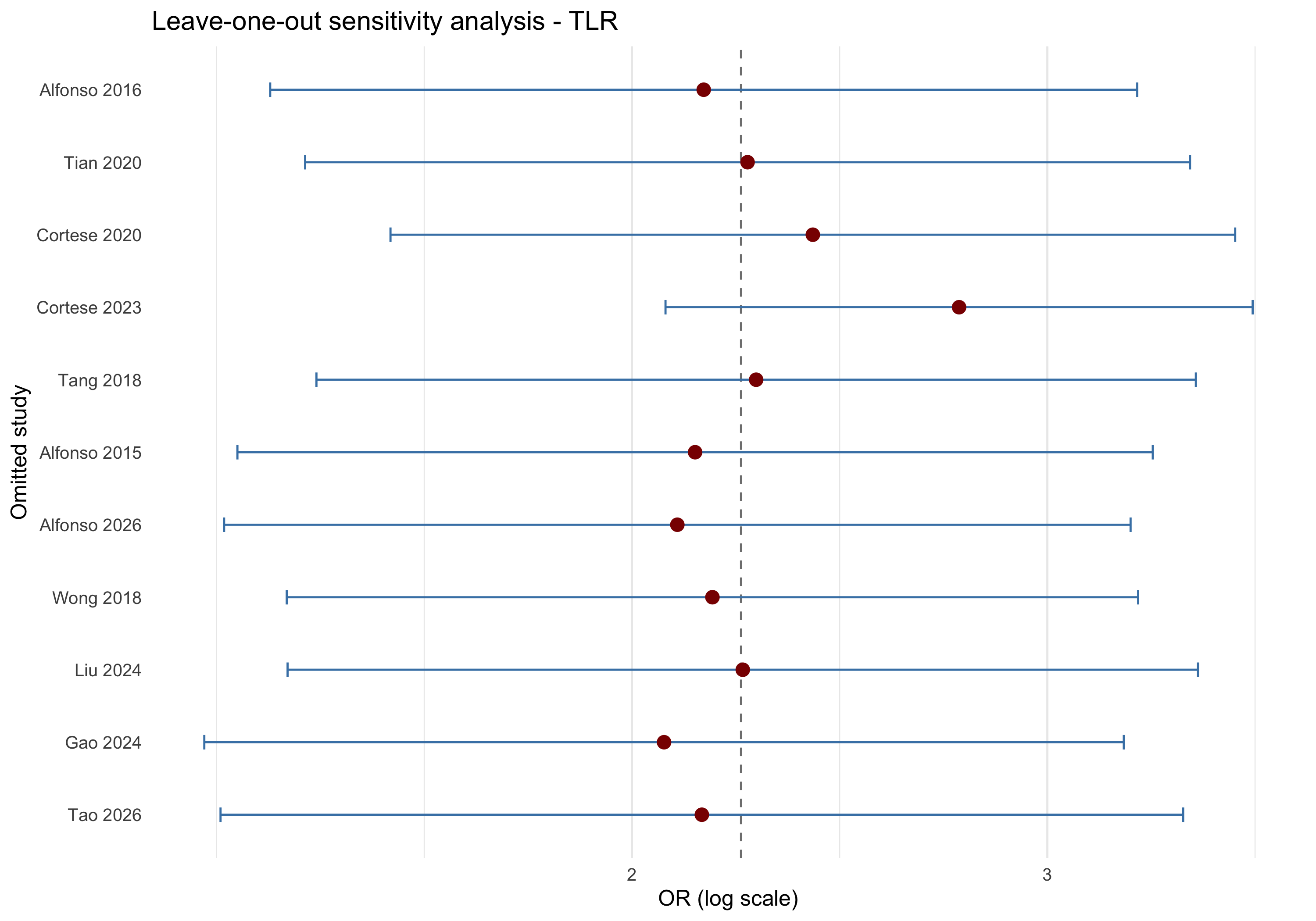

Supplement: Supplementary file 5 [file Datasheet1.zip › R scripts/5/TLR_sensitivity_leave1out.tiff]

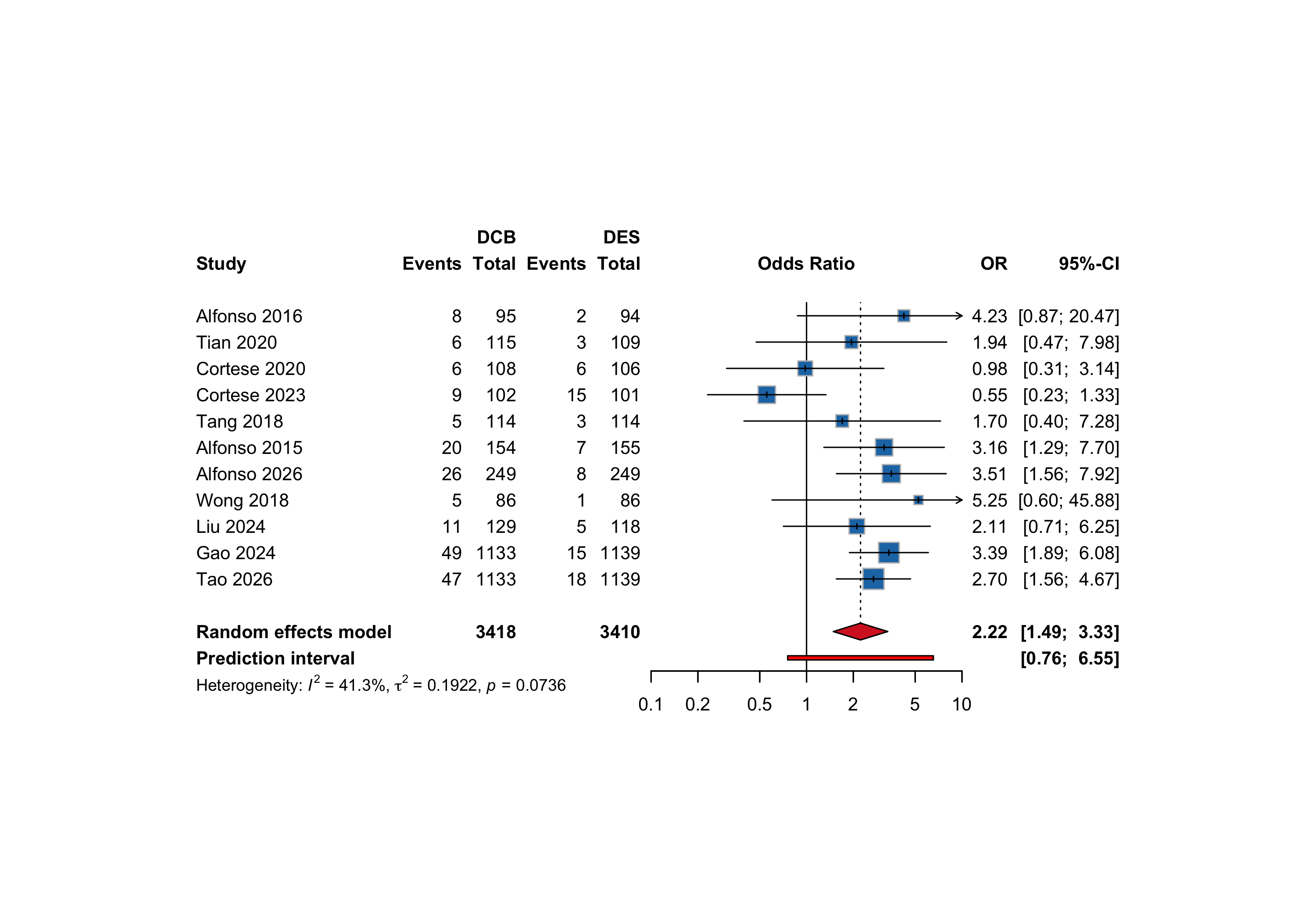

Supplement: Supplementary file 5 [file Datasheet1.zip › R scripts/5/TLR_forest.tiff]

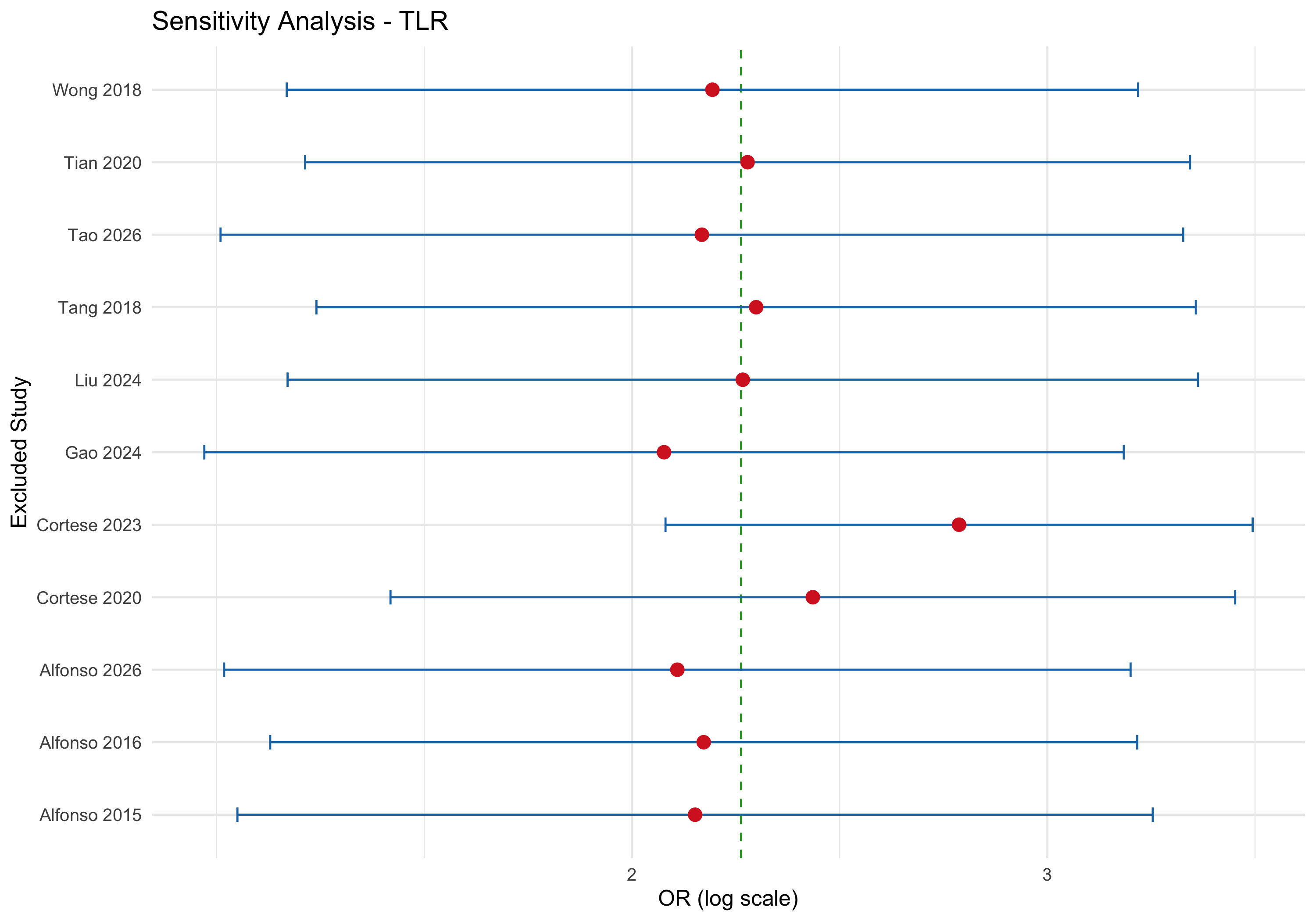

Supplement: Supplementary file 5 [file Datasheet1.zip › R scripts/5/TLR_sensitivity.tiff]

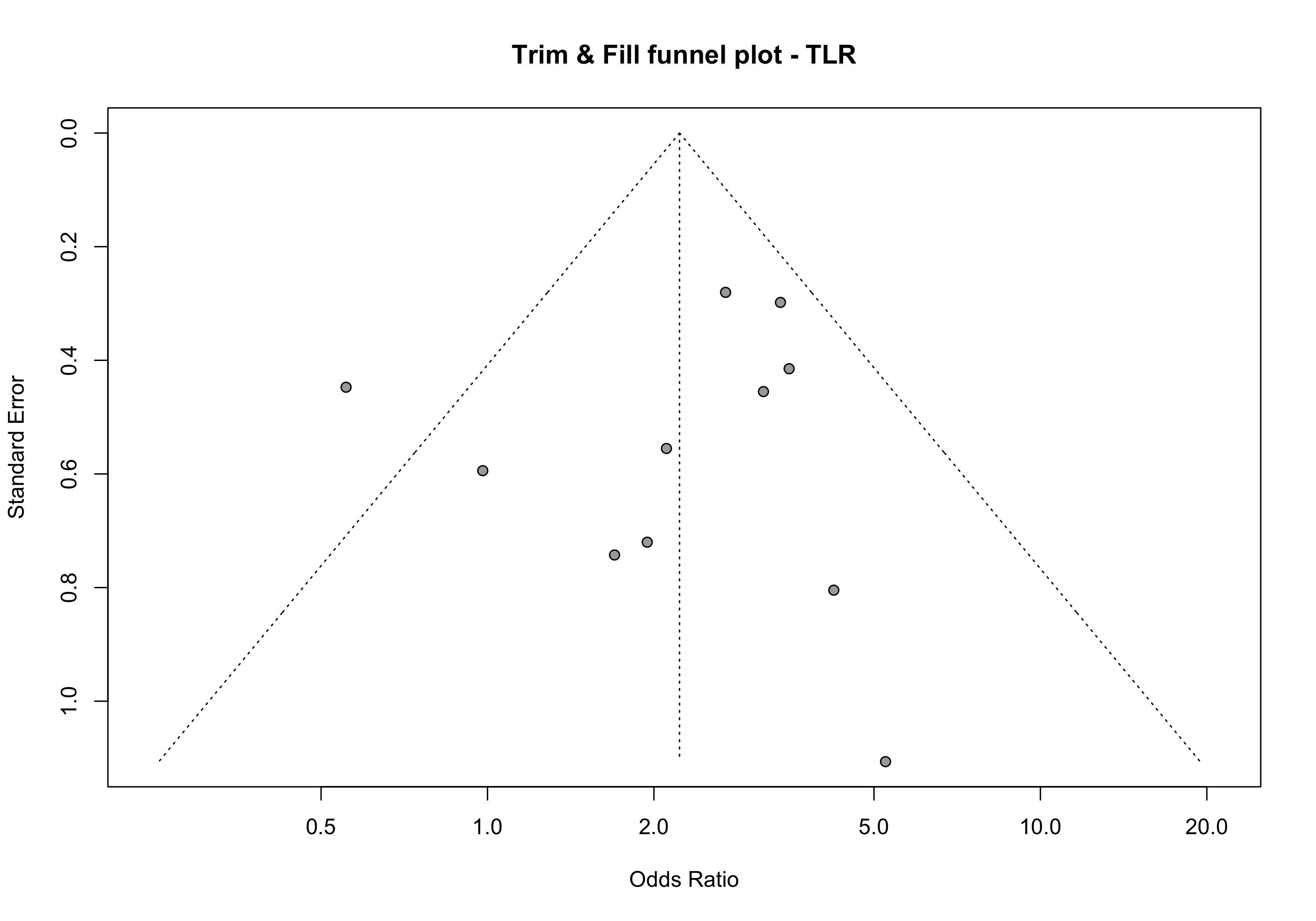

Supplement: Supplementary file 5 [file Datasheet1.zip › R scripts/5/TLR_trimfill_funnel.tiff]

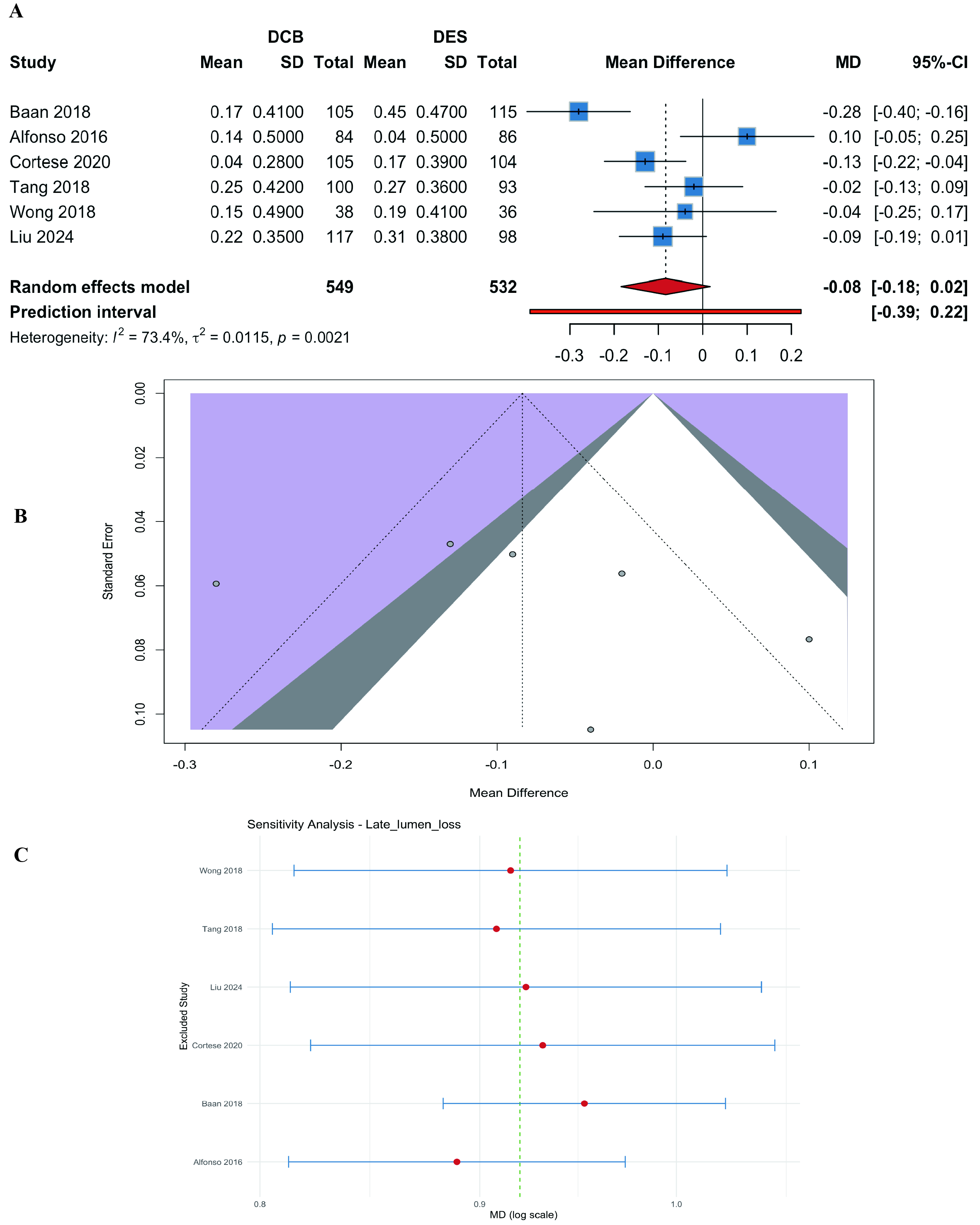

Supplement: Supplementary file 6 [file Image1.tif]

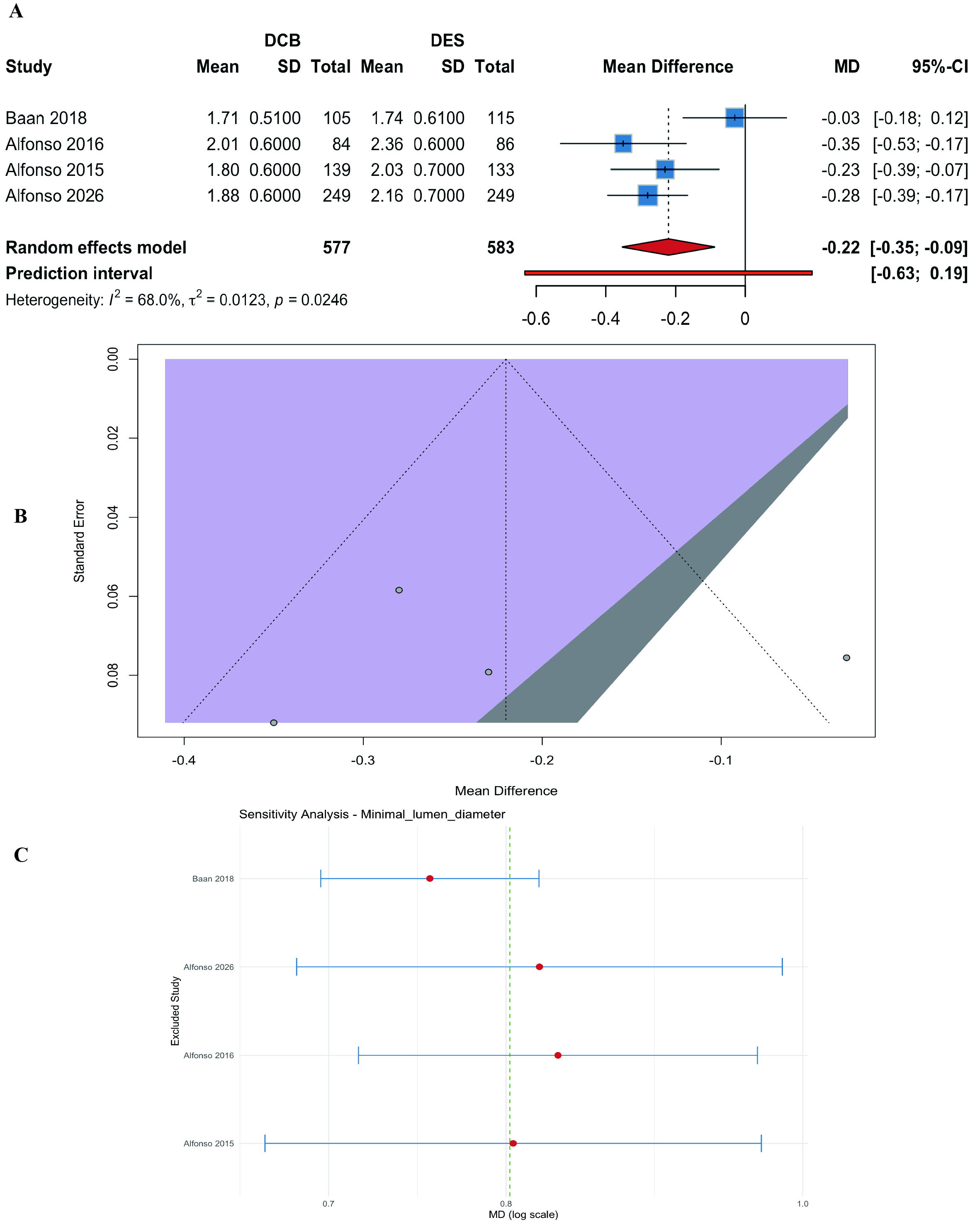

Supplement: Supplementary file 7 [file Image2.tif]

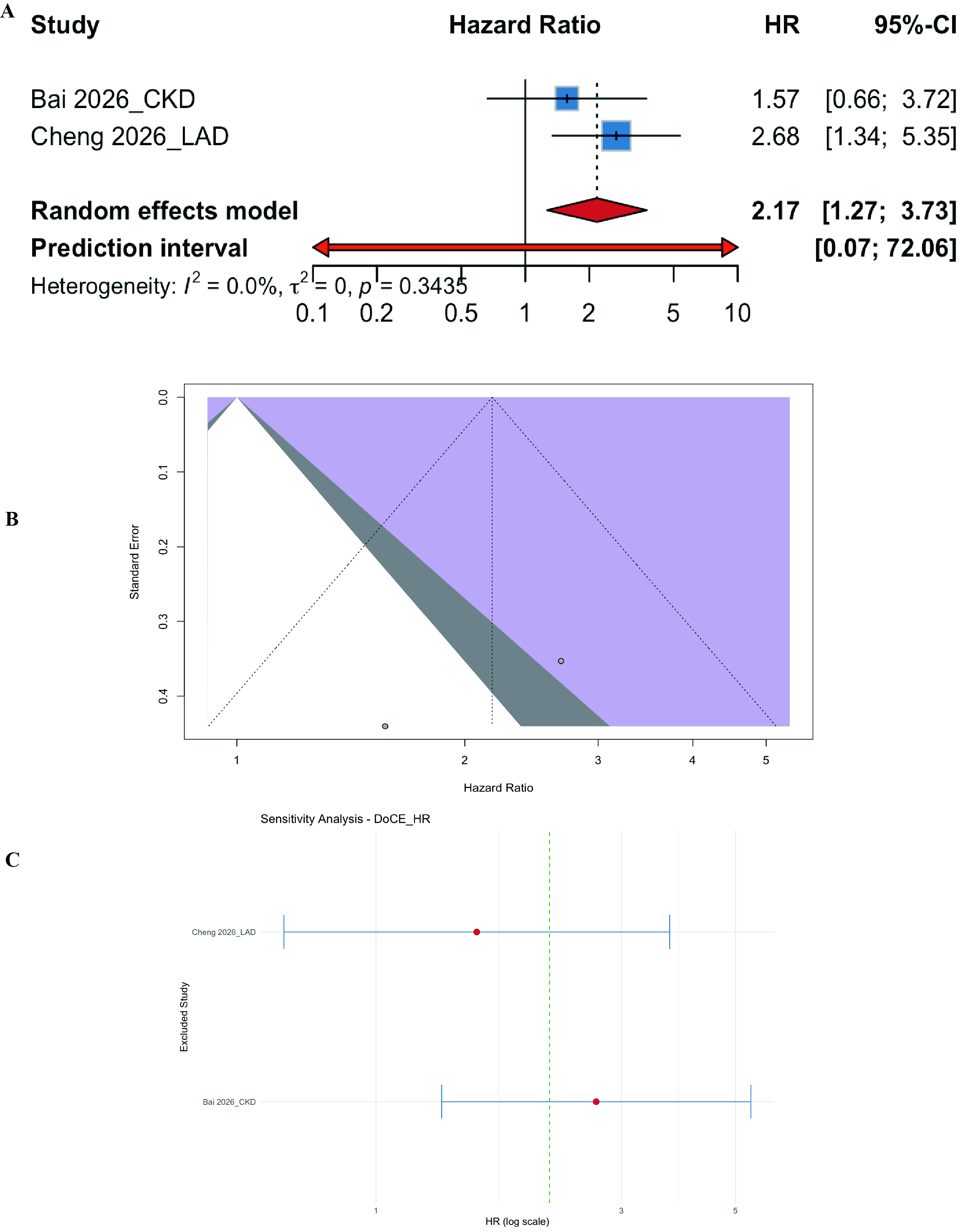

Supplement: Supplementary file 8 [file Image3.tif]

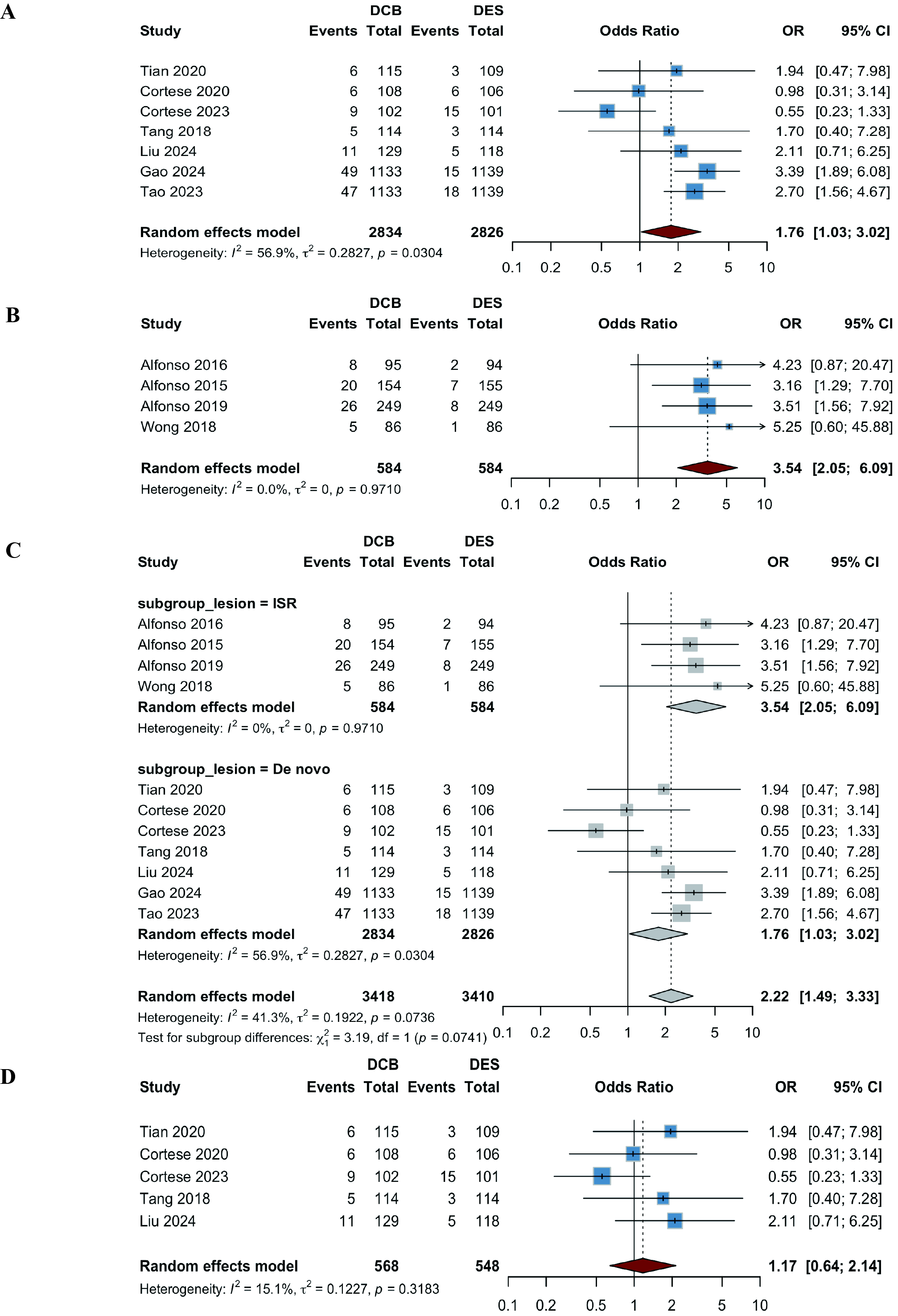

Supplement: Supplementary file 9 [file Image4.tif]
